# Structure‐Based Design, Synthesis, and Biological Evaluation of Oxadiazole–Morpholine Hybrids as Potent PARP‐1 Inhibitors Inducing Apoptosis in Breast Cancer Cells

**DOI:** 10.1002/ddr.70346

**Published:** 2026-07-08

**Authors:** Nader R. Albujuq, Khaled M. Darwish, Sherif Ashraf Fahmy, Buthaina Hussein, Ghada G. Kayed, Musa A. Said, Mohamed S. Nafie

**Affiliations:** ^1^ Department of Chemistry, School of Science The University of Jordan Amman Jordan; ^2^ Medicinal Chemistry Department, Faculty of Pharmacy Suez Canal University Ismailia Egypt; ^3^ Department of Medicinal Chemistry, Faculty of Pharmacy Galala University New Galala Egypt; ^4^ Department of Pharmacy, Institute of Pharmaceutics and Biopharmaceutics Marburg University Marburg Germany; ^5^ Department of Pharmacy, Faculty of Pharmacy Al‐Zaytoonah University of Jordan Amman Jordan; ^6^ Faculty of science Zarqa University Zarqa Jordan; ^7^ Department of Chemistry, Faculty of Science Islamic University of Madinah Madinah Saudi Arabia; ^8^ Department of Chemistry, College of Sciences University of Sharjah Sharjah United Arab Emirates; ^9^ Bioinformatics and Functional Genomics Research Group, Research, Institute of Sciences and Engineering (RISE) University of Sharjah Sharjah United Arab Emirates; ^10^ Chemistry Department Faculty of Science, Suez Canal University Ismailia Egypt

**Keywords:** apoptosis induction, breast cancer, oxadiazole, PARP‐1 inhibition, synthesis

## Abstract

Poly(ADP‐ribose) polymerase‐1 (PARP‐1) plays a central role in the repair of DNA single‐strand breaks and represents an established therapeutic target in cancer treatment. In this study, a series of novel oxadiazole–morpholine hybrid compounds was designed and synthesized using a structure‐based drug design approach to target the catalytic domain of PARP‐1. The synthesized compounds were evaluated for their cytotoxic activity against breast and ovarian cancer cells, PARP‐1 inhibitory potency, and apoptosis‐inducing effects. Among the tested compounds, **12a** exhibited potent cytotoxic activity against MDA‐MB‐231 cells (IC_50_ = 1.23 μM), surpassing the reference drug olaparib (IC_50_ = 3.45 μM). Compound **12a** also demonstrated strong PARP‐1 inhibitory activity (IC_50_ = 0.034 μM), comparable to olaparib (IC_50_ = 0.012 μM). Flow cytometric analyses revealed that compound **12a** significantly induced apoptotic cell death and altered cell cycle progression. Molecular docking studies suggested plausible binding interactions within the PARP‐1 catalytic site, supporting the observed biological activity. These findings identify oxadiazole–morpholine hybrids as promising scaffolds for further optimization as PARP‐1 inhibitors.

## 1 Introduction

Cancer remains a serious global health challenge that affects individuals of all ages in nearly all human populations. It is the second most prevalent cause of death throughout the world and still makes a significant contribution to high mortality rates and increasing healthcare burden. According to the World Health Organization (WHO) and Global Cancer Observatory (GLOBOCAN**)**, in 2024, an estimated 20 million new cancer cases were recorded globally, with around 10 million deaths caused by disease‐related factors. This is a concerning indicator of the continued rise in cancer incidence, despite advances in prevention and early detection. The growing burden of cancer presents a significant public health challenge, not only due to its increasing incidence but also because of its biological diversity, and this complexity hinders accurate diagnosis and limits the effectiveness of many therapeutic approaches (Arnold 2015)

Cancer comprises over 200 distinct types, each with unique tissue‐level variations that complicate diagnosis and treatment. Current therapies, such as chemotherapy, radiation, and surgery, aim to target malignant cells without affecting normal and healthy cells, but treatment efficacy remains limited due to cancer's multifactorial nature (Bray 2024)

Recently developed cancer drugs target specific molecular pathways, and one of the most promising targets is the enzyme poly (ADP‐ribose) polymerase 1 (PARP‐1) (Hopkins 2019). These specific enzymes play a key role in detecting and repairing DNA strand breaks and damage (Kanev 2024). PARP‐1 binds to the break sites using zinc finger motifs and catalyzes the addition of ADP‐ribose units from NAD^+^ to itself and other proteins. This process results in chromatin relaxation, allowing the DNA repair machinery to access and repair DNA lesions (Wang 2017).

While PARP‐1 activity plays a critical role in maintaining genomic integrity during minor DNA damage, its overactivation in response to extensive damage can trigger cell death via apoptosis or necrosis. Furthermore, dysregulated PARP‐1 expression, either loss or overexpression, is associated with carcinogenesis (Ito 2016). Under expression impairs DNA repair and leads to genomic instability, while overexpression has been observed in various aggressive tumors such as breast, lung, and melanoma cancers. This elevated expression is typically associated with a poor clinical prognosis and diminished responsiveness to therapy (Jain et al. 2025).

**Figure 1:**
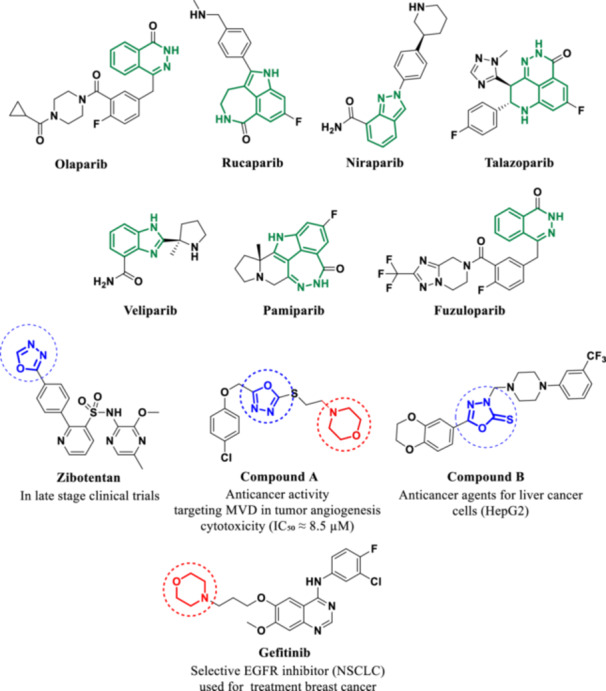
Structures of FDA‐approved PPAR‐1 inhibitors, second wave of inhibitors, and reported anti‐cancer compounds containing an oxadiazole and/or morpholine tethered residue.

Given these functions, PARP‐1 inhibitors have emerged as valuable therapeutic agents in cancer treatment. Inhibition of PARP‐1 by these agents impairs DNA repair mechanisms in cancer cells, with minimal impact on normal cells (Slade 2020). This strategy emphasizes the efficacy of PARP‐1 inhibitors as a molecular approach to combating cancer (Bondar and Karpichev 2024). To date, the clinical landscape of PARP‐1 inhibition is defined by several FDA‐approved drugs, including Olaparib (2014), Rucaparib (2016), Niraparib (2017), and Talazoparib (2018), alongside newer regional approvals such as Veliparib, Pamiparib, and Fuzuloparib (also referred to as Fluzoparib) (Figure 1). While these agents have revolutionized treatment for Breast Cancer gene (BRCA)‐mutated cancers, they are primarily based on phthalazinone (Olaparib), indazole (Niraparib), or tricyclic benzimidazole (Rucaparib) scaffolds (Wang 2026). A significant safety concern with these agents is their lack of isoform selectivity; profiling studies show that many clinical inhibitors exhibit polypharmacology, potently inhibiting other PARP family members such as PARP2, PARP3, and the tankyrases (PARP5a/b). The absence of selectivity results in narrow therapeutic windows and common hematologic adverse events, including severe anemia and neutropenia in up to 80% of patients (Wang 2025).

Furthermore, their heterogeneous mechanical profiles impact on the clinical utility of these agents. For instance, Veliparib (ABT‐888) is a potent dual PARP‐1/2 inhibitor (IC_50_ = 5.2 and 2.9 nM, respectively) with low PARP‐trapping activity due to steric hindrance. As such, it has shown limited activity as monotherapy but has shown considerable success in combination of regimen with chemotherapy. On the other hand, regional entries like Pamiparib and Fuzuloparib have demonstrated high response rates in BRCA‐mutated populations (e.g., 61.9% Overall Response Rate for Pamiparib in ER+/HER2− cohorts) and favorable pharmacokinetic profiles, with manageable hematologic safety concerns (Zeng 2024). Despite these advances, the development of compounds with promising chemical scaffolds has been identified as an efficient approach to identify relevant hits for further development and optimization (De Oliveira 2012).

The 1,3,4‐oxadiazole ring is a well‐known pharmacophore in medicinal chemistry and has been reported to possess a wide range of biological activities, particularly anti‐cancer activity (Glomb et al. 2018). It is better than other oxadiazole isomers because of its physicochemical advantages like improved metabolic stability and enhanced aqueous solubility and reduced lipophilicity (Ahsan 2022). The 1,3,4‐oxadiazole moiety is a bioisostere of carbonyl‐containing groups (e.g., esters, amides, carbamates) and therefore contributes to improved ligand‐target interactions (Verma 2019). It also serves as a planar aromatic linker that gives an optimal spatial orientation of pharmacophores and can lead to an improved binding affinity and specificity (Stecoza 2021).

It is worth noting that none of the currently approved PARP‐1 inhibitors utilize the 1,3,4‐oxadiazole ring. On the other hand, several oxadiazole‐based compounds have demonstrated clinical and preclinical efficacy, where such pharmacophores have already clinically validated in oncology through agents like Zibotentan, an endothelin receptor antagonist (Shepard and Dreicer 2010; James and Growcott 2009). Another key pharmacophoric feature in drug design is the morpholine moiety, which has been widely incorporated into various biologically active compounds due to its favorable pharmacokinetic and pharmacodynamic properties (Duan 2022; Mahal 2019, 2017). Morpholine has been extensively incorporated into numerous derivatives explored for their anticancer, antiviral, anti‐inflammatory, and antitubercular activities (Araki 2012). Various modified natural products and synthetic chemical scaffolds with a morpholine ring, such as Gefitinib, displayed potent cytotoxic activities (Araki 2012). Notably, a novel class of synthesized oxadiazole–morpholine hybrid compounds have been evaluated against Dalton's Lymphoma Ascites (DLA) tumor cells. Among them, Compound **A** showed significant in vitro cytotoxicity (IC_50_ ≈ 8.5 µM) and achieved an 85% tumor volume reduction in vivo (Figure 1). This dual mechanism of action illustrates the potential of oxadiazole–morpholine derivatives. It supports their further investigation into anticancer drug development (Al‐Ghorbani et al. 2015). Compound **B,** as well, showed potent antitumor activity, demonstrated strong focal adhesion kinase (FAK) inhibitory activity with an IC_50_ of 0.78 µM (Sun 2017).

Inspired by these findings, our recent research efforts have focused on the design and synthesis of hybrid compounds combining 1,3,4‐oxadiazole and morpholine moieties (Boström 2012), supported by molecular modeling to optimize molecular interactions and inhibit PARP‐1 biotarget. The rationale for this approach is to exploit the synergistic potential of both pharmacophores, thereby enhancing overall anticancer activity while improving drug‐like properties. These oxadiazole–morpholine hybrid compounds hold great promise in overcoming the structural and safety limitations of existing PARP‐1 clinical therapies.

## 2 Results and Discussion

### 2.1 Scientific Rational

To design and synthesize a new class of compounds with significant PARP‐1 inhibitory activity, we adopted a combined ligand‐ and structure‐based drug design approach. Design of our compounds was guided by selecting suitable chemical synthons that satisfied the binding interactions, topology, and electrostatic characteristics of the PARP‐1 active site. Further, exploring the common pharmacophoric features of reported PARP‐1 inhibitors and adopting them in the design of our compounds has been considered beneficial for circumventing challenges related to both compounds’ dynamics and kinetics. In these regards. Combining both ligand‐ and structure‐based approaches would increase success rates across rigorous preclinical and clinical testing.

**Figure 2:**
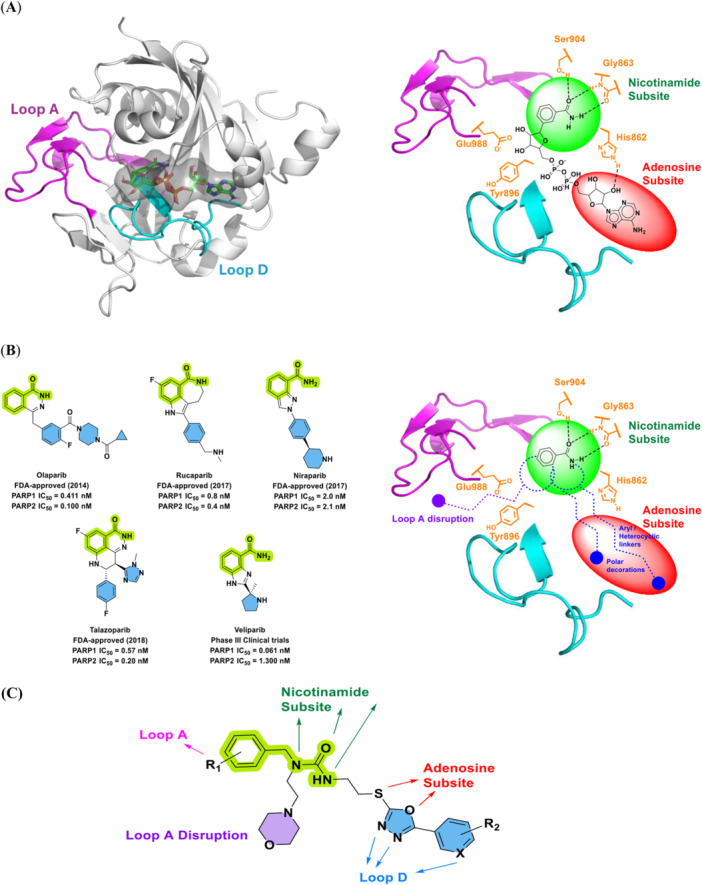
Scientific rational for designing our benzyl‐urea derivatives with tethered morpholine‐ and 1,3,4‐oxadiazole‐based linkers. (A) Structure features of the *C*‐terminal ADP‐ribosyltranferase folding at PARP‐1 enzyme (PDB: 5NQE), highlighting the main secondary protein structures involved in substrate binding, recognition, and enzyme catalysis. Right panel shows the key binding interactions between a non‐hydrolyzable NAD^+^ analog and PARP's catalytic and binding residues. Subsites for nicotinamide and adenosine are color‐coded; (B) FDA‐approved and clinical candidates of PARP‐1/2 small molecule inhibitors depicting nicotinamide bioisosteres and hydrophobic tails for dual accommodation of the nicotinamide and adenosine subsites. Suggested approach for tampering PARP‐1 selectivity through disrupting Loop A and Loop D conformational shift for inducing relevant pocket of selectivity; (C) Schematic architecture of our proposed PARP‐1 inhibitors based on benzyl‐urea, 1,3,4‐oxadiazole, and morpholine scaffolds for mediating relevant interactions to residues of both nicotinamide and adenosine subsites. Postulated compound‐PARP‐1 interactions are represented as solid arrows.

Human PARP‐1 pocket compromises the highly conserved catalytic domain among other PARP family members. This pocket is recognized as the *C*‐terminal ADP‐ribosyltransferase folding, which permits the binding of NAD^+^ in an optimal conformation for catalyzing the ADP‐ribosylation (PARylation) post‐translational modification of substrate proteins (Kouyama 2019). The pocket resides in the space defined by two loops: loop A (acceptor), which binds directly to the protein substrate to be PARylated, and loop D (donor), which interacts directly with the NAD^+^ molecule (Figure 2A). This pocket further comprises two distinct subsites for anchoring the nicotinamide and adenosine moieties of the NAD^+^ molecule, respectively (Langelier 2018). Almost all PARP‐1 inhibitors bind to this pocket, mimicking the interactions between the enzyme pocket and the nicotinamide moiety of NAD^+^ molecules (Rudolph et al. 2022). Typically, there are two conserved polar contacts between the nicotinamide group and the PARP‐1's conserved residues, Gly863 and Ser904, towards their main chain and side chain, respectively. Further, the catalytic triad His862, Tyr896, and Glu988 resides at the catalytic site, vicinal to the nicotinamide pocket, which is essential for driving PARP‐1's catalytic activity (Zhu 2023). Notably, His862 is located midway between the nicotinamide and adenosine subsite, where it permits polar hydrogen bonding towards the hydroxyl group of the adenosine moiety at C2 as well as hydrophobic π‐stacking with the aromatic nicotinamide group (Hottiger 2010).

Several FDA‐approved and clinical‐phase PARP‐1 inhibitors exhibit the structural and pharmacophoric features required to target both the nicotinamide and adenosine subsites of the catalytic enzyme (Figure 2B) (Deeks 2015; Syed 2017; Heo and Duggan 2018; Hoy 2018). Despite harboring different chemical scaffolds, these agents feature nicotinamide bioisosteric groups (benzene carboxamide) that can satisfy the hydrogen‐bonding potential of the conserved residues, Gly863 and Ser904. On the other hand, the reported PARP‐1 inhibitors displayed a long hydrophobic tail comprising several connected aromatic/heteroaromatic rings, directed toward the adenosine subsite. Besides driving potential π‐contacts, these multi‐ring scaffolds can be decorated with polar substituents for mediating relevant polar contacts with the subsite lining residues, including the catalytic His862 side chain. Based on this reported structural architecture, we adopted the benzyl urea scaffold as an untampered bioisostere of the nicotinamide moiety, where the urea group would serve as the hydrogen‐bond warhead to mediate optimal stability at the subsite‐conserved Gly863 and Ser904. Further, our compounds feature a long hydrophobic tail with a 1,3,4‐oxadiazole ring at its center to accommodate the adenosine subsite. The rational for incorporating the 1,3,4‐oxadiazole moiety was for its well‐established favorable pharmacokinetic properties and its ability to engage in hydrogen bonding and π‐π stacking interactions with relevant pocket residues. Harboring the (N─C─O) structural fragment, 1,3,4‐oxadiazole confers increased lipophilicity to incorporated compounds, potentially boosting absorption and cell membrane permeability (Ahsan 2022). Further, the 1,3,4‐oxadiazole ring is an excellent bioisostere of the amide/ester linkage, enabling the connection of two chemical fragments along parallel synthetic pathways and providing stability against metabolic degradation (Atmaram and Roopan 2022).

Although reported PARP‐1 inhibitors targeting both nicotinamide and adenosine subsites showed potent inhibition activities, selectivity challenges arose owing to the conserved nature of the NAD^+^‐binding site among members of the PARP family (Zheng et al. 2023). Thus, approaches for targeting the less conserved secondary protein structures have been proposed as adequate for mitigating the promiscuity of the developed PARP‐1 inhibitors (Ferraris 2010). Among the PARP structural motifs, both Loops A and D are less well conserved in length and rigidity across different PARP family members where such variations impose substrate selectivity (Pinto and Schüler 2014). In this regard, the high loop A/D variability could be exploited for the design of isoform‐selective inhibitors. A recognized approach has been proposed: introducing tethered moieties to disrupt either PARP‐1 loop, thereby creating unique pockets outside the nicotinamide/adenosine subsites and generating potent, selective PARP inhibitors. This has been recognized with a potent selective *N*‐aryl piperazine‐based PARP‐14 inhibitor (PDB: 5NQE), which typically resides at the nicotinamide subsite, yet its (*Z*)‐maleic amide tail protrudes towards a nascent pocket vicinal to Loop A, which resulted from the compound's induced‐fitting conformational shift of Loop D (Upton 2017).

Thus, our compound rationalized introducing a tethered morpholine‐based linker at the urea scaffold to potentially disrupt Loop A and enhance Loop D conformational flexibility. Besides the proposed pharmacodynamic advantage of the morpholine ring, this polar scaffold was also suggested to confer better solubility indices for our designed compounds, offering them an overall balanced polar/lipophilic profile for successful survival across the prospect pre‐clinical and clinical stages. Finally, other heterocyclic fragments were strategically introduced into our compound design to explore SAR and assess enhanced target affinity, physicochemical properties, and overall biological activity. A summary of the typical architecture of our designed compounds is shown in Figure 2C.

### 2.2 Chemistry

**Scheme 1:**
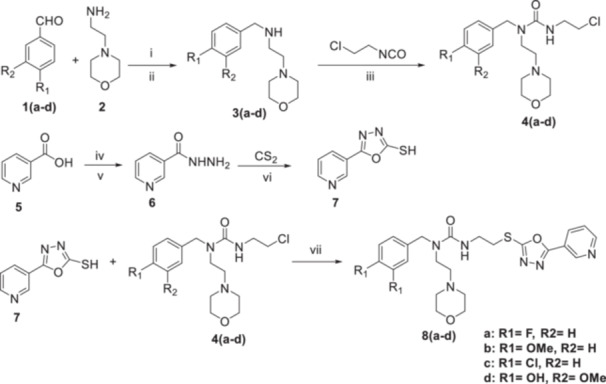
Reagents and conditions: (i) AcOH, isopropanol, r.t, 2.0 h, (ii) NaBH_4_, r.t, 24 h (iii) CH_2_Cl_2_, r.t, 1 h.; (iv) CH_3_OH, H_2_SO_4_, 80°C, 6 h (v) NH_2_NH_2_, EtOH, 80°C, 6 h (vi) DMF, KOH then HCl; (vii) Et_3_N, CH_2_Cl_2_, r.t, 1 h.

**Scheme 2:**
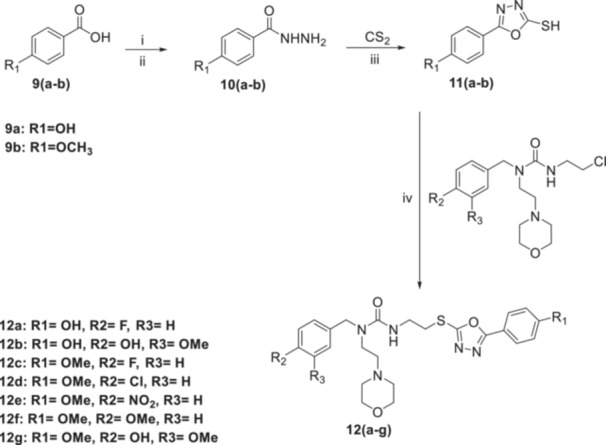
Reagents and conditions: (i) CH_3_OH, H_2_SO_4_, 80°C, 6 h (ii) NH_2_NH_2_, EtOH, 80°C, 6 h (iii) DMF, KOH then HCl; (iv) Et_3_N, CH_2_Cl_2_, r.t, 1 h.

**Scheme 3:**
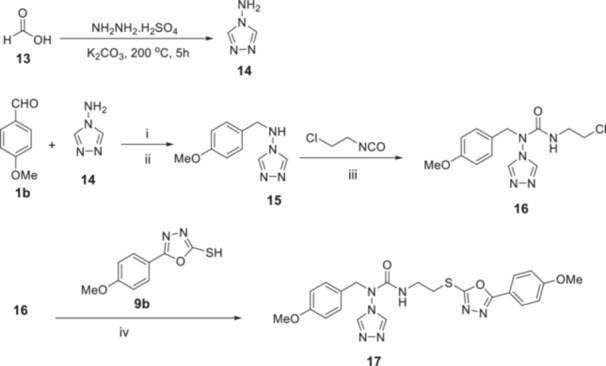
Reagents and conditions: (i) AcOH, isopropanol, r.t, 2.0 h, (ii) NaBH_4_, r.t, 24 h (iii) CH_2_Cl_2_, r.t, 1 h.; (iv) Et_3_N, CH_2_Cl_2_, r.t, 1 h.

The synthetic routes of newly designed target compounds are depicted in Schemes 1, 2, 3. Diverse scaffolds of 1,3,4‐oxadiazole moieties tethering pyridine, *p*‐methoxybenzene, and phenol rings linked with appropriate secondary amines through sulfur atom and a linker were designed, synthesized, and characterized for biological evaluation.

As depicted in Scheme 1, initially, 4‐(2‐aminoethyl)morpholine **2** underwent reductive amination with benzaldehyde derivatives **1**(**a**–**d**) to produce the corresponding secondary amines **3**(**a**–**d**). Direct coupling of chloroethyl isocyanate with secondary amines **3**(**a**–**d**) yielded chloroethylcarbamides **4**(**a**–**d**) intermediates in good yield. The 1,3,4‐oxadiazole substituent **7** was synthesized through the reaction of appropriate acid hydrazides with CS_2_ in a basic medium, followed by the addition of HCl to mediate cyclization and form the oxadiazole ring (Monte 2013; Saitoh 2009). The target compounds **8**(**a**–**d**) were successfully achieved through nucleophilic substitution between substituted 1,3,4‐oxadiazole **7** and key components **4**(**a**–**d**) (Scheme 1).

Similar approaches were applied for the synthesis of compounds **12**(**a**–**e**). The same procedure adopted for the synthesis of 1,3,4‐oxadiazoles, substituent **9** was employed. We prepared *p*‐methoxy and *p*‐hydroxy benzene ring substituents at the oxadiazole ring **11**(**a**–**b**) (Kahl 2019). The reaction between the functionalized key intermediates **4**(**a**–**e**) and compounds **11**(**a**–**b**) proceeded smoothly, and target compounds **12**(**a**–**g**) were obtained in good yields (Scheme 2).

As depicted in Scheme 3, the 4‐amino‐1,2,4‐triazole moiety **14** was incorporated into the diamine structures **15** as a nitrogen‐rich group. 4‐amino‐1,2,4‐triazole **14** was prepared according to the literature procedure (Sanz 2002). Amino triazole ring was initially condensed with appropriate aldehyde (Sultan 2020), then reduced by NaBH_4_ to produce *N*‐substituted ‐benzyl‐4*H*‐1,2,4‐triazol‐4‐amines **15.** Similarly, the efficient coupling of 1,2,4‐triazol‐4‐amines **15** with **2**‐chloroethyl isocyanate provided key intermediates **16** in high yields. The target compound **17** was successfully synthesized through the subsequent linkage of these intermediates with 1,3,4‐oxadiazoles substituent **9b** (Scheme 3).

### 2.3 Biological Analysis

Using the MTT assay, the cytotoxicity profiles of the synthesized compounds were evaluated against two cancer cell lines: the High‐grade serous ovarian (OVCAR3) and Triple‐negative breast (MDA‐MB‐231) cancer cell lines. Results are shown in Table 1 for the initial screening of cancer cell growth inhibition (GI%) at a concentration of 10 µM for the investigated compounds. Notably, the compounds’ activities were much more pronounced against the proliferation of breast over the ovarian cancer cells. Differential growth inhibition percentages for each singular compound across the two cell lines showed around a twofold at inhibiting the MDA‐MB‐231 than OVCAR3 for most compounds. The preferential targeting of the depicted cancer cell line could highlight the directed cytotoxicity activity of the synthesized compounds towards PARP‐1 inhibition, likely attributed to the reported differential PARP‐1 expressions across the tested cell lines. A study by Zhen et al. which highlighted the molecular and pathological features of different breast cancer cell lines, showed that MDA‐MB‐231 cells exhibit significantly lower basal PARP‐1 expression (Zhen et al. 2017). On the contrary, the OVCAR3 ovarian cancer cells were reported to have heterogeneous, higher detectable, and functional PARP‐1 expression (Funingana 2023). On the other hand, the positive reference standard, Olaparib, showed relatively higher activity, yet statistically insignificant, in breast cancer as compared to the ovarian ones. This could be explained by Olaparib's non‐selective nature, as this reference control has been reported to have a dual PARP‐1/2 inhibition profile with activity favoring PARP2 inhibition.

**Table 1:** Percentage of cell growth inhibition at the single dose [10 µM] for the tested compounds against MDA‐MB‐231 and OVCAR3 cancer cell lines using the MTT assay.

Code numbers	Structure	Percentage of cell growth inhibition at [10 µM] ± SD*	
		MDA‐MB‐231	OVCAR3
**8a**	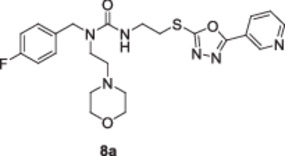	39.6 ± 1.2	36.3 ± 0.9
**8b**	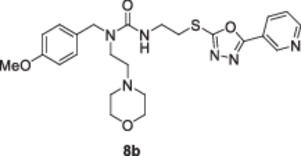	23.3 ± 0.9	12.3 ± 0.4
**8c**	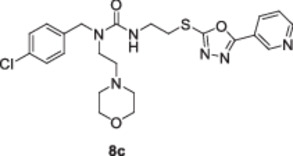	46.9 ± 1.2	16.4 ± 0.8
**8d**	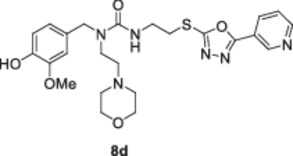	45.3 ± 1.1	21.3 ± 0.5
**12a**	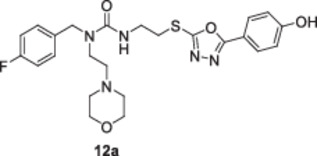	72.3 ± 2.4	59.6 ± 2.1
**12b**	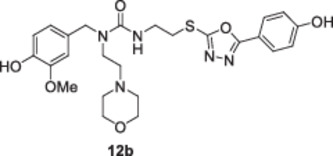	46.2 ± 1.9	41.3 ± 1.7
**12c**	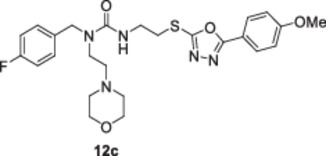	23.1 ± 1.1	23.3 ± 0.8
**12d**	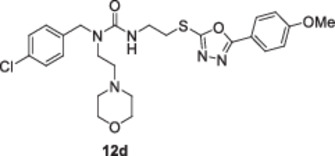	53.6 ± 2.1	19.6 ± 0.7
**12e**	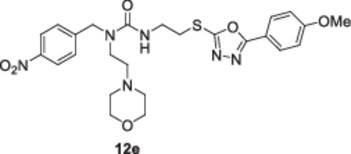	47.3 ± 1.7	16.3 ± 0.8
**12f**	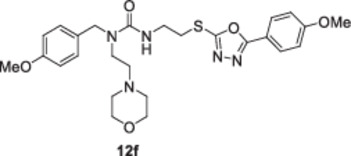	16.3 ± 0.8	3.6 ± 0.6
**12g**	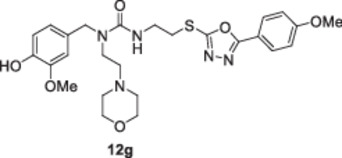	75.6 ± 2.9	63.2 ± 1.8
**17**	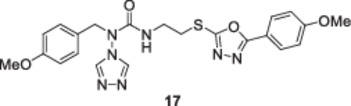	69.9 ± 2.3	61.2 ± 1.2
**Olaparib**		83.6 ± 2.7	87.6 ± 2.8

* Percentage of cell growth inhibition at a single concentration of [10 µM].

Focusing on the results for the breast MDA‐MB‐231 cell line, the growth inhibitions were more pronounced for compounds **12a**, **12g**, and **17**, reaching up to 72.3 ± 2.4, 75.6 ± 2.9, and 69.9 ± 2.3, respectively. These compounds exhibited higher cell growth inhibition in the MDA‐MB‐231 than OVCAR‐3 cells exceeding 70%. Analysis of the structure–activity relationships of the synthesized compounds against the tested breast cancer cell lines provided valuable insights into structural features associated with enhanced biological activity. Based on the structural diversity of the benzyl urea core scaffold, the presence of electron‐deactivating groups (F, Cl, and NO_2_) and/or donating groups (OH and OMe) has been associated with the terminal substitutions on the benzene ring at C2 of the oxadiazole ring. Notably, compounds with a perfect match of substituent groups to achieve a balanced polar/hydrophilic character showed higher cell growth inhibition percentages. Compound **12g** with the polar acidic OH group at the benzyl urea scaffold has been balanced with the more lipophilic group MeO benzene moiety as compared to the pyridin‐3‐yl or *para*‐tolyl synthons at the C2 oxadiazole ring. On the other hand, compounds **8a**, **12a**, and **12c** with the *para*‐fluoro substitution (highly electrophilic) on the benzyl urea scaffold were only balanced with the hydroxyl group on the terminal benzene ring at compound **12a**. Regarding less electronegative substituents on the benzene ring, compounds **8c** and **12d** bearing a *para*‐chloro substitution showed enhanced biological activity, with a greater preference for the *para*‐MeO phenyl terminal scaffold over the pyridine‐3‐yl ring (**12d** GI% > **8c** GI%). Moving towards a more electronegative substituent, compounds with terminal nitro groups showed a preference for the pyridin‐3yl ring at the other end of the compound (**8e** GI% > **12e** GI%). Finally, double MeO on both ends of the synthesized compound (as in **12f**) was highly detrimental to inhibition activity in ovarian cells, yielding the lowest GI% value (16.3 ± 0.8) among all synthesized compounds. To our surprise, just replacing the morpholine ring at **12f** with the 1,3,4‐triazole ring gave rise to one of the most highly active compounds, **17**, despite having double MeO lipophilic groups. This could suggest the beneficial impact of the 1,3,4‐triazole ring on the compound's dissolution/kinetics, as well as, to some extent, its dynamics in terms of binding interactions with prospective cancer‐associated biotargets.

**Figure 3:**
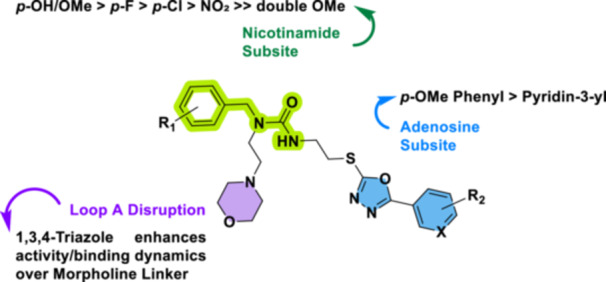
Illustrated Structure‐Activity Relationship (SAR) of oxadiazole–morpholine hybrids based on cytotoxicity against MDA‐MB‐231 and OVCAR3 cell lines. The schematic highlights the three critical pharmacophoric regions and their influence on anti‐proliferative activity.

Expanding the biological SAR results over both cancerous cells and summarizing these results have identified three primary pharmacophoric regions to impact the activity of these novel synthesized oxadiazole–morpholine hybrids. Key pharmacophoric regions involve: (i) Substituents on the benzyl‐urea core skeleton, (ii) 3,4‐oxadiazole terminal substitution, and (iii) Linker influence (Morpholine vs. Triazole) (Figure 3). Generally, the electronic nature of the benzene ring at the nicotinamide bioisostere region significantly influenced potency. The presence of a polar acidic hydroxyl group combined with a methoxy group (compound **12g**) yielded the highest GI% against MDA‐MB‐231 (75.6 ± 2.9%). Conversely, the incorporation of double methoxy groups (compound **12f**) was highly detrimental, resulting in the lowest GI% values across both cell lines (16.3% in MDA‐MB‐231% and 3.6% in OVCAR3). Highly electrophilic groups, such as the *para*‐fluoro substitution in compound **12a**, achieved optimal potency only when balanced by a hydroxyl group on the terminal benzene ring. Regarding the substitution pattern on the oxadiazole ring, the C2‐substitution served as a hydrophobic tail designed to accommodate the adenosine subsite. Scaffolds bearing a more lipophilic *para*‐methoxyphenyl moiety generally exhibited superior growth inhibition compared to those with a pyridin‐3‐yl or *para*‐hydroxybenzene ring. For example, compound **12d** (*para*‐chlorobenzyl with *para*‐methoxyphenyl) was significantly more active than its pyridine‐3‐yl analog (**8c**). Finally, replacement of morpholine ring with a 1,3,4‐triazole ring (compound **17**) resulted in a dramatic restoration of activity in scaffolds that were otherwise inactive. This suggests that the triazole ring may improve binding dynamics, possibly due to its aromatic nature favoring interactions with residues like Tyrosine. Top‐active compounds were then analyzed for cancerous cell line IC_50_ cytotoxicity assay, PARP‐1 enzymology inhibition analysis, and molecular docking simulations for gaining further insights.

**Figure 4:**
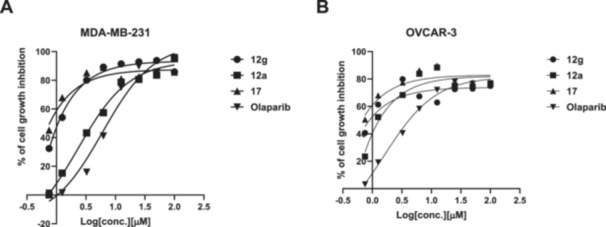
Non‐linear regression curves fit for the Dose‐response curves of the percentages of cell growth inhibition (%) versus the log values of working serial concentrations (2 to −0.12 µM) for 48 h. (A) MDA‐MB‐231 breast cancer cells, and (B) OVCAR‐3 ovarian cancer cells.

As summarized in Table 2 and Figure 4, for the IC_50_ calculation, compounds **12g, 12a,** and **17** exhibited potent inhibitory activity against MDA‐MB‐231 cancer cells. **12a** showed potent cytotoxicity against MDA‐MB‐231 cells, with an IC_50_ of 1.23 ± 0.1 µM, compared to Olaparib, which had an IC_50_ of 3.45 ± 0.2 µM. Furthermore, compounds **12g** and **17** showed potential cytotoxicity against MDA‐MB‐231 cancer cells, with IC_50_ values of 10.7 ± 0.9 and 12.3 ± 1.1 µM, respectively. Conversely, MCF‐10A normal cells did not exhibit cytotoxicity against any of the compounds, even those with higher IC_50_ values.

**Table 2:** IC_50_ values of 12a, 12g, and 17 against MDA‐MB‐231cancer cells.

Compound	IC_50_ [µM] ± SD^a^	
	MDA‐MB‐231	MCF‐10A
**12a**	1.23 ± 0.1	≥ 50
**12g**	10.7 ± 0.9	≥ 50
**17**	12.3 ± 1.1	≥ 50
**Olaparib**	3.45 ± 0.2	≥ 50

a IC_50_ values are expressed in Mean ± SD of three independent replicates. IC_50_ values were calculated by GraphPad Prism software.

### 2.4 PARP‐1 Inhibition

**Figure 5:**
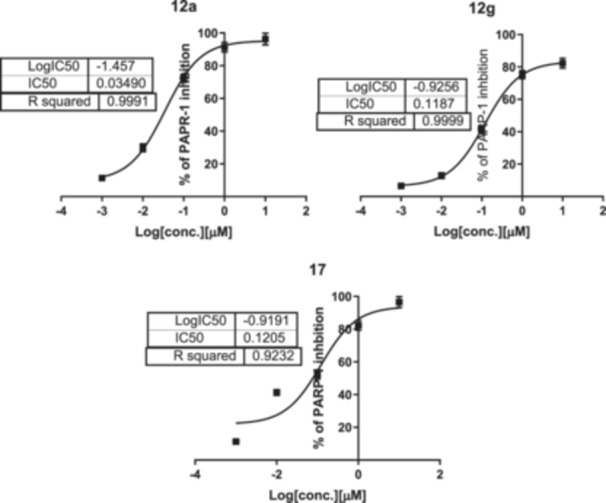
Dose‐response curves of the most active compounds as PARP‐1 inhibitors.

Compounds **12a, 12g**, and **17** were tested for their inhibitory effects against PARP‐1 to determine their molecular targets. As seen in Table 3 and Figure 5, the tested compounds showed promising PARP‐1 kinase inhibition activity; interestingly, compound **12a** exhibited IC_50_ value of 0.034 µM, compared to Olaparib (IC_50_ = 0.012 µM). Compounds **12g** and **17** exhibited promising PARP‐1 inhibition with IC_50_ values of 0.118 and 0.012 µM, respectively.

**Table 3:** IC_50_ values and inhibition percentages of PARP‐1 kinases inhibition of the most cytotoxic compounds.

Compound	PARP‐1
	IC_50_ ± SD (µM)^a^
**12a**	0.034 ± 0.001
**12g**	0.118 ± 0.009
**17**	0.120 ± 0.015
**Olaparib**	0.012 ± 0.001

a “Values are expressed as an average of three independent replicates.” “IC_50_ values were calculated using sigmoidal non‐linear regression curve fit of percentage inhibition against five concentrations of each compound.”

### 2.5 Compound 12a Induced Apoptosis in MDA‐MB‐231 Cancer Cells

**Figure 6:**
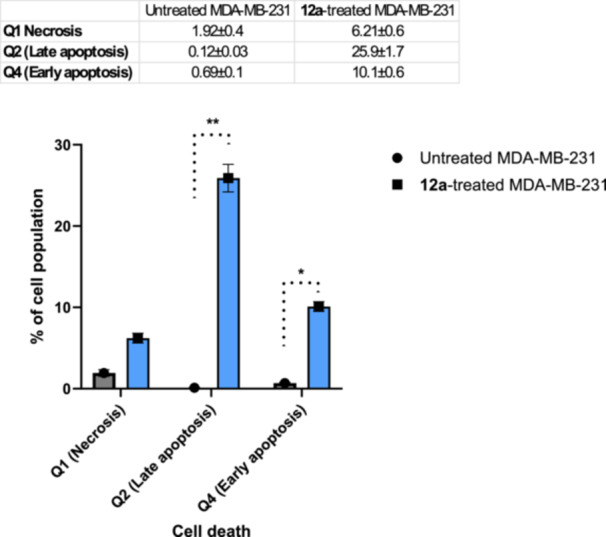
Apoptosis/necrosis assessment in the untreated and **12a**‐treated MDA‐MB‐231 cancer cells at the IC_50_ value for 48h. Q1: necrotic; Q2: late apoptotic and Q4: early apoptotic cell death. *(*p* ≤ 0.05) **(*p* ≤ 0.001) significantly different between treated and untreated using GraphPad prism.

MDA‐MB‐231 cancer cells treated with compound **12a** at the IC_50_ (1.23 μM, 48 h) were then stained by Annexin V‐FITC/PI staining. To compare apoptotic and necrotic cell death of the tested compound **12a** in untreated and treated MDA‐MB‐231 cells, flow cytometric measurement of Annexin V/PI staining was used. Notably, Figure 6 illustrates that compound **12a** markedly enhanced apoptotic cell death in breast cancer cells, increasing total apoptosis to 36.0% (10.1% early apoptosis and 25.9% late apoptosis) compared with the control group, which exhibited only 0.81% total apoptosis (0.12% early and 0.69% late apoptosis) Compound **12a** induced necrotic cell death (6.21%, compared to 1.92% for the control). Therefore, compound **12a** treatment caused more apoptotic than necrotic cell death. For further investigation, MDA‐MB‐231 cancer cells treated with compound **12a** were subjected to DNA flow cytometry to determine when cell proliferation was inhibited.

### 2.6 Compound 12a Arrested the Cell Cycle at the G2/M‐phase in MDA‐MB‐231 Cancer Cells

**Figure 7:**
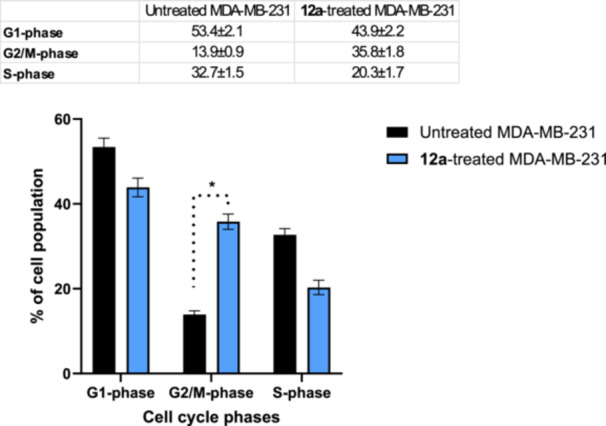
Bar presentation of quantified cell populations in cell cycle phases in the untreated and **12a**‐treated MDA‐MB‐231 cells treated with the IC_50_ value for 48 h. *(*p* ≤ 0.05) significantly different between treated and untreated using GraphPad prism.

As shown in Figure 7, treatment with compound **12a** significantly increased the proportion of cells in the G2/M phase from 13.9% to 35.8%. While it decreased the cell in the S‐phase from 32.7% to 20.3%, and the G1‐phae from 53.4% to 43.9%. Consequently, compound **12a** induced cell cycle arrest at the G2/M‐phase and blocked the progression of MDA‐MB‐231 cancer cells. Our findings of the inhibition of PARP‐1 accompanied by the G2/M arrest in the proliferation of MDA‐MB‐231 cancer cells, agreed with a previous study in which they concluded PARP inhibitors, used either alone or in combination with DNA damage agents, may cause a G2/M mitotic arrest and/or apoptosis in a susceptible genetic context (Madison et al. 2011).

### 2.7 Compound 12a Treatment Affected the Apoptosis‐Related Genes in the MDA‐MB‐231

**Figure 8:**
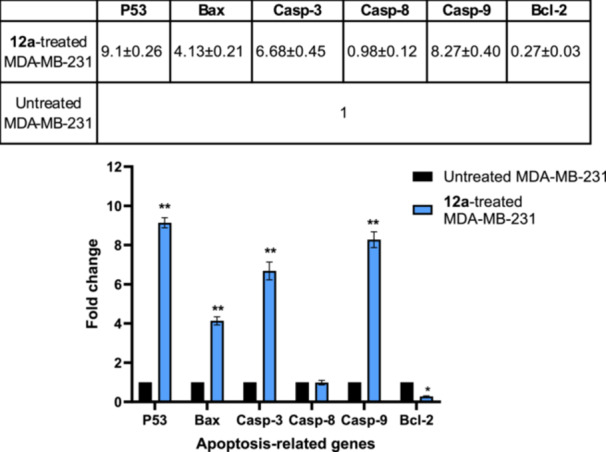
Quantitative RT‐PCR result analysis of the apoptosis‐related genes P53, Bax, caspase‐3, caspase‐8, caspase‐9, and Bcl‐2, respectively, in the untreated and treated MDA‐MB‐231 cells with compound **12a** (IC_50_ = 1.23 μM, 48 h). The housekeeping gene was β‐actin. Fold change between untreated and **12a**‐treated groups was calculated using the equation 2^−^^ΔΔCT^ for differential quantification of gene expression. *(*p* ≤ 0.05) and **(*p* ≤ 0.001) is significantly different between untreated and treated using unpaired *t*‐test in GraphPad Prism.

Compound **12a** demonstrated the highest activity in the series, with an IC_50_ value of 1.23 μM against the MDA‐MB‐231 cancer cell line. Both the intrinsic and extrinsic apoptotic pathways were investigated using gene expression analysis and RT‐PCR to control apoptosis. As shown in Figure 8, compound **12a** treatment induced an increase in the expression of pro‐apoptotic genes, such as P53 (fold change = 9.1), Bax (fold change = 4.13), Caspase‐3 (fold change = 6.68), and Caspase‐9 (fold change = 8.27), while it downregulated the caspase‐8 expression by fold change of 0.98. Furthermore. Compound **12a** simultaneously suppressed the expression of the anti‐apoptotic gene Bcl‐2 by 0.27‐fold. The results illustrated that apoptosis‐induction activity was more intrinsic than extrinsic pathway.

### 2.8 Molecular Modeling

**Figure 9:**
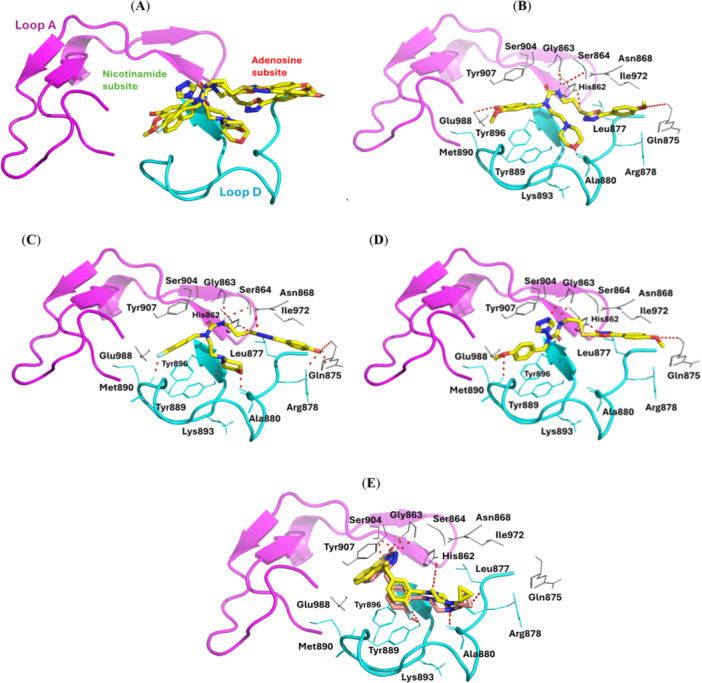
Molecular docking findings for the synthesized compounds shown activity through in vitro PARP‐1's inhibition assay. (A) Overlaid conformation/orientation of the three active PARP‐1 synthesized compound inhibitors shown as yellow sticks at PARP‐1's substrate binding pocket (PDB: 7kk4); (B–D) Predicted binding modes of the active synthesized compounds, **12a**, **12g**, and **17**, respectively; (E) Superimposed co‐crystallized Olaparib (Simone sticks) over its redcoked conformation (yellow sticks) at PARP‐1's catalytic site. Only key surrounding residues within a 4‐Å radius are represented as lines, while polar interactions are illustrated as red‐dashed lines.

Molecular docking simulations of the three top‐active PARP‐1 inhibitors, **12a, 12g**, and **17**, have been performed to explore the molecular aspects of these synthesized compounds towards the cancer‐associated target enzyme (Figure 9). Relative conformation/orientation and residue‐wise interaction patterns for the docked compounds in respect to PPAR1 were highlighted across the molecular docking simulation as compared to PARP‐1 positive control inhibitor, Oalparib (PDB: 7kk4). Findings from this virtual tool have been proposed as beneficial for guiding prospective lead development and optimization processes (Patra 2021; Wu 2023). Simulation runs predicted relevant PARP‐1's pocket accommodation by the three investigated compounds with excellent binding energies at high negative values; –8.18, –9.48, and –6.98 Kcal/mol for **12a**, **12g**, and **17**, respectively. Notably, the three investigated compounds showed relevant anchoring across both the nicotinamide and adenosine subsites of the PARP‐1's catalytic pocket.

As expected, both compounds **12a** and **12g** directed their benzyl urea scaffolds near the conserved Gly863/Ser904 residues, while placing their 1,3,4‐oxadiazole‐based tails at the lining surface of the adenosine subsite. Relevant polar interactions (hydrogen bond distances < 3.10 Å; angles > 127°) have been postulated by the compound's polar decorations towards the pocket lining residues such as His862, Gly863, Ser864, Gln875, Arg878, Ala880, Tyr889, Met890, and/or Glu988. Besides polar contacts, the aromatic rings at compounds **12a** and **12g** predicted sandwiched π‐driven short‐distance contacts (< 5.00 Å) with vicinal non‐polar residues, including Loop D Tyr889/Tyr896 and vicinal Tyr907. Finally, the morpholine arm extended at the space between the Loop A and Loop D towards the solvent side. The latter would postulate the role of the morpholine arm in the disruption of the PARP's less conserved secondary structure, Loop A. It is worth mentioning that compound **12a** showed more extended residue‐wise interaction patterns than **12g** (11 vs. 6 H‐bonds), which translated into greater inhibition of PARP‐1 (IC_50_).

Regarding compound **17**, relevant contacts with PARP‐1's key binding/catalytic residues have been highlighted. The docked compound predicted polar contacts with His862, Gly863, and Met896 residues at the nicotinamide subsites, as well as towards Gln875 at the adenosine subsite. Proximal π‐driven contacts towards Loop D Tyr889/Tyr896 and vicinal Tyr907 residues were also illustrated across the molecular docking simulation. Interestingly, compound **17** depicted differential orientation, particularly for the triazole scaffold, at the PARP‐1's pocket as compared to the earlier morpholine‐based compound analogs. The 1,3,4‐triazole ring showed preferential orientation towards the inner side of the nicotinamide subsite in an opposite orientation to that of the morpholine ring. This may be rationalized by the aromatic nature of the 1,3,4‐triazole ring, which predicts a favored orientation towards the Tyr907 side chain.

Validation of the obtained docked results was performed by redocking the co‐crystallized ligand, Oalparib, using the same docking protocol and algorithm as for the synthesized compounds. Superimposed root mean‐squared deviation (RMSD) between the co‐crystallized Oalparib and its redocked conformation/orientation was at a low value of 1.06 Å. Obtaining an RMSD value less than 2 Å cut‐off generally indicates that the docking protocol/procedure is valid, as it can replicate the co‐crystallized ligand binding while furnishing both the docked binding energies and modes at high predicted biological significance (Kontoyianni et al. 2004; Albuquerque 2020; de Souza 2019; El‐Naggar 2022; Elhady 2021; Maher 2019). Notably, redocked Olaparib replicated the crystallized residue‐wise interaction pattern, successfully translating it into a relevant docking score (–10.78 Kcal/mol) that was higher than any of the docked compounds. The latter showed good agreement with its most potent PARP‐1 inhibition profile, down to low two‐digit nanomolar concentrations, across the enzymology in vitro assay.

### 2.9 Chemistry

All solvents, reagents, and starting materials (4‐(2‐aminoethyl)morpholine, 2‐chloroethyl isocyanate, vanillin, *p*‐hydroxybenzaldehyde, *p*‐flourbenzaldehyde, nicotinic acid) were purchased from ACROS Chemicals Ltd. and Sigma‐Aldrich. Solvent (if necessary) are purified and distilled according to the standard procedures. Thin layer chromatography (TLC) was performed on UV fluorescent Silica gel Merck 60 F254 plates, and the TLC spots were visualized using a UV lamp (254 nm). Purification on column chromatography was performed using packed silica gel (Silica Gel 230–400 mesh) columns. A Bruker (Billerica, Massachusetts, USA) spectrophotometer (400 and 500 MHz) was used to perform the NMR spectra, with TMS serving as an internal standard. For high‐resolution mass spectroscopy, the samples were acquired with the aid of a Bruker Apex IV (7 Tesla) instrument (Bruker) (ionization: ESI (mode: positive), scan modes used: high resolution; full‐scan MS1 (*m*/z 50–950) resolution: 70,000). Purity of the samples is >95% and was confirmed by HRMS less than 5.0 ppm error

#### 2.9.1 General Procedure for the Synthesis of Compounds (**3a**–**d**)

To a stirred solution of aldehyde (1.0 eq.) in isopropanol (20 mL) with few drops of acetic acid, 4‐(2‐aminoethyl)morpholine (1.1 eq.) was added. The resulting reaction mixture was stirred at rt for 1 h. Sodium borohydride (NaBH_4_) (2.0 eq.) was added portionwise at 0°C and left stirring at room temperature for 1 h. After completion of the reaction, solvent was concentrated under reduced pressure to get a crude residue. The residue was basified with saturated solution of NaHCO_3_ and extracted with dichloromethane (2 × 50 mL), dried over MgSO_4_ and concentrated in vacuum to afford pure compounds (without any further purifications).

#### 2.9.2 *N*‐(4‐fluorobenzyl)‐2‐morpholinoethan‐1‐amine (**3a**)

Yield: 84% (yellow oil). TLC analysis (DCM/MeOH: (20:1), Rf value = 0.15). ^1^H NMR (500 MHz, CDCl_3_) δ 7.31–7.27 (m, 2H, Ar‐H), 7.08–7.03 (m, 2H, Ar‐H), 3.82 (s, 2H, ArCH_2_NH), 3.73–3.67 (m, 4H, OCH_2_ (morpholine)), 2.67 (2H, CH_2_NH), 2.56–2.50 (m, 6H, NCH_2_ (morpholine) and CH_2_N). 13C NMR (125 MHz, CDCl_3_) δ 163.12, 161.15, 136.72, 136.70, 130.32, 130.25, 115.47, 115.29, 66.82, 57.20, 53.37, 52.84, 47.16.

#### 2.9.3 *N*‐(4‐methoxybenzyl)‐2‐morpholinoethan‐1‐amine (**3b**)

Yield: 82% (orange oil). TLC analysis (DCM/MeOH: (20:1), Rf value = 0.10). ^1^H NMR (500 MHz, CDCl_3_). δ 6.84–6.77 (m, 2H, Ar‐H), 6.76 (d, J = 8.3 Hz, 1H, Ar‐H), 6.61 (s, 1H, Ar‐H), 3.90 (s, 3H, OCH_3_), 3.85 (s, 2H, ArCH_2_NH), 3.73–3.67 (m, 4H, OCH_2_ (morpholine)), 2.79 (2H, CH_2_NH), 2.67 (2H, CH_2_N), 2.56–2.50 (m, 4H, NCH_2_ (morpholine)). ^13^C NMR (125 MHz, CDCl_3_) δ 147.53, 145.63, 131.72, 121.86, 115.37, 112.31, 66.82, 57.19, 56.05, 53.53, 53.37, 47.16.

#### 2.9.4 *N*‐(4‐chlorobenzyl)‐2‐morpholinoethan‐1‐amine (**3c**)

Yield: 77% (yellow oil). TLC analysis (DCM/MeOH: (20:1), Rf value = 0.2). ^1^H NMR (500 MHz, CDCl_3_) δ 7.35–7.27 (m, 4H, Ar‐H), 3.78 (s, 2H, ArCH_2_NH), 3.72–3.66 (m, 4H, OCH_2_ (morpholine)), 2.78 (t, J = 6.0 Hz, 2H, CH_2_NH), 2.54 (t, J = 6.0 Hz, 2H, CH_2_N), 2.48–2.42 (m, 4H, NCH_2_ (morpholine)). ^13^C NMR (125 MHz, CDCl_3_) δ 138.61, 133.70, 129.80, 128.61, 66.82, 57.20, 53.45, 53.37, 47.17.

#### 2.9.5 2‐Methoxy‐4‐(((2‐morpholinoethyl)amino)methyl)phenol (**3d**)

Yield: 86% (yellow oil). TLC analysis (DCM/MeOH: (20:1), Rf value = 0.2). ^1^H NMR (500 MHz, CDCl_3_) δ 6.86 (1H, Ar‐H), 6.82 (s, 1H, Ar‐H), 6.76 (1H, Ar‐H), 3.88 (s, 3H, OCH_3_), 3.75 (s, 2H, ArCH_2_NH), 3.73‐3.67 (m, 4H, OCH_2_ (morpholine)), 2.78 (2H, CH_2_NH), 2.54 (2H, CH_2_N), 2.52‐2.46 (m, 4H, NCH_2_ (morpholine)). ^13^C NMR (125 MHz, CDCl_3_) δ 158.96, 132.25, 129.30, 113.79, 66.82, 57.20, 55.35, 53.37, 47.16.

##### 2.9.5.1 5‐(3‐Pyridyl)‐1,3,4‐oxadiazole‐2‐thione (**7**)

Compound **7** was prepared according to our modified procedure.

A mixture of nicotinic acid (6.0 g, 0.048 mol), 20 mL of dry methanol, and 4 drops of concentrated sulfuric acid was refluxed for 8 h. The solvent was evaporated under reduced pressure, and the crude product was washed with water (20 mL). A solution of sodium hydrogen carbonate was added to make the solution basic, the mixture was extracted with CH_2_Cl_2_ (3 × 20 mL), and the extract was evaporated under reduced pressure, the produced methyl ester was taken and used directly for the next step.

Hydrazine hydrate (5 mL) was added to a solution of nicotine ester (5.0 g; 0.036 mol) in 25 mL ethanol, and the mixture was refluxed for 6 h. The mixture was cooled and poured onto crushed ice, and the solid product was filtered off, dried, and recrystallized from ethanol.

A solution of nicotinohydrazide (4.0 g: 0.029 mol), potassium hydroxide (1.42 g, 0.025 mol), carbon disulfide (3.0 mL, 0.04 mol), and DMF (15.0 mL) was heated under reflux for 6 h. Solution was then cooled and poured into cooled water; precipitate then acidified with 1.0 mL hydrochloric acid. The resulting precipitate was filtered, collected and dried to give pure *5‐(3‐pyridyl)‐1,3,4‐oxadiazole‐2‐thione*

##### 2.9.5.2 General Procedure for the Synthesis of Compounds (**4a**–**d**)

2‐Chloroethyl isocyanate (12 mmol) was added to a solution of the amines (**a**–**e**) (10 mmol) in ethanol and left stirring at 50°C for 1 h. The completion of reactions was monitored by TLC. Solvent was removed by vacuum and the crude product was sufficiently pure and used directly for the next step.

##### 2.9.5.3 General Procedure for the Synthesis of Compounds (**8a**–**d**)

The obtained chlorocarboxamides **4**(**a**–**d**) (1.0 eq.) and compound **7** (1.1 eq.) were dissolved in acetonitrile (10 mL) and stirred at room temperature for 10 min. Triethylamine (1.0 eq.) was added to the reaction solution and homogenous solution was heated to 50°C for 12 h. The reaction progress was monitored by (TLC). Upon completion, the reaction mixture was concentrated under reduced pressure, and the resulting crude product was purified by silica gel column chromatography using a DCM/methanol (20:1) eluent system.

#### 2.9.6 1‐(4‐Fluorobenzyl)‐1‐(2‐morpholinoethyl)‐3‐(2‐((5‐(pyridin‐3‐yl)‐1,3,4‐oxadiazol‐2‐yl)thio)ethyl) Carboxamide (**8a**)

Yield: 83% (white solid). TLC analysis (DCM/MeOH: (20:1), Rf value = 0.15). ^1^H NMR (500 MHz, CDCl_3_) δ 9.17 (s, 1H, Ar‐H), 8.73 (d, *J* = 4.8 Hz, 1H, Ar‐H), 8.25 (d, *J* = 8.0 Hz, 1H, Ar‐H), 7.70 (br s, 1H, NH), 7.41 (dd, *J* = 8.0, 4.8 Hz, 1H, Ar‐H), 7.18 (m, 2H, Ar‐H), 6.91 (m, 2H, Ar‐H), 4.39 (s, 2H, ArCH_2_N), 3.68‐3.59 (m, 6H, OCH_2_ (morpholine) and CH_2_S), 3.49 (t, *J* = 6.2 Hz, 2H, CH_2_NH), 3.20 (t, *J* = 6.5 Hz, 2H, CH_2_N), 2.42–2.36 (m, 6H, NCH_2_ (morpholine) and CH_2_‐morpholine). ^13^C NMR (126 MHz, CDCl_3_) δ 165.29, 163.72, 159.60, 152.37, 147.60, 133.89, 129.43, 129.37, 123.83, 120.09, 115.51, 115.34, 66.91, 58.95, 53.90, 50.71, 45.14, 40.17, 33.23, 29.67. High‐resolution MS/MS, ESI (+1) calcd for C_23_H_27_FN_6_O_3_S, 486.18494, *m*/*z*: found: 487.19102

#### 2.9.7 1‐(4‐Methoxybenzyl)‐1‐(2‐morpholinoethyl)‐3‐(2‐((5‐(pyridin‐3‐yl)‐1,3,4‐oxadiazol‐2‐yl)thio)ethyl) Carboxamide (**8b**)

Yield: 80% (colorless oil). TLC analysis (DCM/MeOH: (20:1), Rf value = 0.15). ^1^H NMR (500 MHz, CDCl_3_) δ 9.16 (s, 1H, pyridine‐H), 8.72 (d, *J* = 4.8 Hz, 1H, Ar‐H), 8.24 (d, *J* = 8.0 Hz, 1H, Ar‐H), 7.40 (dd, *J* = 8.0, 4.8 Hz, 1H, Ar‐H), 7.11 (d, *J* = 8.5 Hz, 2H, Ar‐H), 6.76 (d, *J* = 8.5 Hz, 2H, Ar‐H), 4.36 (s, 2H, ArCH_2_N), 3.71 (s, 3H, OCH_3_), 3.68‐3.57 (m, 6H, OCH_2_ (morpholine) and CH_2_S), 3.47 (t, *J* = 6.2 Hz, 2H, CH_2_NH), 3.20 (t, *J* = 6.5 Hz, 2H, CH_2_N), 2.43‐2.33 (m, 6H, NCH_2_ (morpholine) and CH_2_‐morpholine). ^13^C NMR (126 MHz, CDCl_3_) δ 165.31, 163.68, 159.58, 158.90, 152.36, 147.62, 133.87, 130.24, 129.01, 120.09, 113.97, 113.93, 67.02, 66.97, 58.94, 55.25, 53.90, 53.84, 50.77, 49.80, 45.06, 40.14, 38.44, 33.23. High‐resolution MS/MS, ESI (+ 1) calcd for C_24_H_30_N_6_O_4_S, 498.20492, *m*/*z*: found: 499.21481

#### 2.9.8 1‐(4‐Chlorobenzyl)‐1‐(2‐morpholinoethyl)‐3‐(2‐((5‐(pyridin‐3‐yl)‐1,3,4‐oxadiazol‐2‐yl)thio)ethyl) Carboxamide (**8c**)

Yield: 75% (yellow oil). TLC analysis (DCM/MeOH: (20:1), Rf value = 0.25). ^1^H NMR (500 MHz, CDCl_3_)) δ 9.19 (s, 1H, (pyridine‐H)), 8.73 (d, *J* = 4.8 Hz, 1H, Ar‐H), 8.26 (d, *J* = 8.0 Hz, 1H, Ar‐H), 7.40 (dd, *J* = 8.0, 4.8 Hz, 1H, Ar‐H), 7.23 (d, *J* = 8.4 Hz, 2H, Ar‐H), 7.13 (d, *J* = 8.4 Hz, 2H, Ar‐H), 4.40 (s, 2H, ArCH_2_N), 3.66‐3.64 (m, 6H, OCH_2_ (morpholine) and CH_2_S), 3.50 (t, *J* = 6.2 Hz, 2H, CH_2_NH), 3.18 (t, J = 6.5 Hz, 2H, CH_2_N), 2.47–2.40 (m, 6H, NCH_2_ (morpholine) and CH_2_‐morpholine). ^13^C NMR (126 MHz, CDCl_3_) δ 165.29, 163.75, 159.64, 152.42, 147.65, 133.87, 133.11, 129.14, 128.72, 123.88, 123.81, 123.61, 120.06, 66.95, 59.09 53.82, 50.85, 45.30, 40.20, 33.23, 29.69. High‐resolution MS/MS, ESI (+1) calcd for C_23_H_27_ClN_6_O_3_S, 502.15539, *m*/*z*: found: 503.16343

#### 2.9.9 1‐(4‐Hydroxy‐3‐methoxybenzyl)‐1‐(2‐morpholinoethyl)‐3‐(2‐((5‐(pyridin‐3‐yl)‐1,3,4‐oxadiazol‐2‐yl)thio)ethyl) Carboxamide (**8d**)

Yield: 75% (yellow oil). TLC analysis (DCM/MeOH: (20:1), Rf value = 0.25). ^1^H NMR (500 MHz, CDCl_3_) δ 9.15 (s, 1H, (pyridine‐H)), 8.71 (d, *J* = 4.8 Hz, 1H, Ar‐H), 8.23 (d, *J* = 8.0 Hz, 1H, Ar‐H), 7.41 (dd, *J* = 8.0, 4.8 Hz, 1H, Ar‐H), 6.82 (d, *J* = 8.1 Hz, 1H, Ar‐H), 6.74 (s, 1H, Ar‐H), 6.63 (d, *J* = 8.1 Hz, 1H, Ar‐H), 5.75 (br s, 1H, OH), 4.34 (s, 2H, ArCH_2_N), 3.78 (s, 3H, OCH_3_), 3.68‐3.60 (m, 6H, OCH_2_ (morpholine) and CH_2_S), 3.47 (t, *J* = 6.2 Hz, 2H, CH_2_NH), 3.26 (t, *J* = 6.5 Hz, 2H, CH_2_N), 2.47‐2.38 (m, 6H, NCH_2_ (morpholine) and CH_2_‐morpholine). ^13^C NMR (126 MHz, CDCl_3_) δ 165.31, 163.68, 159.58, 158.90, 152.36, 147.62, 133.87, 130.24, 129.01, 123.80, 120.09, 113.97, 113.93, 67.02, 66.97, 58.94, 55.25, 53.90, 53.84, 50.77, 45.06, 40.14, 33.23. High‐resolution MS/MS, ESI (+1) calcd for C_24_H_30_N_6_O_5_S, 514.19984, *m*/*z*: found: 515.20541.

#### 2.9.10 General Procedure for Compounds **11a** and **11b**

A mixture of acid (0.05 mol), 30 mL of dry methanol, and 1 ml of concentrated sulfuric acid was refluxed for 8 h. The solvent was evaporated under reduced pressure, and the crude product was washed with water (20 mL). A solution of sodium hydrogen carbonate was added to make the solution basic, the mixture was extracted with CH_2_Cl_2_ (3 × 20 mL), and the extract was evaporated under reduced pressure. The methyl ester of the corresponding acid was used directly for the next step. Hydrazine hydrate (3.5 mL; 4.0 eq.) was added to a solution of methyl ester (0.03 mol) in 20 mL ethanol, and the mixture was refluxed for 12 h. The mixture was cooled and poured onto crushed ice, and the solid product was filtered off, dried, and recrystallized from ethanol.

A solution of acid hydrazide (0.03 mol), potassium hydroxide (0.03 mol), carbon disulfide (0.05 mol), and ethanol (15.0 mL) were heated under reflux for 8 h. Solution was then cooled and poured into cooled water; precipitate then acidified with 1.0 mL conc. HCl. The resulting precipitate was filtered, collected and dried to give pure compounds

##### 2.9.10.1 5‐(4‐Hydroxyphenyl)‐1,3,4‐oxadiazole‐2‐thione **11a**

Yield: 70% (yellow solid). Lit (Dash 2011) (300 MHz, DMSO‐*d*_6_) *δ*: 10.16 (br, 1*H*, SH), 9.54 (s, 1*H*, OH), 7.71 (d, *J* = 6.8 Hz, 2H, H‐2ʹ,6ʹ Ar), 6.81 (d, *J* = 6.8 Hz, 2*H*, H‐3ʹ,5ʹ Ar) ppm

##### 2.9.10.2 5‐(4‐Methoxyphenyl)‐1,3,4‐oxadiazole‐2‐thione **11b**

Yield: 78% (yellow solid). The spectroscopic data are in accordance with Lit (Yang 2024).

#### 2.9.11 General Procedure for the Synthesis of Compounds (**12a**–**g**)

The synthesis of compounds **12(a**–**g)** was carried out following the same procedure previously described for the preparation of compounds **8(a**–**d)**

#### 2.9.12 1‐(4‐Fluorobenzyl)‐3‐(2‐((5‐(4‐hydroxyphenyl)‐1,3,4‐oxadiazol‐2‐yl)thio)ethyl)‐1‐(2‐morpholinoethyl) Carboxamide **12a**

Yield: 70% (white solid). TLC analysis (DCM/MeOH: (20:1), Rf value = 0.15). ^1^H NMR (500 MHz, CDCl_3_) δ 7.92 (d, J = 8.8 Hz, 2H, Ar‐H), 7.72 (dd, J = 8.5, 5.5 Hz, 2H, Ar‐H), 7.15 (m, 2H, Ar‐H), 6.90 (d, *J* = 8.8 Hz, 2H, Ar‐H), 4.40 (s, 2H, Ar‐CH_2_N), 3.69‐3.55 (m, 6H, OCH_2_ (morpholine) and CH_2_S), 3.43 (m, 2H, ─CH_2_─NH), 3.24 (t, *J* = 6.2 Hz, 2H, CH_2_NH), 2.43 (m, 4H, N(CH_2_)_2_), 1.98 (t, *J* = 6.2 Hz, 2H, ─CH_2_–(morpholine)). ^13^C NMR (126 MHz, CDCl_3_) δ 166.16, 163.26, 161.12, 160.68, 159.49, 129.29, 128.57, 127.99, 122.28, 115.39, 114.54, 66.55, 58.25, 53.61, 50.67, 44.61, 40.38, 33.02, 29.67. High‐resolution MS/MS, ESI (+1) calcd for C_24_H_28_FN_5_O_4_S, 501.18460, *m*/*z*: found: 502.19171

#### 2.9.13 1‐(4‐Hydroxy‐3‐methoxybenzyl)‐3‐(2‐((5‐(4‐hydroxyphenyl)‐1,3,4‐oxadiazol‐2‐yl)thio)ethyl)‐1‐(2‐morpholinoethyl) Carboxamide **12b**

Yield: 73% (white solid). TLC analysis (DCM/MeOH: (20:1), Rf value = 0.20). ^1^H NMR (500 MHz, CDCl_3_) δ 7.73 (d, *J* = 8.7 Hz, 2H, Ar‐H), 6.95 (d, *J* = 8.1 Hz, 1H, Ar‐H), 6.88 (d, *J* = 8.7 Hz, 2H, Ar‐H), 6.82 (d, *J* = 1.9 Hz, 1H, Ar‐H), 6.62 (dd, *J* = 8.1, 1.9 Hz, 1H, Ar‐H), 4.31 (s, 2H, Ar‐CH_2_N), 3.78 (s, 3H, ─OCH_3_), 3.69–3.56 (m, 6H, O(CH_2_)_2_ (morpholine)) and CH_2_S), 3.44 (m, 2H, ─CH_2_NH), 3.18 (t, J = 6.2 Hz, 2H, ─CH_2_N), 2.38 (m, 4H, N(CH_2_)_2_ (morpholine)), 2.01 (t, *J* = 6.2 Hz, 2H, ─CH_2_‐morpholine). ^13^C NMR (126 MHz, CDCl_3_) δ 166.07, 163.18, 160.21, 146.91, 145.08, 128.57, 122.99, 116.20, 116.16, 114.91, 114.26, 110.43, 66.94, 55.98, 55.95, 53.85, 53.83, 51.32, 44.94, 43.73, 40.44, 33.24, 29.70. High‐resolution MS/MS, ESI (+1) calcd for C_25_H_31_N_5_O_6_S, 529.19950, *m*/*z*: found: 530.24317

#### 2.9.14 1‐(4‐Fluorobenzyl)‐3‐(2‐((5‐(4‐methoxyphenyl)‐1,3,4‐oxadiazol‐2‐yl)thio)ethyl)‐1‐(2‐morpholinoethyl) Carboxamide **12c**

Yield: 83% (colorless oil). TLC analysis (DCM/MeOH: (20:1), Rf value = 0.25). ^1^H NMR (500 MHz, CDCl_3_) δ 7.87 (d, *J* = 8.8 Hz, 2H, Ar‐H), 7.23 (dd, *J* = 8.5, 5.5 Hz, 2H, Ar‐H), 7.15 (m, 2H, Ar‐H), 6.96 (d, *J* = 8.8 Hz, 2H, Ar‐H), 6.91 (m, 2H, Ar‐H), 4.38 (s, 2H, Ar‐CH_2_N), 3.83 (s, 3H, ─OCH_3_), 3.61 (m, 6H, O(CH_2_)_2_ (morpholine) and CH_2_S), 3.43 (m, 2H, ─CH_2_NH), 3.17 (t, *J* = 6.2 Hz, 2H, ─CH_2_N), 2.45 (m, 4H, N(CH_2_)_2_ (morpholine)), 2.36 (t, *J* = 6.2 Hz, 2H, ─CH_2_‐morpholine). ^13^C NMR (126 MHz, CDCl_3_) δ 165.84, 163.54, 163.07, 162.36, 161.12, 159.57, 129.40, 129.34, 128.46, 115.99, 115.49, 115.32, 114.53, 66.92, 58.95, 55.48, 53.91, 50.69, 45.13, 40.37, 33.21, 29.70. High‐resolution MS/MS, ESI (+1) calcd for C_25_H_30_FN_5_O_4_S, 515.20025, *m*/*z*: found: 516.20684

#### 2.9.15 1‐(4‐Chlorobenzyl)‐3‐(2‐((5‐(4‐methoxyphenyl)‐1,3,4‐oxadiazol‐2‐yl)thio)ethyl)‐1‐(2‐morpholinoethyl) Carboxamide **12d**

Yield: 70% (yellow oil). TLC analysis (DCM/MeOH: (20:1), Rf value = 0.20). ^1^H NMR (500 MHz, CDCl_3_) δ 7.81 (d, *J* = 8.8 Hz, 2H, Ar‐H), 7.18 (d, *J* = 8.4 Hz, 2H, Ar‐H), 7.14 (d, *J* = 8.4 Hz, 2H, Ar‐H), 6.89 (d, *J* = 8.8 Hz, 2H, Ar‐H), 3.75 (s, 3H, ─OCH_3_), 3.66 (s, 2H, Ar‐CH_2_N), 3.58 (m, 6H, O(CH_2_)_2_ (morpholine) and CH_2_S), 3.38 (m, 2H, ─CH_2_NH), 2.59 (m, 4H, N(CH_2_)_2_ (morpholine)), 2.39 (t, *J* = 6.2 Hz, 2H, ─CH_2_N), 2.30 (t, *J* = 6.2 Hz, 2H, ─CH_2_‐morpholine). ^13^C NMR (126 MHz, CDCl_3_) δ 162.73, 160.90, 140.37, 138.64, 135.75, 132.58, 129.71, 129.21, 128.39, 128.02, 115.37, 114.49, 67.04, 57.97, 54.03, 53.01, 44.90, 29.31. High‐resolution MS/MS, ESI (+1) calcd for C_25_H_30_ClN_5_O_4_S, 531.17070, m/z: found: 532.17798

#### 2.9.16 3‐(2‐((5‐(4‐Methoxyphenyl)‐1,3,4‐oxadiazol‐2‐yl)thio)ethyl)‐1‐(2‐morpholinoethyl)‐1‐(4‐nitrobenzyl) Carboxamide **12e**

Yield: 76% (yellow oil). TLC analysis (DCM/MeOH: (20:1), Rf value = 0.25). ^1^H NMR (500 MHz, CDCl_3_) δ 8.07 (d, *J* = 8.5 Hz, 2H, Ar‐H), 7.87 (d, *J* = 8.8 Hz, 2H, Ar‐H), 7.37 (d, *J* = 8.5 Hz, 2H, Ar‐H), 6.94 (d, *J* = 8.8 Hz, 2H, Ar‐H), 4.54 (s, 2H, Ar‐CH_2_‐N), 3.83 (s, 3H, ─OCH_3_), 3.71‐3.58 (m, 6H, O(CH_2_)_2_ (morpholine), CH_2_S─), 3.45 (m, 2H, CH_2_‐NH─), 3.22 (t, *J* = 6.2 Hz, 2H, CH_2_‐N(CO)), 2.51 (m, 4H, N(CH_2_)_2_ (morpholine)), 2.03 (t, *J* = 6.2 Hz, 2H, ─CH_2_‐morpholine). ^13^C NMR (126 MHz, CDCl_3_) δ 165.90, 163.61, 162.44, 159.58, 159.44, 147.21, 146.27, 139.29, 128.44, 128.24, 123.80, 115.85, 114.57, 66.77, 55.57, 53.83, 51.08, 49.49, 45.40, 40.63, 38.63, 33.13, 31.92, 29.69. High‐resolution MS/MS, ESI (+1) calcd for C_25_H_30_N_6_O_6_S, 542.19475, *m*/*z*: found: 543.20203

#### 2.9.17 1‐(4‐Methoxybenzyl)‐3‐(2‐((5‐(4‐methoxyphenyl)‐1,3,4‐oxadiazol‐2‐yl)thio)ethyl)‐1‐(2‐morpholinoethyl) Carboxamide **12f**

Yield: 80% (colorless oily). TLC analysis (DCM/MeOH: (20:1), Rf value = 0.30). ^1^H NMR (500 MHz, DMSO): δ 7.88 (d, *J* = 8.8 Hz, 2H, Ar‐H), 7.07 (d, *J* = 8.5 Hz, 2H, Ar‐H), 6.99 (d, *J* = 8.8 Hz, 2H, Ar‐H), 6.82 (d, *J* = 8.5 Hz, 2H, Ar‐H), 4.30 (s, 2H, Ar‐CH_2_‐N), 3.80 (s, 3H, ─OCH_3_), 3.78 (s, 3H, ─OCH_3_), 3.66–3.64 (m, 6H, O(CH_2_)_2_ (morpholine), ─CH_2_S), 3.50 (m, 2H, ─CH_2_‐NH), 3.44 (m, 2H, ─CH_2_‐N(CO)), 3.41 (m, 4H, N(CH_2_)_2_ (morpholine)), 3.36 (t, *J* = 6.2 Hz, 2H, ─CH_2_‐morpholine). ^13^C NMR (126 MHz, DMSO): δ 165.42, 163.54, 162.48, 158.74, 158.19, 129.08, 128.72, 115.92, 115.33, 114.20, 56.00, 55.49, 49.37, 33.17. High‐resolution MS/MS, ESI (+1) calcd for C_26_H_33_N_5_O_5_S, 527.22024, *m*/*z*: found:

#### 2.9.18 1‐(4‐Hydroxy‐3‐methoxybenzyl)‐3‐(2‐((5‐(4‐methoxyphenyl)‐1,3,4‐oxadiazol‐2‐yl)thio)ethyl)‐1‐(2‐morpholinoethyl) Carboxamide **12g**

Yield: 78% (white solid). TLC analysis (DCM/MeOH: (20:1), Rf value = 0.30). ^1^H NMR (500 MHz, CDCl_3_) δ 7.54 (1H, s), 7.40 (1H, dd, *J* = 8.0, 4.9 Hz), 7.11 (2H, d, *J* = 8.0 Hz), 6.78 (1H, s), 6.78–6.71 (1H, m), 4.35 (1H, s), 3.71 (3H, d, *J* = 6.4 Hz), 3.71–3.55 (5H, m), 3.47 (1H, t, *J* = 6.3 Hz), 3.20 (2H, dt, *J* = 10.5, 5.0 Hz), 2.45–2.38 (2H, m), 2.34 (2H, t, *J* = 5.1 Hz). ^13^C NMR (126 MHz, CDCl_3_) δ (126 MHz, CDCl_3_): 165.31, 163.68, 159.58, 158.90, 152.36, 147.62, 147.45, 133.87, 133.53, 130.24, 129.01, 123.80, 120.09, 113.97, 113.93, 66.97, 55.25, 53.90, 33.23. High‐resolution MS/MS, ESI (+1) calcd for C_26_H_33_N_5_O_6_S, 543.21515, *m*/*z*: found: 544.25882.

##### 2.9.18.1 4‐Amino‐4H‐1,2,4‐triazole **14**

4‐Amino‐4*H*‐1,2,4‐triazole was prepared according to literature procedure (Sanz 2002).

##### 2.9.18.2 *N*‐(4‐Methoxybenzyl)‐4H‐1,2,4‐triazol‐4‐amine **15**

To a stirred solution of *p*‐anisaldehyde (1.0 eq.) with few drops of acetic acid, 4‐amino‐*4H*‐1,2,4‐triazole **14** (1.1 eq.) was added. The reaction was proceeded neat without solvent for 1 h. The Schiff base formed was dissolved in methanol (10 mL), sodium borohydride (NaBH_4_) (2.0 eq.) was subsequently added portionwise at r.t. and left stirring at room temperature for 1 h. After completion of the reaction, solvent was concentrated under reduced pressure to get a crude residue. The residue was basified with saturated solution of NaHCO_3_ solution and extracted with dichloromethane (2 × 50 mL), dried over MgSO_4_ and concentrated in vacuum to afford pure compound. ^1^H NMR (500 MHz, CDCl_3_) δ 8.03–8.01 (1H, triazole‐H), 7.22–7.19 (d, *J* = 8.5 Hz, 2H, Ar‐H), 6.83–6.79 (d, J = 8.5 Hz, 2H, Ar‐H), 6.17 (br s, 1H, triazole/NH‐related proton), 4.13–4.12 (2H, ArCH_2_NH), 3.77–3.76 (s, 3H, OCH_3_). ^13^C NMR (126 MHz, CDCl_3_) δ (126 MHz, CDCl_3_). ^13^C NMR (CDCl_3_) δ 159.6 (Ar‐C‐O), 143.1, 142.1 (triazole C), 130.2, 130.0 (Ar‐CH), 127.7 (Ar‐CH), 114.3, 114.2, 113.9 (Ar‐CH), 57.3 (Ar‐CH_2_‐NH), 55.3 (OCH_3_) (see Files S41–S42).

#### 2.9.19 1‐(4‐Methoxybenzyl)‐3‐(2‐((5‐(4‐methoxyphenyl)‐1,3,4‐oxadiazol‐2‐yl)thio)ethyl)‐1‐(4H‐1,2,4‐triazol‐4‐yl) Carboxamide **17**

2‐chloroethyl isocyanate (12 mmol) was added to a solution of the diamine **15** (10 mmol) in CHCl_3_ (10 mL) and left stirring at 50°C for 1 h. The completion of reactions was monitored by TLC. Solvent was removed by vacuum and the crude product **16** was used directly for the next step. The chlorocarboxamides (1.0 eq.) and compound **9b** (1.1 eq.) were dissolved in acetonitrile (10 mL) and stirred at room temperature for 10 min. Triethylamine (1.0 eq.) was added to the reaction solution and homogenous solution was heated to 50°C for 12 h. The reaction progress was monitored by (TLC). Upon completion, the reaction mixture was concentrated under reduced pressure, and the resulting crude product was purified by silica gel column chromatography using a DCM/methanol (20:1) eluent system.

Yield: 78% (white solid). TLC analysis (DCM/MeOH: (10:1), Rf value = 0.35). ^1^H NMR (500 MHz, DMSO) δ 8.37 (s, 2H, triazole‐H), 7.92 (d, J = 8.8 Hz, 2H, Ar‐H), 7.15 (d, *J* = 8.5 Hz, 2H, Ar‐H), 7.04 (d, *J* = 8.8 Hz, 2H, Ar‐H), 6.83 (d, *J* = 8.5 Hz, 2H, Ar‐H), 4.74 (s, 2H, ArCH_2_N), 3.83 (s, 3H, OCH_3_), 3.70 (s, 3H, OCH_3_), 3.63‐3.47 (m, 6H, CH_2_CH_2_S and CH_2_NH). ^13^C NMR (126 MHz, DMSO‐d6) δ 163.49, 162.51, 159.43, 156.34, 143.97, 130.49, 129.02, 128.74, 127.64, 127.44, 115.84, 115.37, 115.12, 114.41, 56.01, 55.87, 55.48, 53.94, 32.33. High‐resolution MS/MS, ESI (+1) calcd for C_22_H_23_N_7_O_4_S, 481.1532, *m*/*z*: found: 482.1660

### 2.10 Experimental

#### 2.10.1 Cytotoxicity of tested compounds Using MTT Assay

The percentage of cell growth inhibition at the single dose [10 µM] and cytotoxicity (IC_50_) were determined using the MTT assay (Mosmann 1983) against MDA‐MB‐231, OVCAR‐3 cell lines. This was done at the beginning of the study to determine the single dose % inhibition at 10 µM for the compounds, selecting the most effective ones as anticancer agents. Then, the cytotoxicity of the top cytotoxic compounds against the malignant cell line was compared with that against the normal cell line (MCF‐10A). Cells were treated for 48 h with different serial dilutions (100 to 0.75 µM) of the selected compounds. The optical density (O.D.) was measured spectrophotometrically at 570 nm using an ELISA microplate reader (Sunrise TM, TECAN, Germany). The mean values were estimated as the percentage of cell viability as follows:

$$\%\;\mathrm{cell}\;\mathrm{viability}=\frac{O.D\;(\mathrm{treated}\;\mathrm{cells})}{O.D\;(\mathrm{control}\;\mathrm{cells})}\times 100$$

The IC_50_ value of each drug was calculated using dose‐response curve‐fitting models using GraphPad prism.

#### 2.10.2 PARP‐1 Enzymatic Inhibition Assay

To assess the inhibitory potency of compound **12a** at five serial concentrations of 0.001, 0.01, 0.1, 1, and 10 µM against the PARP‐1 colorimetric assay kit (Bioscience, Cat No. #80580, CA, USA) were used. The following equation was used to calculate the percentage of autophosphorylation inhibition by drugs; Percentage inhibition = $100-\left[\frac{\mathrm{Control}}{\mathrm{Treated}}-\mathrm{Control}\right]$ (Alshams 2026; Nafie 2025; Youssef 2022)

#### 2.10.3 Annexin V/PI Staining

3–10^5^ of MDA‐MB‐231 cells were added to 6‐well culture plates, which were then incubated overnight. Following that, cells were treated with compound **12a** at its IC_50_ for 48 h. Following that, PBS was rinsed with ice‐cold water, and media supernatants were collected. The cells were then treated with “Annexin V‐FITC solution (1:100) and propidium iodide (PI)” at a concentration of 10 g/mL for 30 min in the dark after being suspended in 100 L of Annexin binding buffer solution, which is composed of 25 mM CaCl_2_, 1.4 M NaCl, and 0.1 M Hepes/NaOH, pH 7.4. Then, labeled cells were collected using the Cytoflex FACS system. The data were assessed using the cytExpert program (Vermes 1995).

#### 2.10.4 Gene Expression Analysis Using the RT‐PCR

The apoptotic pathway of compound **12a** against MDA‐MB‐231 cells, gene expression of P53, Bax, Caspases‐3,8,9 pro‐apoptotic genes, and Bcl‐2 as the anti‐apoptotic gene. MDA‐MB‐231 cells were treated with compound **12a** at its IC_50_ value for 48 h. Then, a routine RT‐PCR experiment was run, and the data were reported as cycle thresholds (Ct) and Ct ratios to allow comparison of gene expression levels with those of the housekeeping gene, β‐actin (Elmore 2007; Vousden and Lane 2007; Fulda and Debatin 2006).

#### 2.10.5 Molecular Modeling

The top‐active PARP‐1 inhibitors, **12a**, **12g**, and **17**, were 3D‐constructed, and the PARP‐1 protein (PDB: 7KK4) was prepared. The docking protocol was performed using AutoDock Vina V.1.2.0 (Scripps Research, La Jolla, CA, United States) (Eberhardt 2021). The binding site was defined as the region that co‐crystallized with ligands (benzopyran derivative or celecoxib) and was subsequently refined to include key binding residues for small‐molecule ligands (Wang 2010). Docking was performed using Vina Forcefield and Lamarckian_Genetics, with the biological target center as the docking box center (Eberhardt 2021). Global search exhaustiveness was set to 100 Kcal/mol, and the maximum energy difference for poses was set to 3 Kcal/mol (Xue 2022). Visualizing poses and compound/PARP‐1 binding interactions were performed via PyMol V3.1.3.

## 3 Conclusion

Several new morpholine derivatives tethered to 1,3,4‐oxadiazole rings have been synthesized and screened for anticancer activity. Notably, **12a** showed potent cytotoxicity against MDA‐MB‐231 cells, with an IC_50_ of 1.23 ± 0.1 µM, compared to Olaparib, which had an IC_50_ of 10.3 ± 0.8 µM. Compound **12a** also exhibited potent PARP‐1 inhibitory activity, with an IC_50_ value 0.034 µM, compared to Olaparib (IC_50_ = 0.012 µM). Apoptosis‐induction study revealed that **12a** increased total apoptotic breast cancer by 36.0% compared to control 0.81%, arresting the cell cycle at the G2/M phase at the expenses of S‐phase.

## Conflicts of Interest

The authors declare no conflicts of interest.

## Supporting information


Supporting File

## Data Availability

The data that support the findings of this study are available from the corresponding author upon reasonable request.
