# Supplementary material for: Structure‐Based Design, Synthesis, and Biological Evaluation of Oxadiazole–Morpholine Hybrids as Potent PARP‐1 Inhibitors Inducing Apoptosis in Breast Cancer Cells
Source: Drug Dev Res. 2026 Jul 8;87(5):e70346. doi: 10.1002/ddr.70346 (PMC13344874; doi:10.1002/ddr.70346)
Supplement: Supplementary file 1 — Supporting File [file DDR-87-e70346-s001.pdf]

Supplementary Material

Structure-Based Design, Synthesis, and Biological Evaluation of Oxadiazole–Morpholine Hybrids as Potent PARP-1 Inhibitors Inducing Apoptosis in Breast Cancer Cells

Nader R. Albujuq[1*], Khaled M. Darwish[2,3], Sherif Ashraf Fahmy[4*], Buthaina Hussein[5], Ghada G. Kayed[6], Musa A. Said[7], Mohamed S. Nafie[8,9,10*]

[1]Department of Chemistry, School of Science, The University of Jordan, Amman 11942, Jordan.

[2]Department of Medicinal Chemistry, Faculty of Pharmacy, Galala University, New Galala (P.O. 43713), Egypt.

[3]Medicinal Chemistry Department, Faculty of Pharmacy, Suez Canal University, Ismailia (P.O. 41522), Egypt.

[4] Department of Pharmacy, Institute of Pharmaceutics and Biopharmaceutics, Marburg University, Robert-Koch-Str. 4, 35037, Marburg, Germany.

[5]Department of Pharmacy, Faculty of Pharmacy, Al-Zaytoonah University of Jordan, Amman 11733, Jordan.

[6]Faculty of science, Zarqa University, Zarqa, 13110, Jordan.

[7]Department of Chemistry, Faculty of Science, Islamic University of Madinah, Madinah 42351, Saudi Arabia

[8]Department of Chemistry, College of Sciences, University of Sharjah, Sharjah (P.O. 27272), United Arab Emirates (UAE)

[9] Bioinformatics and Functional Genomics Research Group, Research Institute of Sciences and Engineering (RISE), University of Sharjah, Sharjah 27272, United Arab Emirates

[10]Chemistry Department, Faculty of Science, Suez Canal University, Ismailia, (P.O. 41522), Egypt.

***Correspondence**: **Nader R. Albujuq**: n.albujuq@ju.edu.jo / **Sherif Ashraf Fahmy, Emails: sherif.fahmy@pharmazie.uni-marburg.de/ Mohamed S. Nafie**: mohamed.elsayed@sharjah.ac.ae

Table of contents.	Page
**Figure 1**. 1H-NMR for compound **8a**	**S3**
**Figure 2**. 1H-NMR for compound **8a**	**S5**
**Figure 3**. Mass spectroscopy of high resolution for compound **8a**	**S6**
**Figure 4**. Mass spectroscopy of high resolution for compound **8a**	**S7**
**Figure 5**. 1H-NMR for compound **8b**	**S8**
**Figure 6**. 13C-NMR for compound **8b**	**S9**
**Figure 7**. 1H-NMR for compound **8c**	**S10**
**Figure 8.** 13C-NMR for compound **8c**	**S11**
**Figure 9**. Mass spectroscopy of high resolution for compound **8c**	**S12**
**Figure 10**. Mass spectroscopy of high resolution for compound **8c**	**S13**
**Figure 11**. 1H-NMR for compound **8d**	**S14**
**Figure 12**. 13C-NMR for compound **8d**	**S15**
**Figure 13**. Mass spectroscopy of high resolution for compound **8d**	**S16**
**Figure 14**. Mass spectroscopy of high resolution for compound **8d**	**S17**
**Figure 15**. 1H-NMR for compound **12a**	**S18**
**Figure 16**. 13C-NMR for compound **12a**	**S19**
**Figure 17**. Mass spectroscopy of high resolution for compound **12a**	**S20**
**Figure 18**. Mass spectroscopy of high resolution for compound **12a**	**S21**
**Figure 19**. 1H-NMR for compound **12b**	**S22**
**Figure 20**. 13C-NMR for compound **12b**	**S23**
**Figure 21**. Mass spectroscopy of high resolution for compound **12b**	**S24**
**Figure 22**. Mass spectroscopy of high resolution for compound **12b**	**S25**
**Figure 23**. 1H-NMR for compound **12c**	**S26**
**Figure 24**. 13C-NMR for compound **12c**	**S27**
**Figure 25**. Mass spectroscopy of high resolution for compound **12c**	**S28**

**Figure 26**. 1H-NMR for compound **12d**	**S29**
**Figure 27**. 13C-NMR for compound **12d**	**S30**
**Figure 28**. Mass spectroscopy of high resolution for compound **12d**	**S31**
**Figure 29**. 1H-NMR for compound **12e**	**S32**
**Figure 30**. 13C-NMR for compound **12e**	**S33**
**Figure 31**. Mass spectroscopy of high resolution for compound **12e**	**S34**
**Figure 32**. 1H-NMR for compound **12f**	**S35**
**Figure 33**. 13C-NMR for compound **12f**	**S36**
**Figure. 34**. Mass spectroscopy of high resolution for compound **12f**	**S37**
**Figure 35**. Mass spectroscopy of high resolution for compound **12g**	**S38**
**Figure 36**. 1H-NMR for compound **17**	**S39**
**Figure 37**. 13C-NMR for compound **17**	**S40**
**Figure 38**. 1H-NMR for compound **15**	**S41**
**Figure 39**. 13C-NMR for compound **15**	**S42**
**Figure 40:** Representative histogram of Annexin V/PI staining in the untreated and **12a**-treated MDA-MB-231 cancer cells	**S43**
**Figure 41:** Representative histogram of cell cycle analysis in the untreated and **12a**-treated MDA-MB-231 cancer cells	**S43**

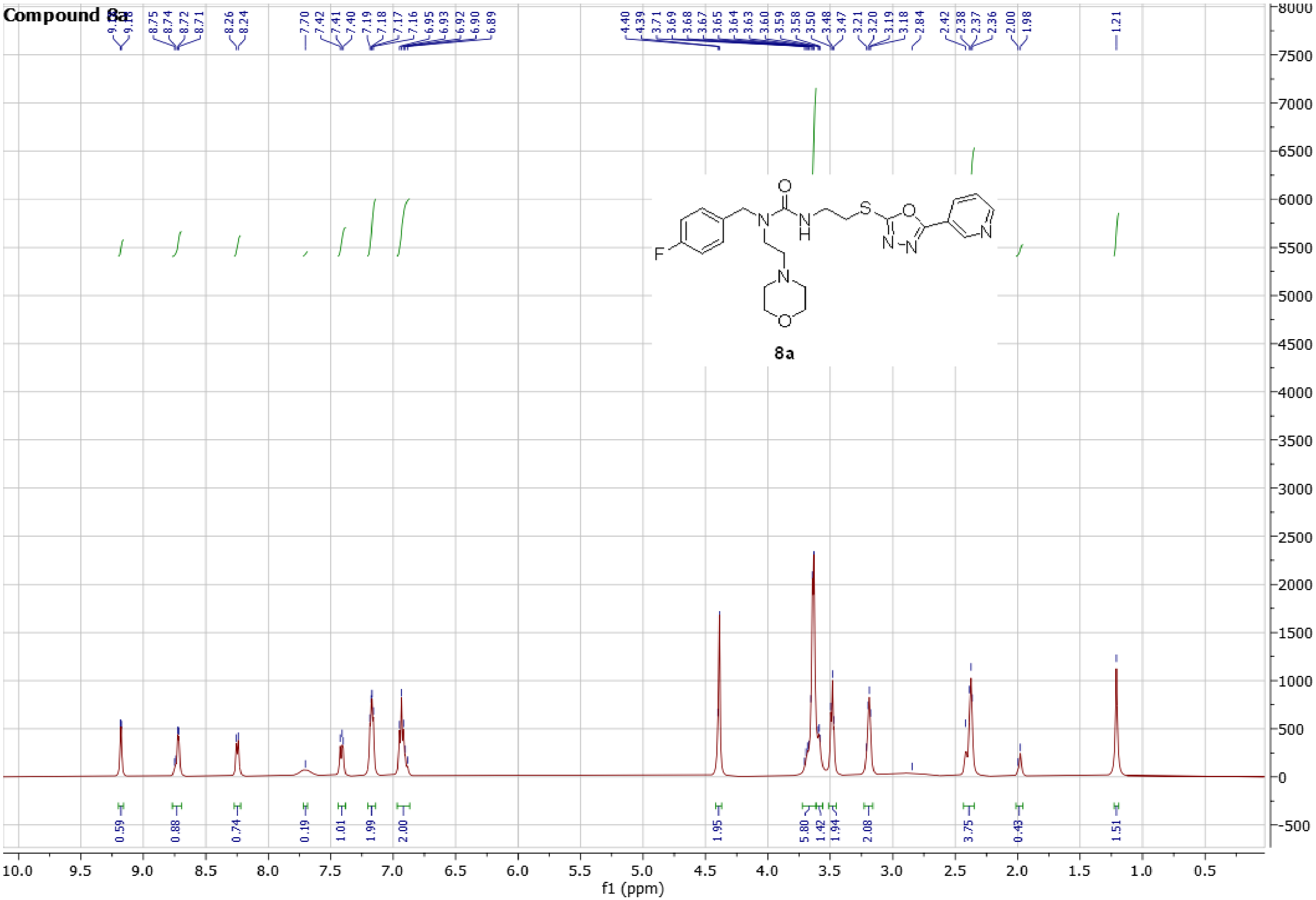

Figure 1. 1H-NMR for compound **8a**

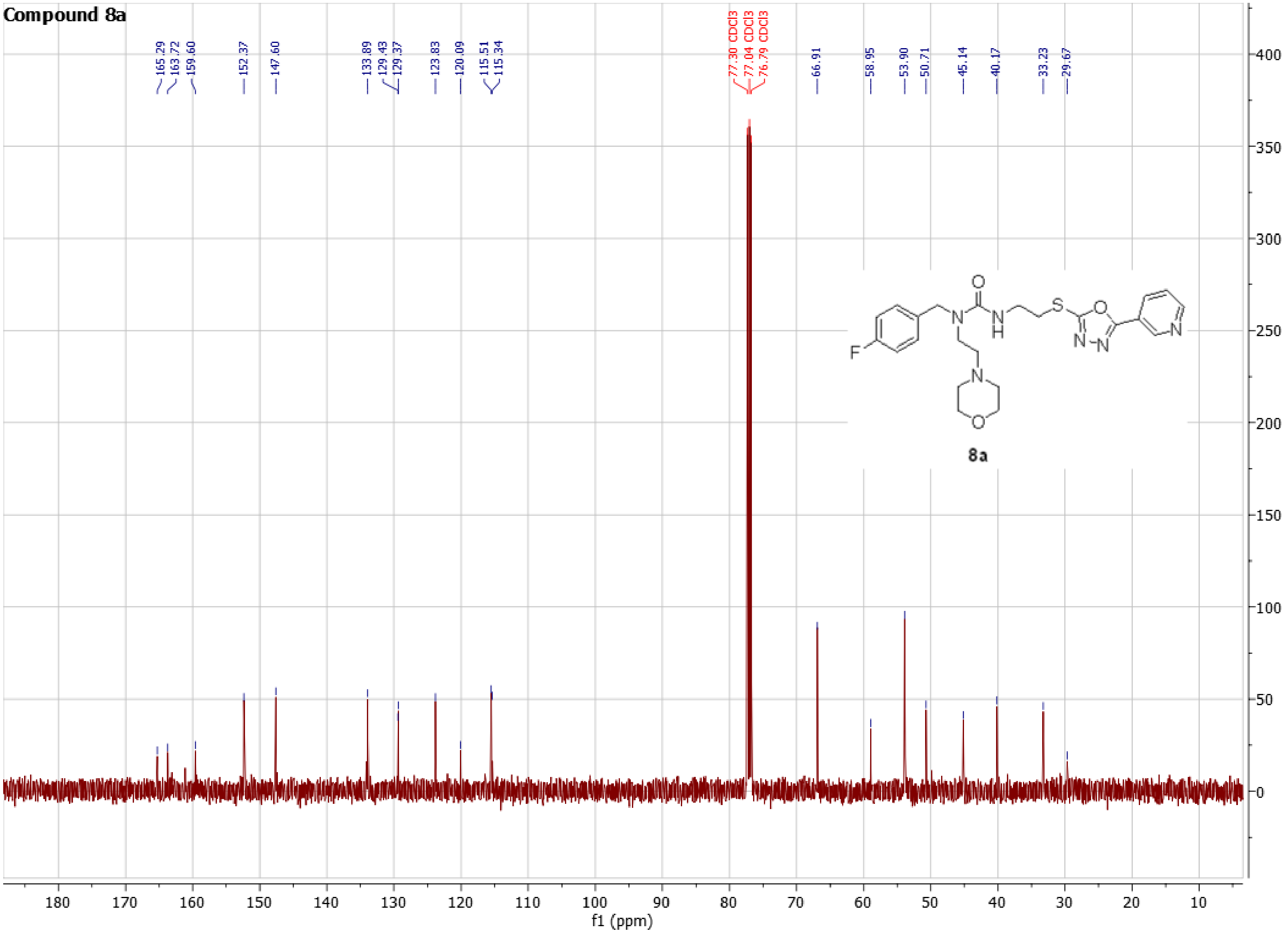

0

Figure 2. 13C-NMR for compound **8a**

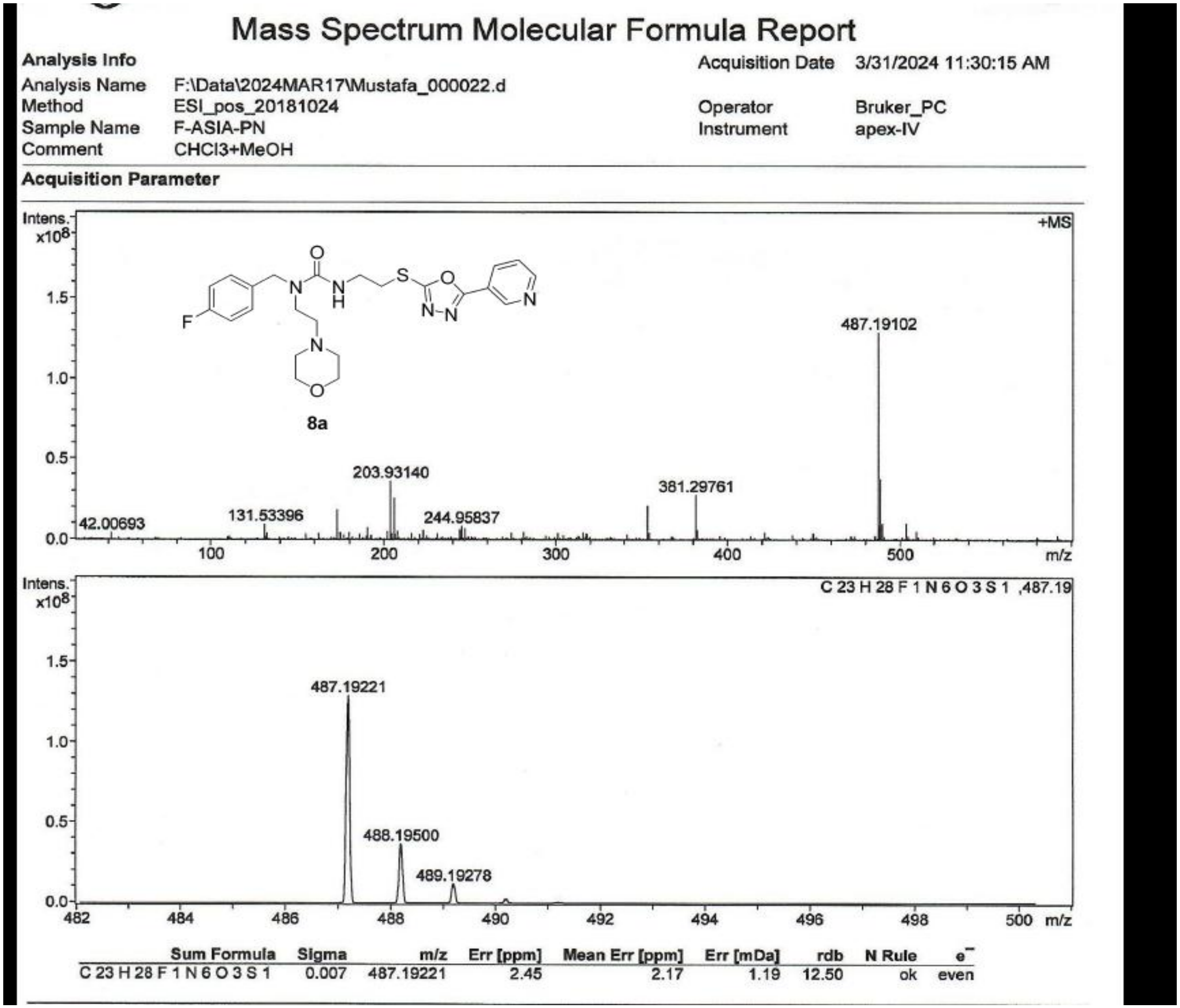

Figure 3. Mass spectroscopy of high resolution for compound **8a**

Mass Spectrum List Report

Analysis Info

Analysis Name	F:\Data\2024MAR17\Mustafa_000022.d	Acquisition Date	3/31/2024 11:30:15 AM
Method	ESI_pos_20181024	Operator	Bruker_PC
Sample Name	F-ASIA-PN	Instrument	apex-IV
Comment	CHCl3+MeOH		

Acquisition Parameter

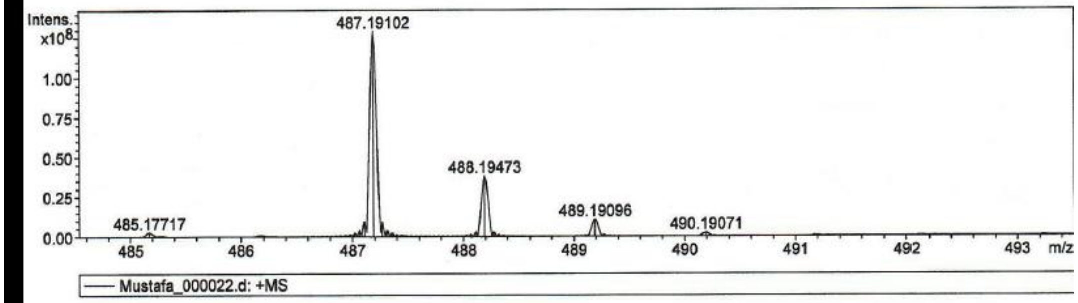

#	m/z	I	I %
1	42.00693	4288185	3.3
2	131.53396	9510972	7.4
3	172.95698	18733775	14.5
4	174.95211	4299269	3.3
5	179.98615	4313276	3.3
6	190.96755	7295230	5.7
7	201.97636	5078863	3.9
8	203.93140	36113029	28.0
9	205.92854	25658201	19.9
10	207.92570	5546856	4.3
11	223.01308	6023705	4.7
12	244.10072	6656253	5.2
13	244.95837	8234552	6.4
14	246.95571	6811328	5.3
15	274.27497	4396692	3.4
16	281.16669	4869802	3.8
17	315.83287	4341806	3.4
18	353.26646	20997531	16.3
19	354.27027	4551091	3.5
20	381.29761	27825567	21.6
21	382.30156	6393666	5.0
22	421.17497	4574914	3.6
23	449.21139	4067657	3.2
24	487.11063	9241160	7.2
25	487.19102	128765033	100.0
26	487.27092	9424006	7.3
27	488.19473	37579755	29.2
28	489.19096	10270171	8.0
29	503.18639	10470906	8.1
30	509.17385	5394664	4.2

Figure 4. Mass spectroscopy of high resolution for compound **8a**

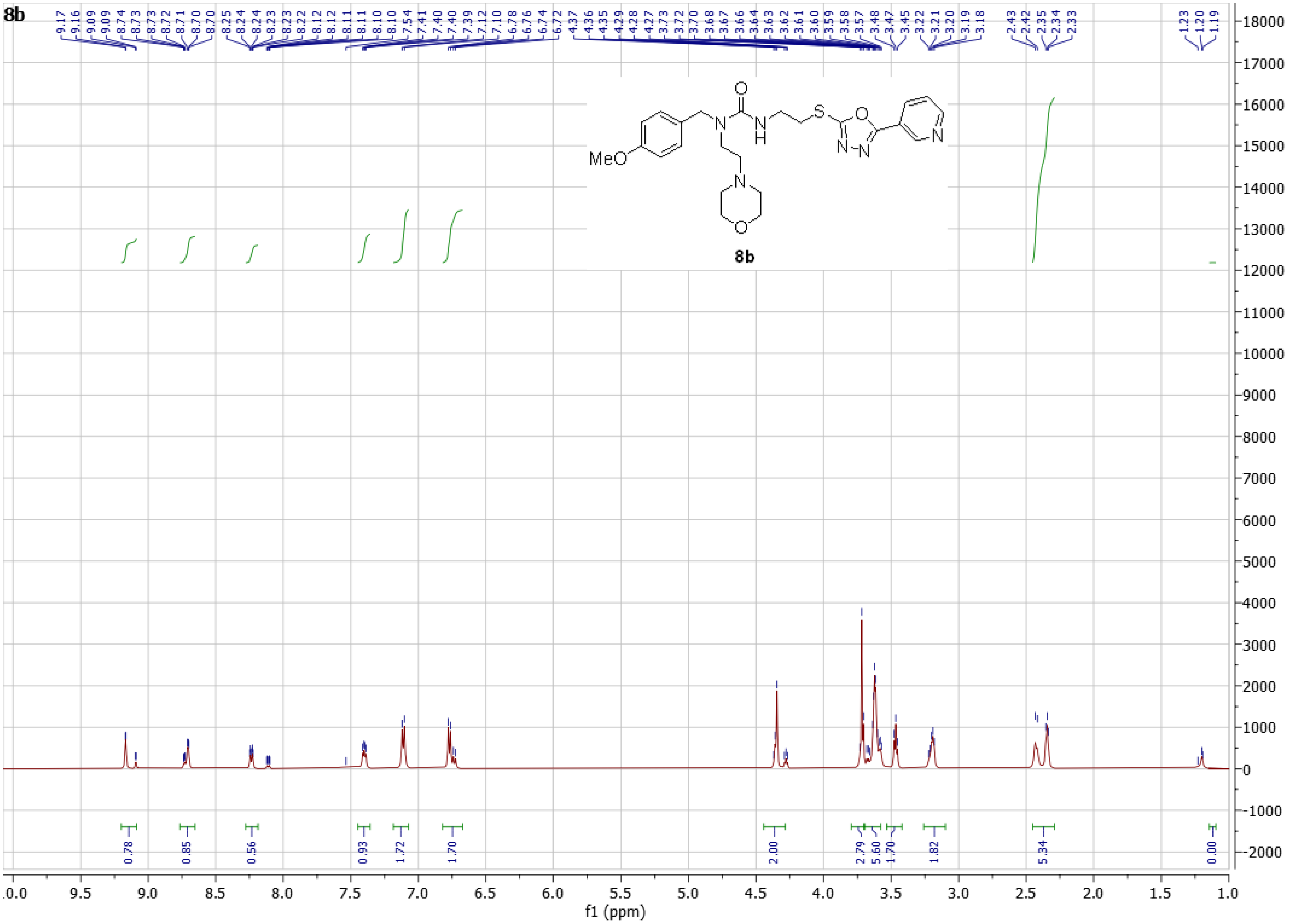

Figure 5. 1H-NMR for compound **8b**

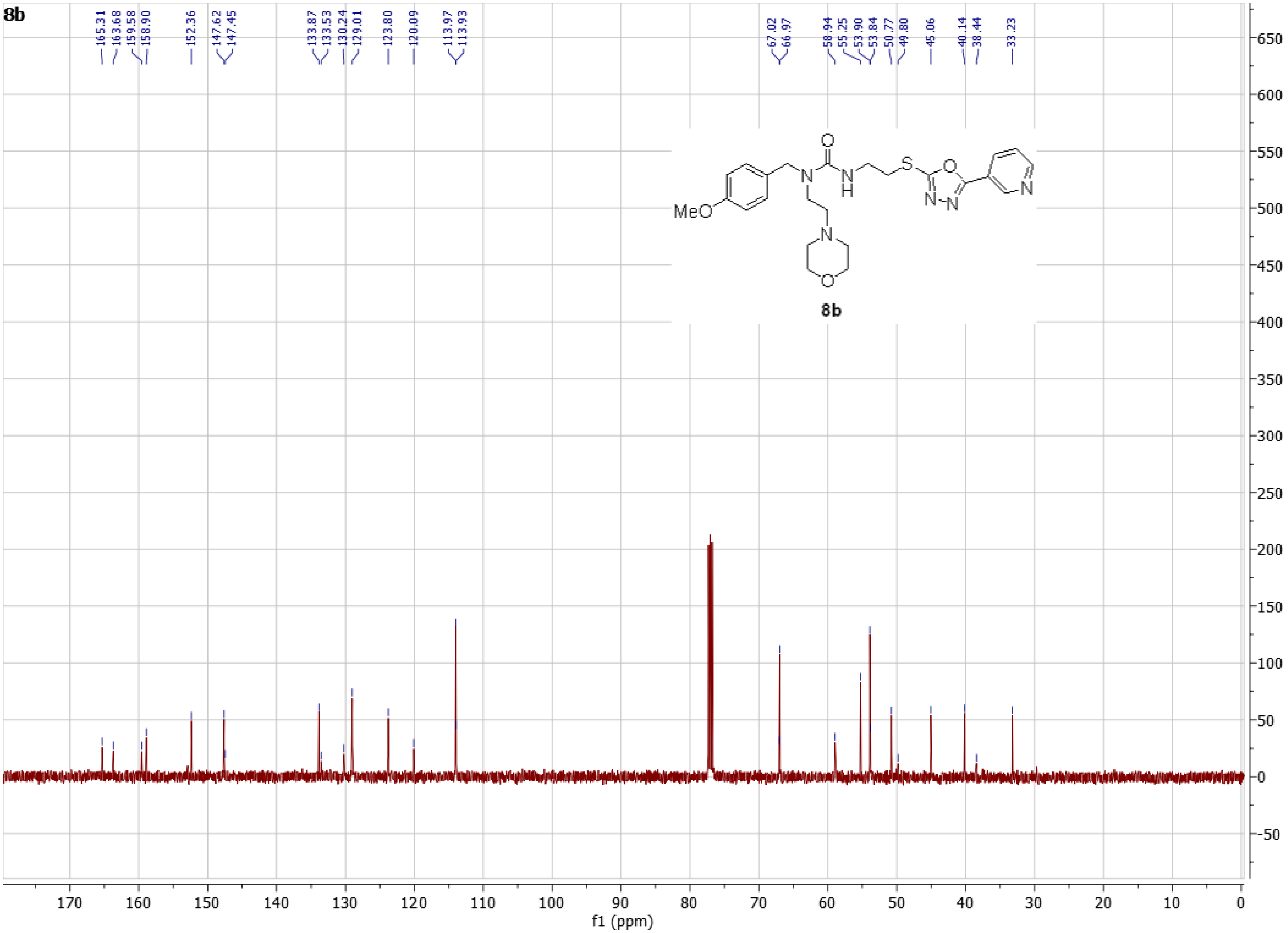

Figure 6. 13C-NMR for compound **8b**

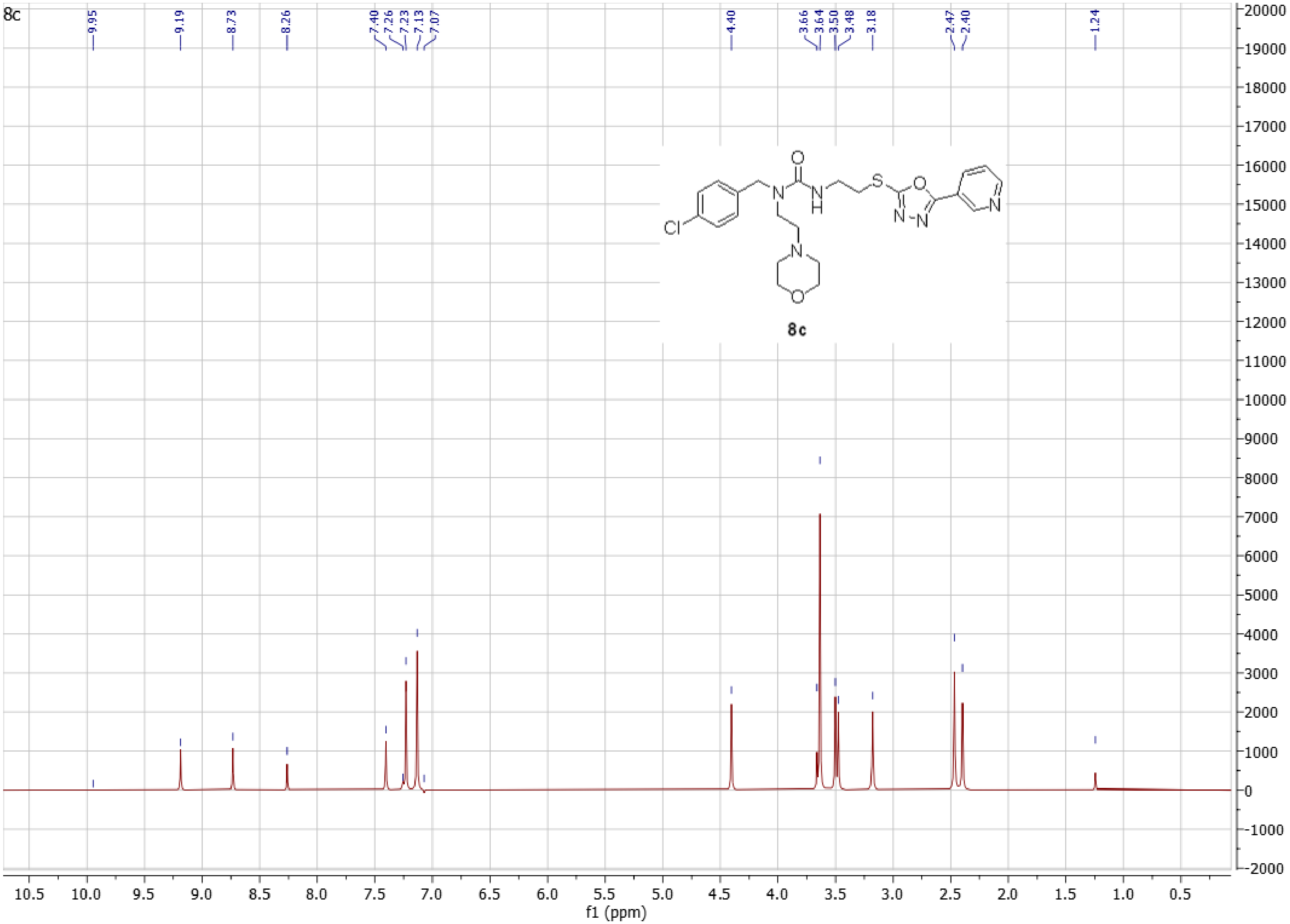

Figure 7. 1H-NMR for compound **8c**

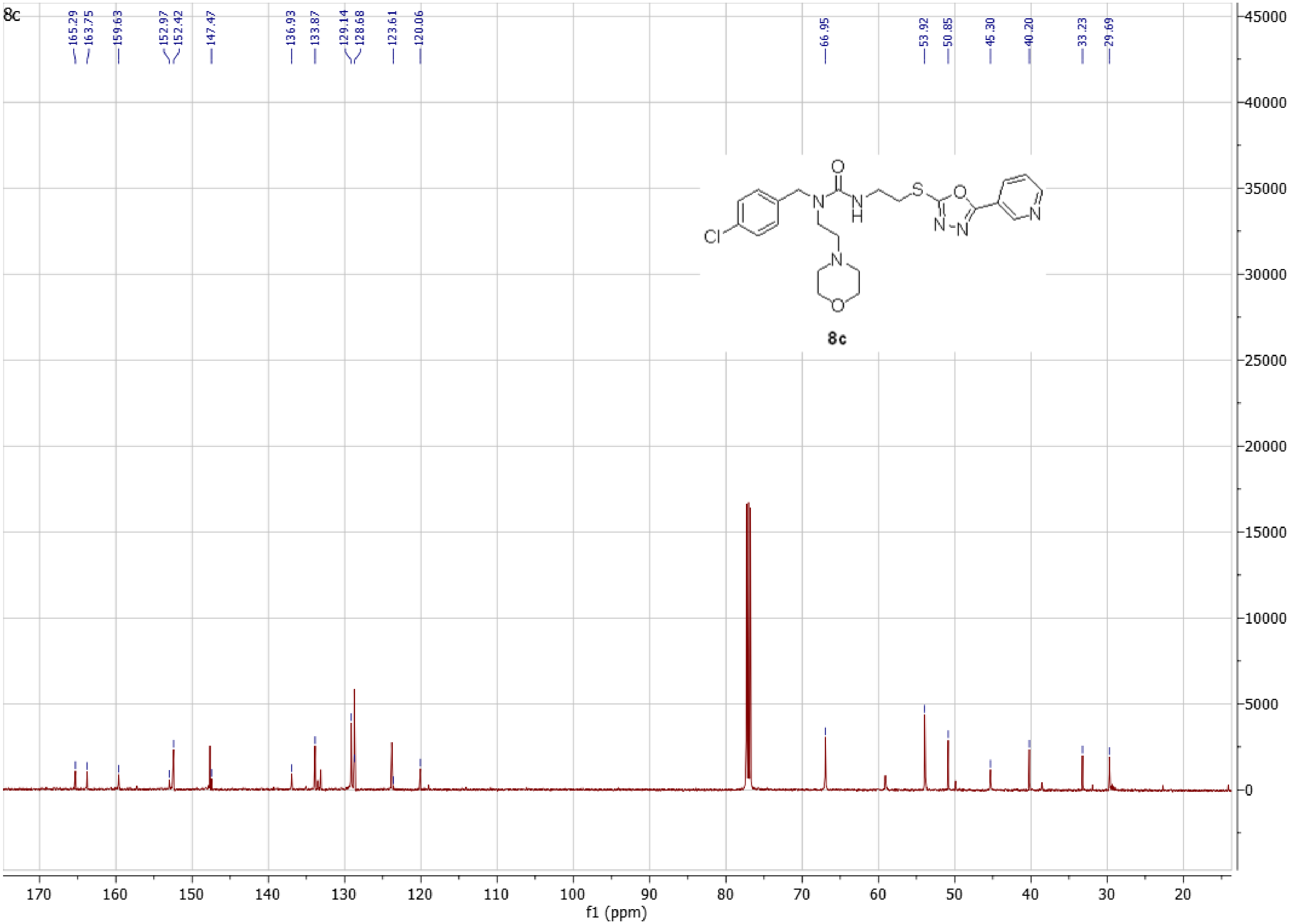

Figure 8. 13C-NMR for compound **8c**

Mass Spectrum Molecular Formula Report

Analysis Info

Analysis Name	F:\Data\2024MAY23\Mustafa_000062.d	Acquisition Date	7/4/2024 12:32:57 PM
Method	ESI_pos_20181024	Operator	Bruker_PC
Sample Name	Cl-PN	Instrument	apex-IV
Comment	CHCl3+ACN		

Acquisition Parameter

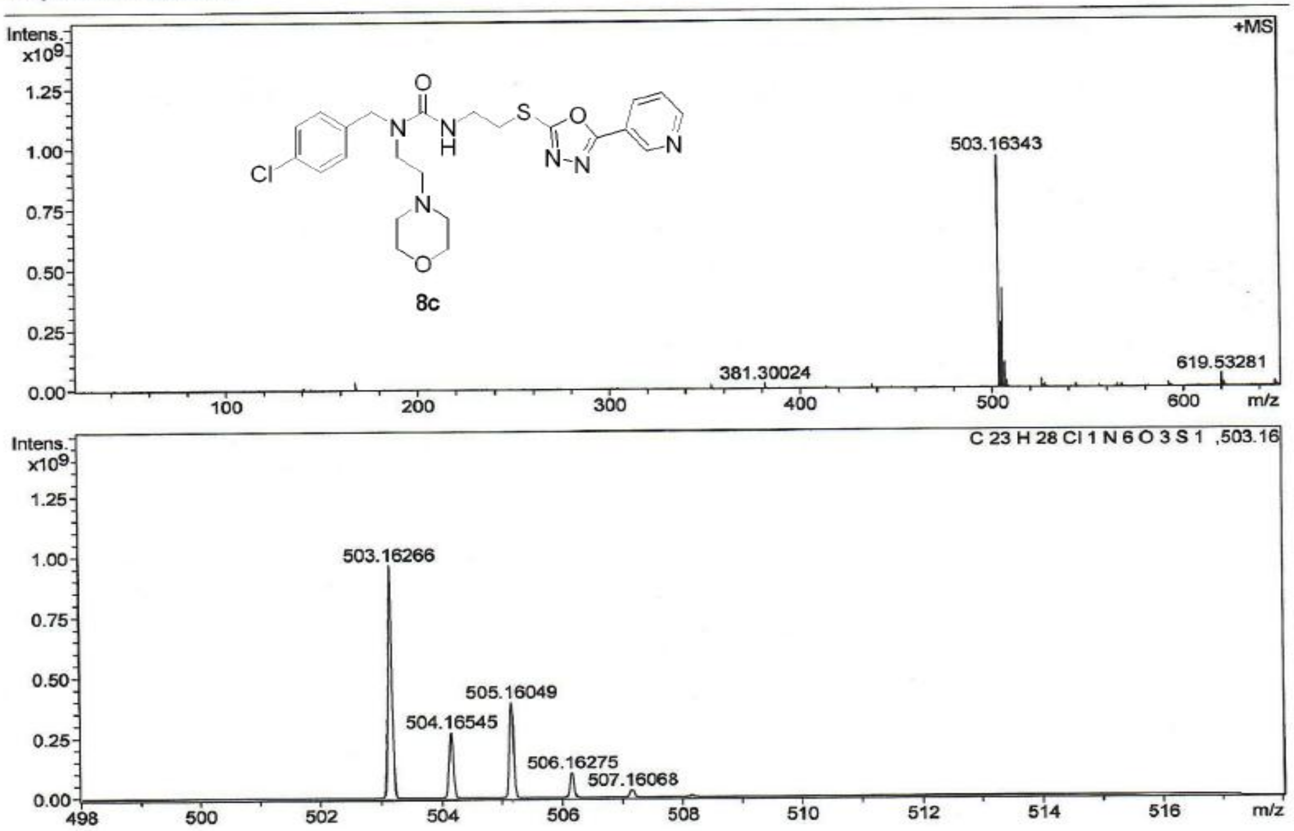

Sum Formula	Sigma	m/z	Err [ppm]	Mean Err [ppm]	Err [mDa]	rdb	N Rule	e⁻
C 23 H 28 Cl 1 N 6 O 3 S 1	0.007	503.16266	-1.53	-3.35	-0.77	12.50	ok	even

Figure 9. Mass spectroscopy of high resolution for compound **8c**

Mass Spectrum List Report

Analysis Info

Analysis Name	F:\Data\2024MAY23\Mustafa_000062.d	Acquisition Date	7/4/2024 12:32:57 PM
Method	ESI_pos_20181024	Operator	Bruker_PC
Sample Name	Cl-PN	Instrument	apex-IV
Comment	CHCl3+ACN		

Acquisition Parameter

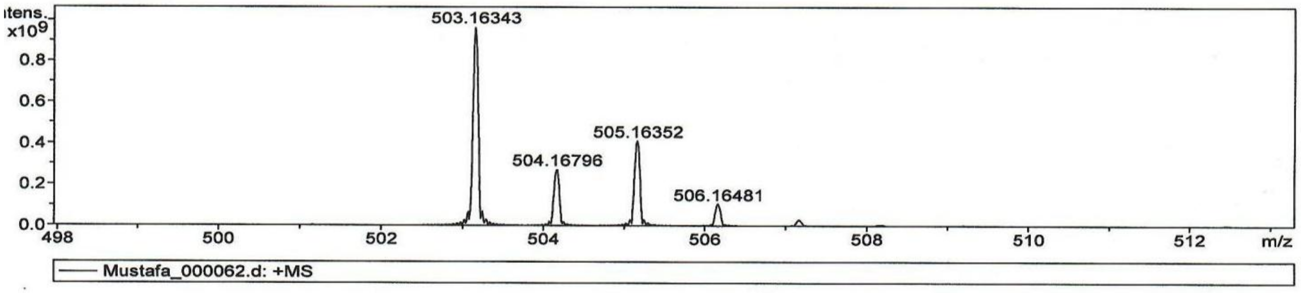

#	m/z	I	I %
1	353.26902	17120562	1.8
2	381.30024	22779419	2.4
3	437.19672	11952764	1.2
4	502.98608	15660850	1.6
5	503.03167	28651321	3.0
6	503.07795	70227775	7.3
7	503.16343	964466506	100.0
8	503.24930	68679511	7.1
9	503.29555	28589917	3.0
10	503.34102	15552355	1.6
11	503.38616	9857897	1.0
12	504.08222	19178420	2.0
13	504.16796	272267196	28.2
14	504.25408	19246019	2.0
15	505.03026	12026864	1.2
16	505.07706	29116402	3.0
17	505.16352	410671093	42.6
18	505.24945	30158839	3.1
19	505.29826	13498361	1.4
20	506.16481	104824826	10.9
21	507.16489	29290700	3.0
22	525.14906	33579053	3.5
23	527.14720	13521637	1.4
24	543.20755	10166516	1.1
25	565.08984	9903179	1.0
26	591.50444	18365267	1.9
27	619.53281	54350936	5.6
28	620.53303	20817927	2.2
29	647.55553	24596765	2.6
30	648.55955	9951058	1.0

Figure 10. Mass spectroscopy of high resolution for compound **8c**

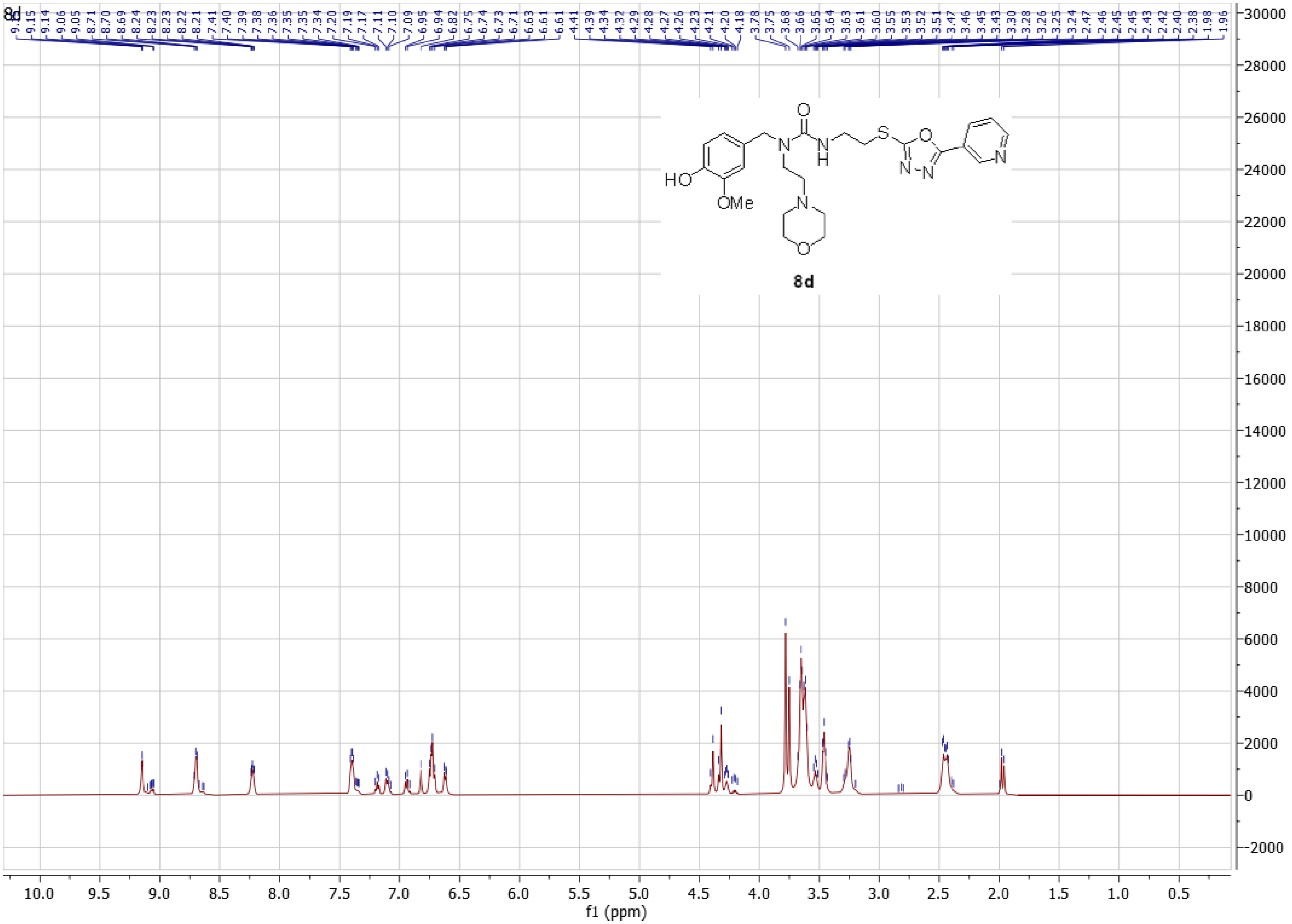

Figure 11. 1H-NMR for compound **8d**

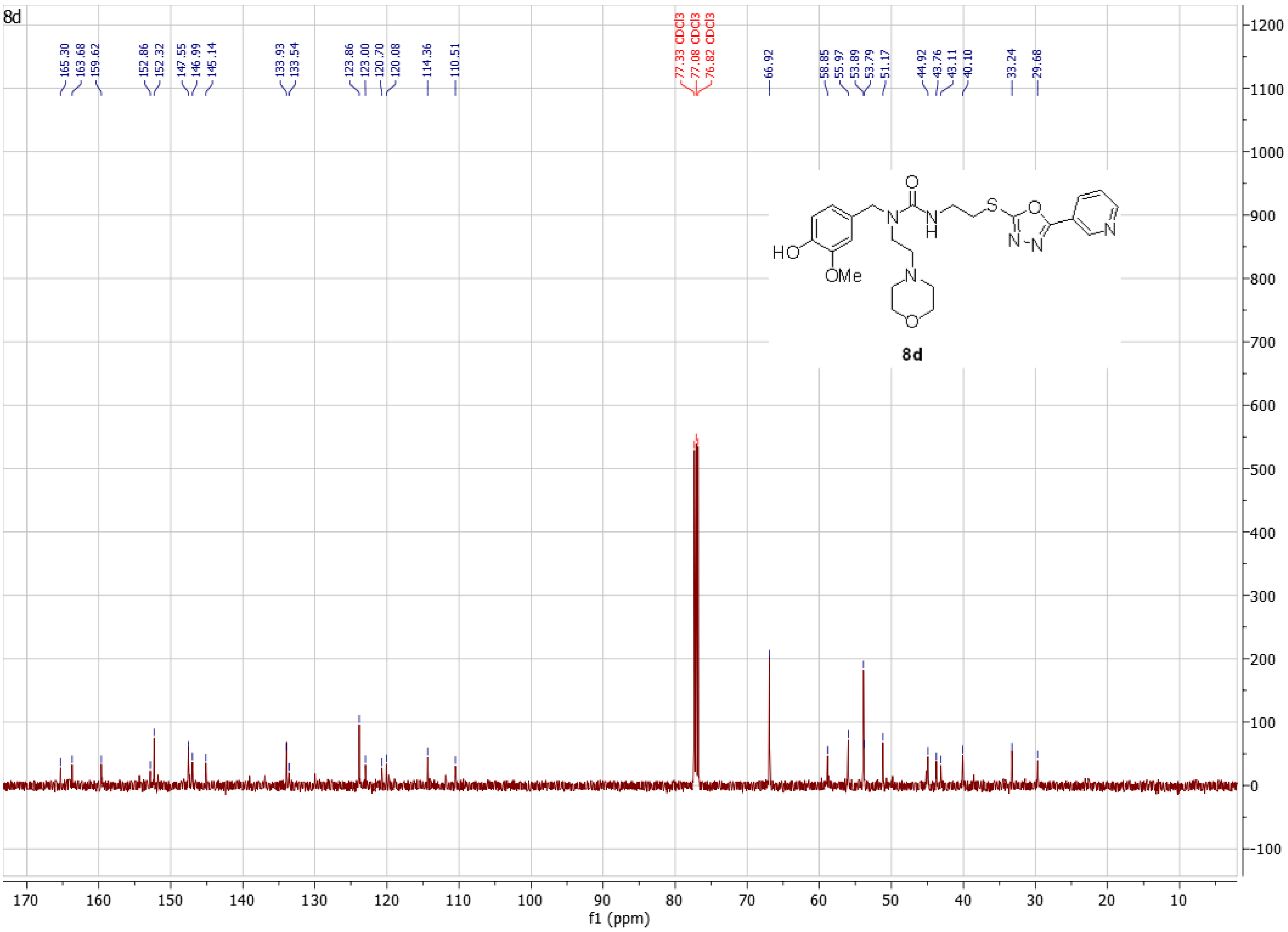

Figure 12. 13C-NMR for compound **8d**

Mass Spectrum Molecular Formula Report

Analysis Info | Acquisition Date 4/16/2024 3:15:37 PM

Analysis Name	F:\Data\2024MAR17\Mustafa_000043.d		
Method	ESI_pos_20181024	Operator	Bruker_PC
Sample Name	V-ASIA-PN	Instrument	apex-IV
Comment	MeOH		

Acquisition Parameter

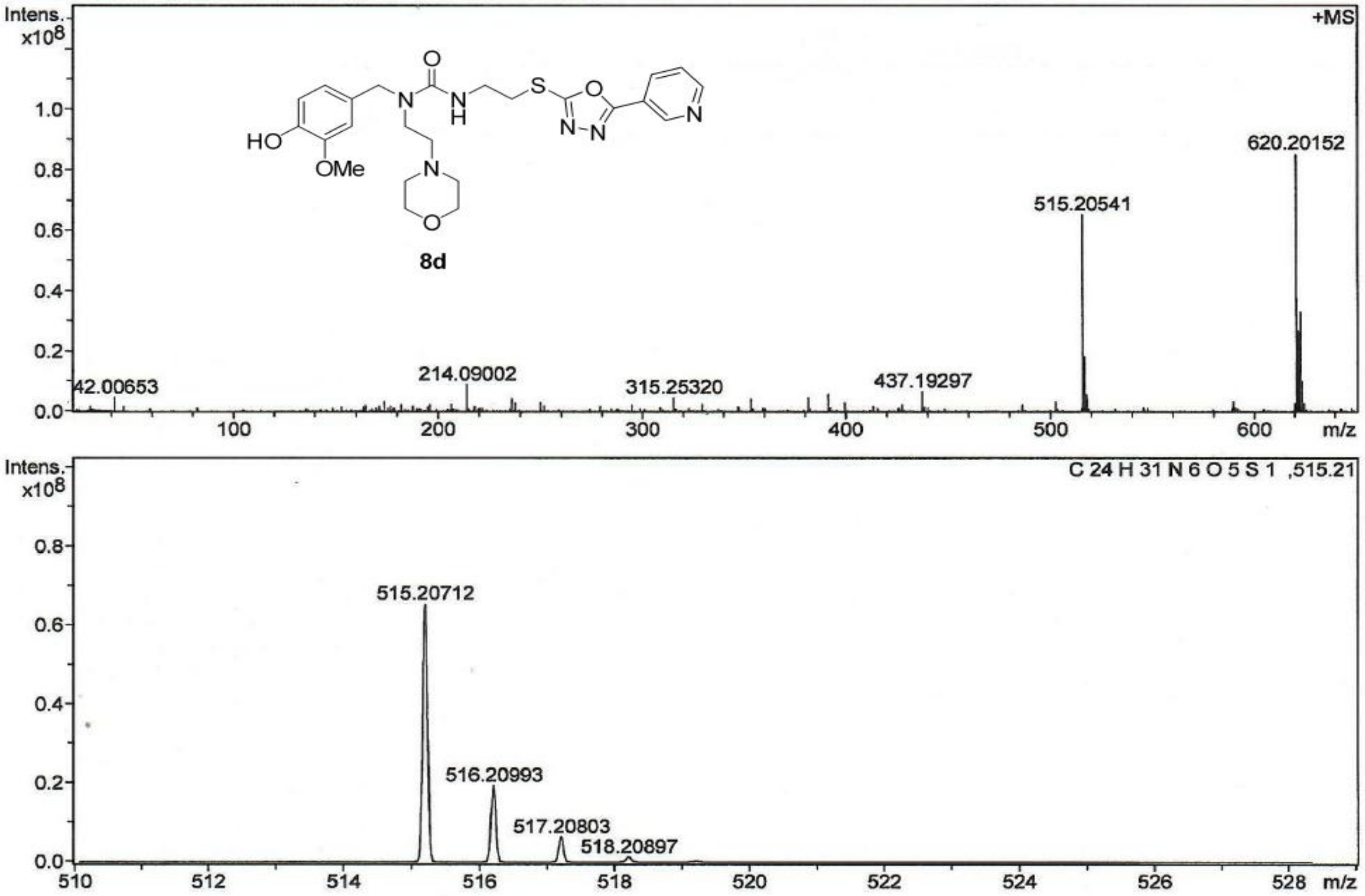

Sum Formula	Sigma	m/z	Err [ppm]	Mean Err [ppm]	Err [mDa]	rdb	N Rule	e⁻
C 24 H 31 N 6 O 5 S 1	0.008	515.20712	3.31	3.45	1.71	12.50	ok	even

Figure 13. Mass spectroscopy of high resolution for compound **8d**

Mass Spectrum List Report

Analysis Info

Analysis Name	F:\Data\2024MAR17\Mustafa_000043.d	Acquisition Date	4/16/2024 3:15:37 PM
Method	ESI_pos_20181024	Operator	Bruker_PC
Sample Name	V-ASIA-PN	Instrument	apex-IV
Comment	MeOH		

Acquisition Parameter

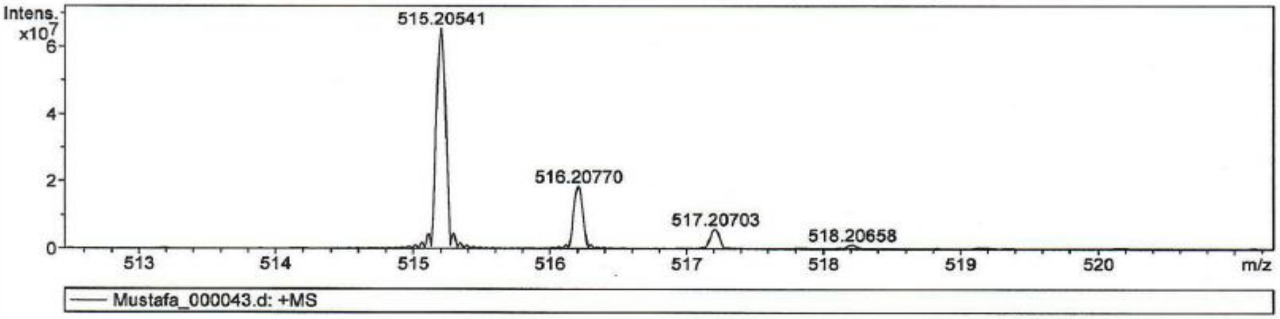

#	m/z	I	I %
1	42.00653	4824141	5.7
2	173.98517	3465297	4.1
3	214.09002	9139283	10.7
4	235.96736	4464499	5.2
5	237.96444	2962428	3.5
6	249.98298	3043495	3.6
7	315.25320	4649725	5.5
8	329.23229	2604331	3.1
9	353.26620	4262049	5.0
10	381.29705	4564495	5.4
11	391.28396	5864096	6.9
12	399.25007	2996526	3.5
13	437.19297	6851993	8.0
14	502.19127	3350720	3.9
15	515.11604	4642734	5.4
16	515.20541	65386143	76.7
17	515.29549	4575373	5.4
18	516.20770	18464098	21.7
19	517.20703	5850009	6.9
20	589.19863	3502526	4.1
21	619.52521	2813382	3.3
22	620.00066	2447718	2.9
23	620.07109	6164345	7.2
24	620.20152	85282205	100.0
25	620.33135	6139324	7.2
26	620.40173	2619854	3.1
27	621.20385	27036315	31.7
28	622.19864	33519755	39.3
29	623.20138	10157424	11.9
30	624.19991	2841984	3.3

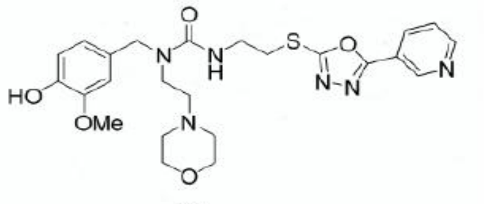

8d

Figure 14. Mass spectroscopy of high resolution for compound **8d**

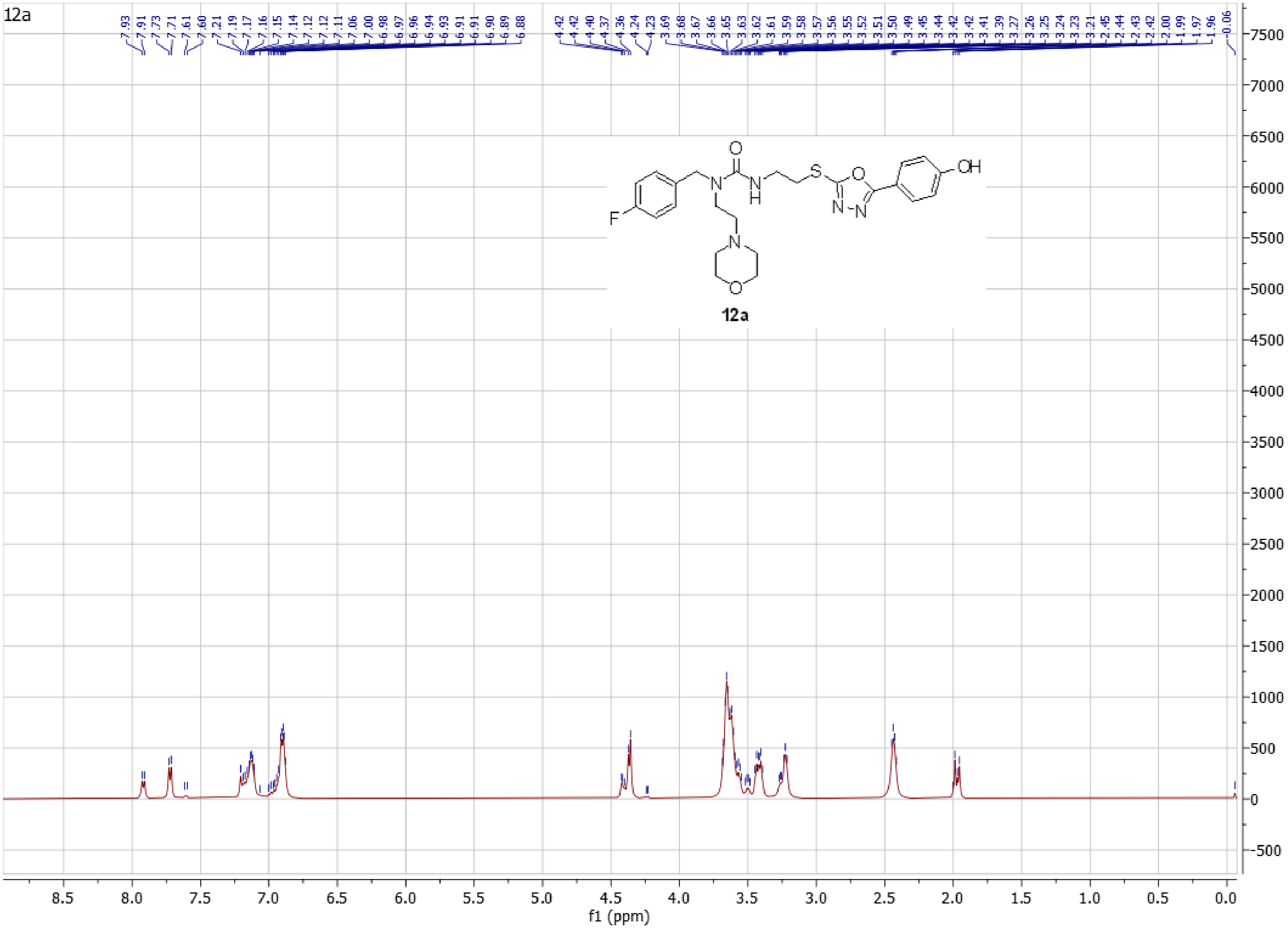

Figure 15. 1H-NMR for compound **12a**

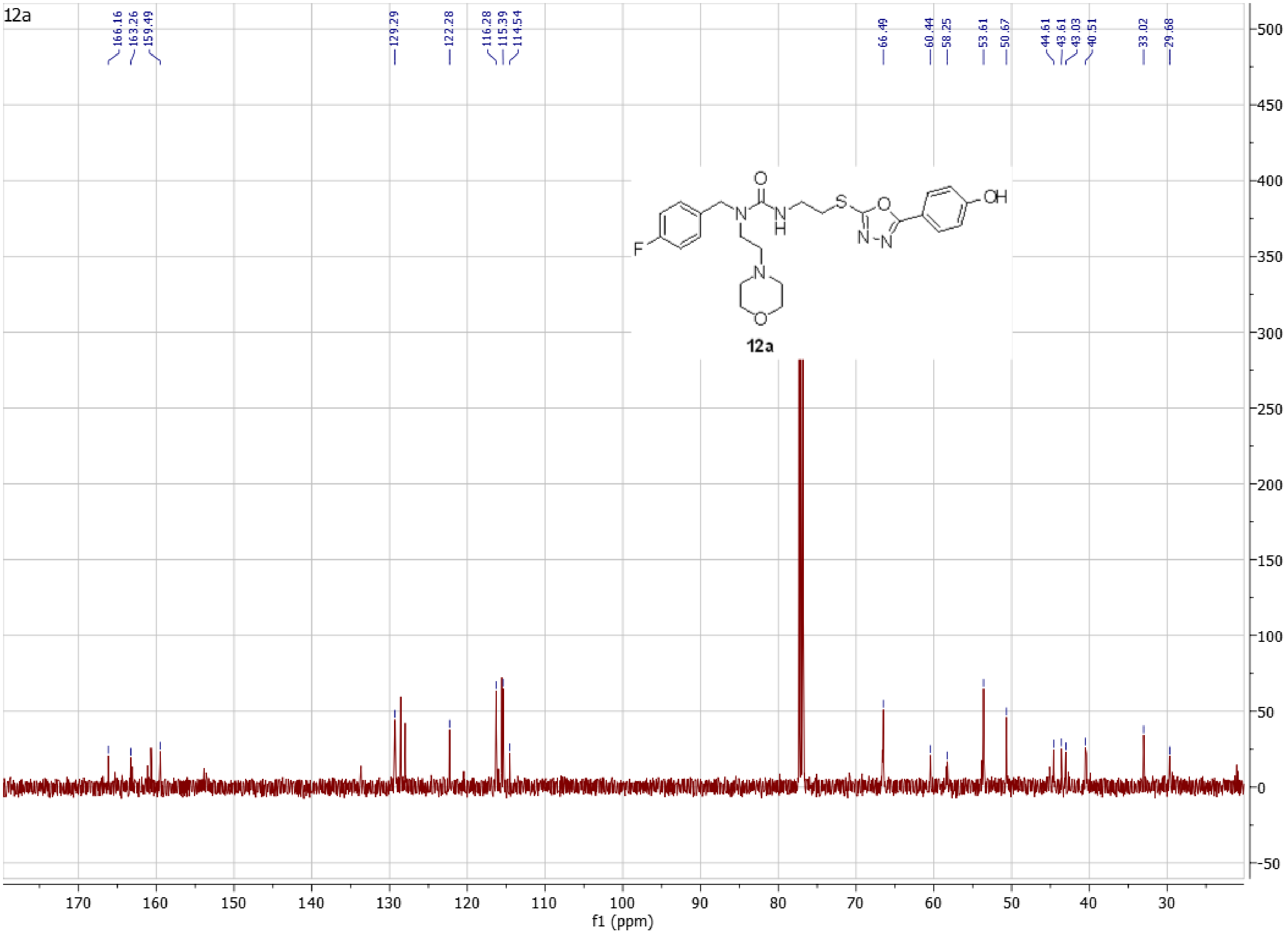

Figure 16. 13C-NMR for compound **12a**

Mass Spectrum Molecular Formula Report

Analysis Info | Acquisition Date 4/3/2024 10:07:31 AM

Analysis Name	F:\Data\2024MAR17\Mustafa_000040.d		
Method	ESI_pos_20181024	Operator	Bruker_PC
Sample Name	F-ASIA-OH	Instrument	apex-IV
Comment	CHCl3+MeOH		

Acquisition Parameter

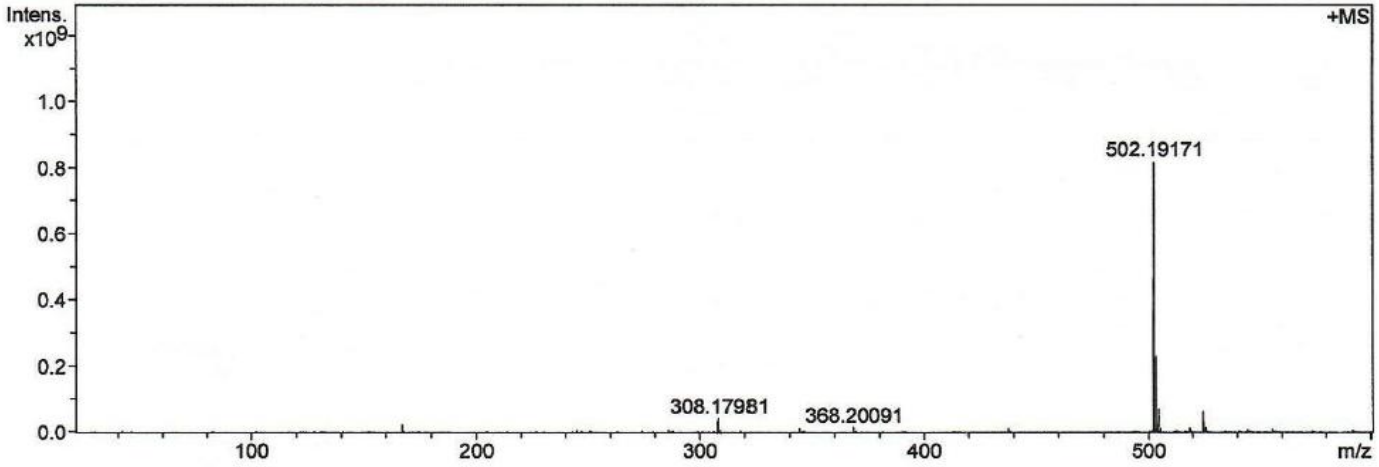

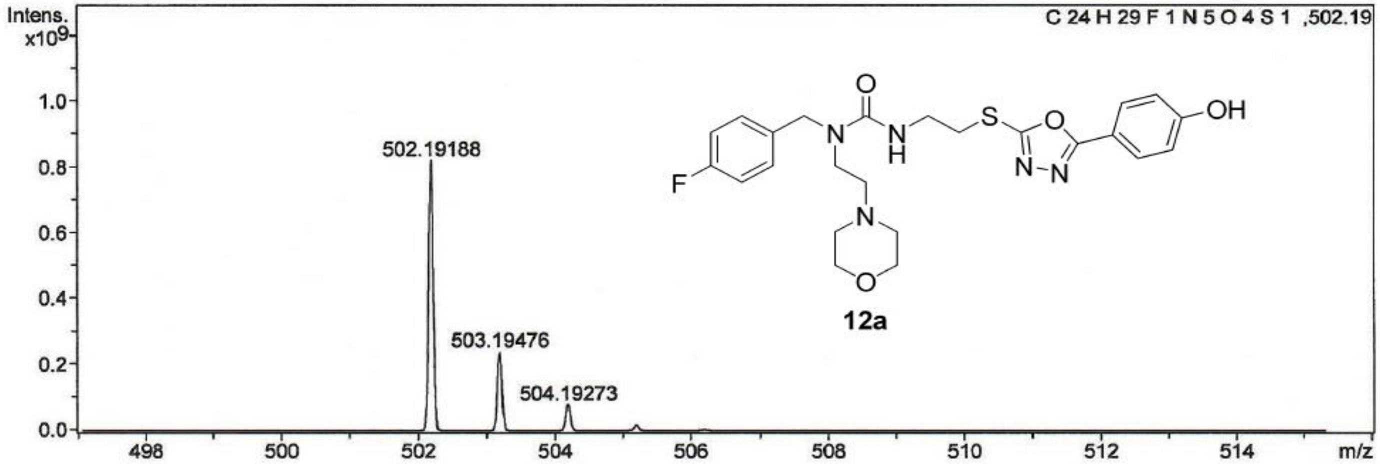

Sum Formula	Sigma	m/z	Err [ppm]	Mean Err [ppm]	Err [mDa]	rdb	N Rule	e⁻
C 24 H 29 F 1 N 5 O 4 S 1	0.004	502.19188	0.33	-2.17	0.17	12.50	ok	even

Figure 17. Mass spectroscopy of high resolution for compound **12a**

Mass Spectrum List Report

**nalysis Info**		Acquisition Date	4/3/2024 10:07:31 AM
nalysis Name	F:\Data\2024MAR17\Mustafa_000040.d		
lethod	ESI_pos_20181024	Operator	Bruker_PC
ample Name	F-ASIA-OH	Instrument	apex-IV
omment	CHCl3+MeOH		

Acquisition Parameter

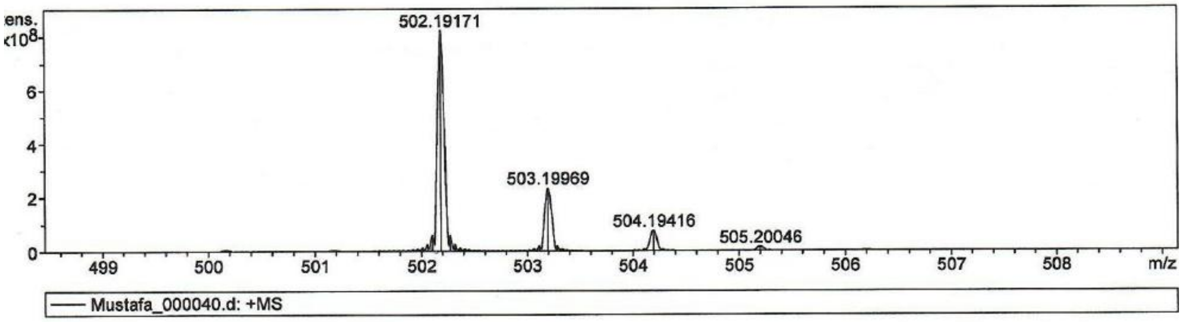

#	m/z	I	I %
1	244.95938	6096061	0.7
2	285.98399	6609077	0.8
3	308.17981	44836873	5.5
4	309.18375	8090824	1.0
5	344.15690	12978952	1.6
6	368.20091	19121724	2.3
7	437.19733	12513443	1.5
8	501.96949	8452808	1.0
9	502.01460	13400281	1.6
10	502.05998	24380137	3.0
11	502.10620	58591994	7.1
12	502.19171	819573531	100.0
13	502.27674	59487031	7.3
14	502.32281	24442695	3.0
15	502.36831	13095771	1.6
16	502.41348	8313450	1.0
17	503.06832	7013954	0.9
18	503.11423	16916560	2.1
19	503.19969	233276523	28.5
20	503.28533	16857219	2.1
21	503.33186	6903697	0.8
22	504.19416	73640316	9.0
23	505.20046	13938135	1.7
24	518.19005	13973936	1.7
25	524.17692	64134882	7.8
26	525.18116	18643579	2.3
27	526.18348	5845009	0.7
28	544.21006	9776088	1.2
29	555.32844	11130237	1.4
30	591.50013	7337567	0.9

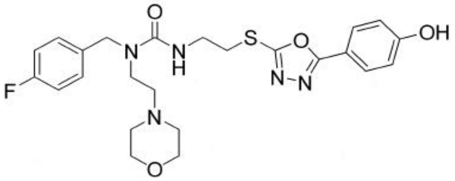

12a

Figure 18. Mass spectroscopy of high resolution for compound **12a**

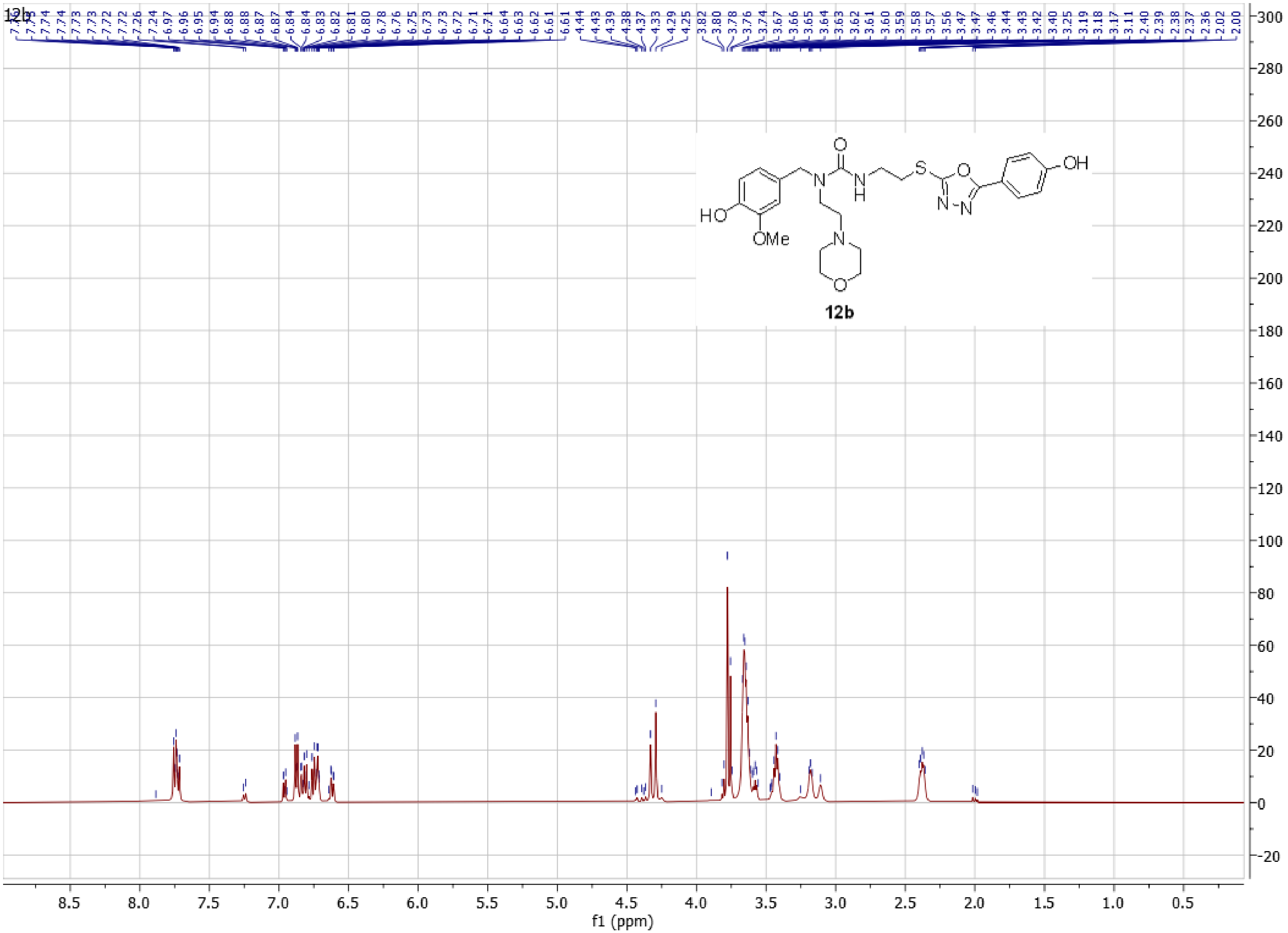

Figure 19. 1H-NMR for compound **12b**

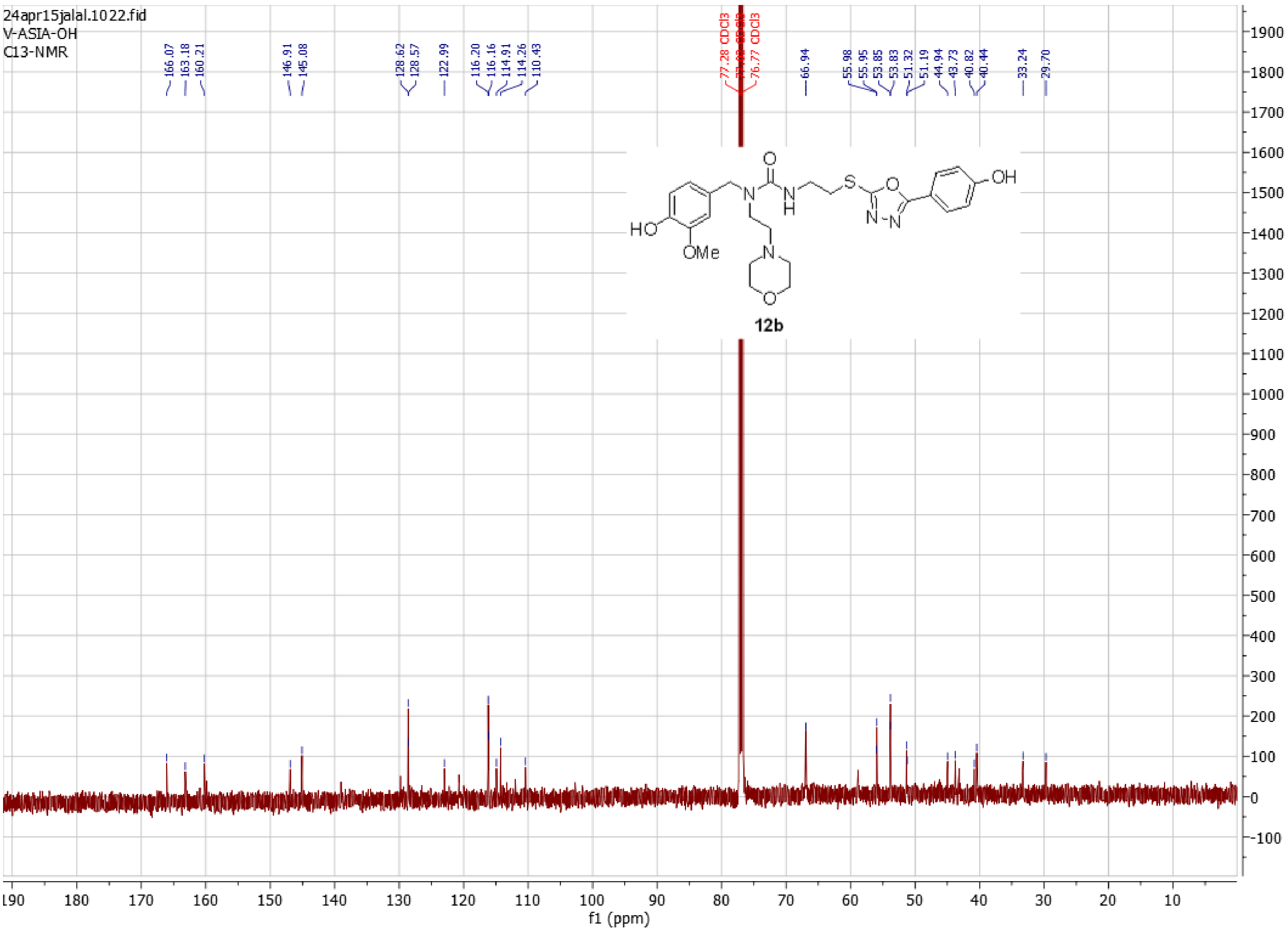

Figure 20. 13C-NMR for compound **12b**

Mass Spectrum Molecular Formula Report

Analysis Info

Analysis Name	F:\Data\2024APR23\Mustafa_000043.d	Acquisition Date	5/6/2024 10:17:18 AM
Method	ESI_pos_20181024	Operator	Bruker_PC
Sample Name	V-ASIA-OH	Instrument	apex-IV
Comment	CHCl3+ACETONE		

Acquisition Parameter

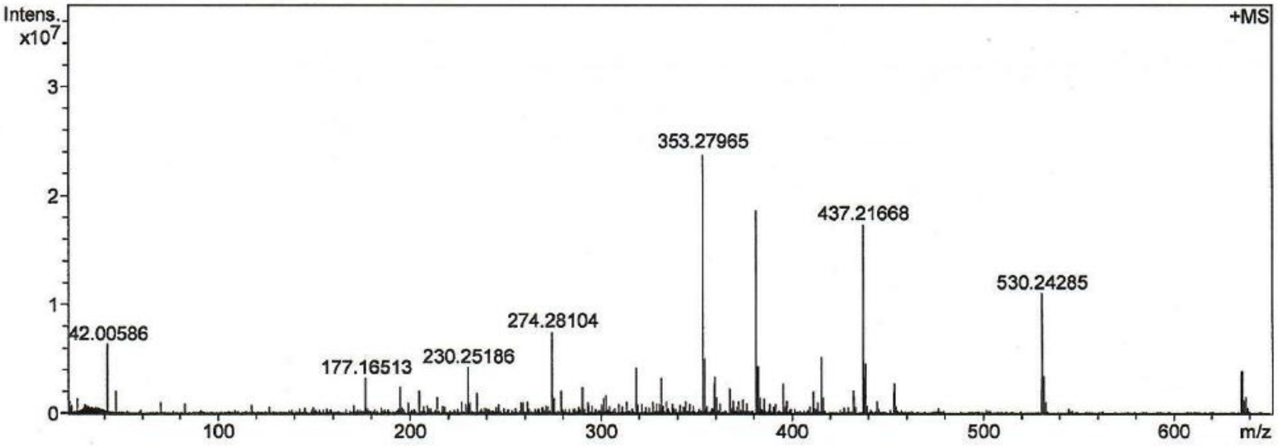

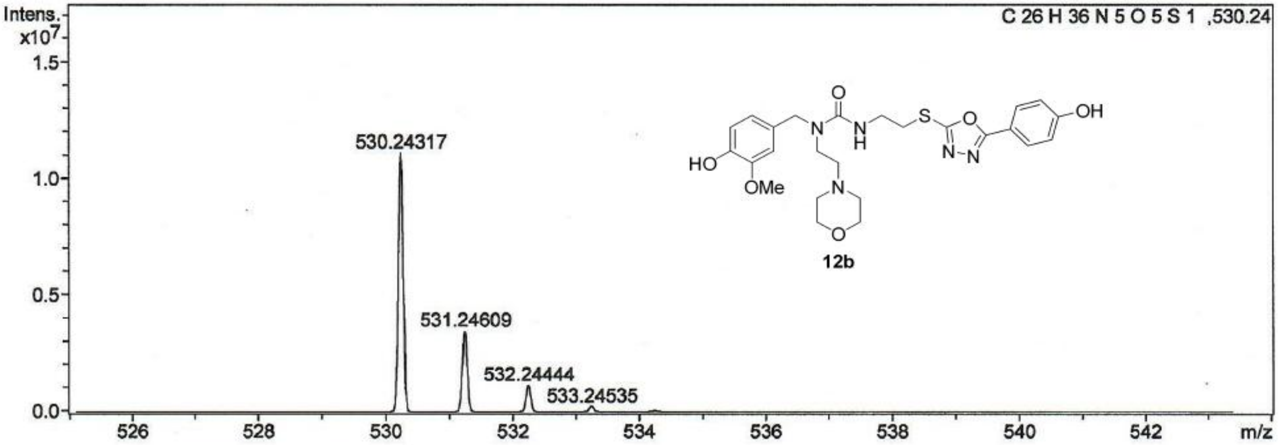

Sum Formula	Sigma	m/z	Err [ppm]	Mean Err [ppm]	Err [mDa]	rdb	N Rule	e⁻
C 26 H 36 N 5 O 5 S 1	0.004	530.24317	0.59	0.54	0.31	11.50	ok	even

Figure 21. Mass spectroscopy of high resolution for compound **12b**

Mass Spectrum List Report

**Analysis Info**		Acquisition Date	5/6/2024 10:17:18 AM
Analysis Name	F:\Data\2024APR23\Mustafa_000043.d		
Method	ESI_pos_20181024	Operator	Bruker_PC
Sample Name	V-ASIA-OH	Instrument	apex-IV
Comment	CHCl3+ACETONE		

Acquisition Parameter

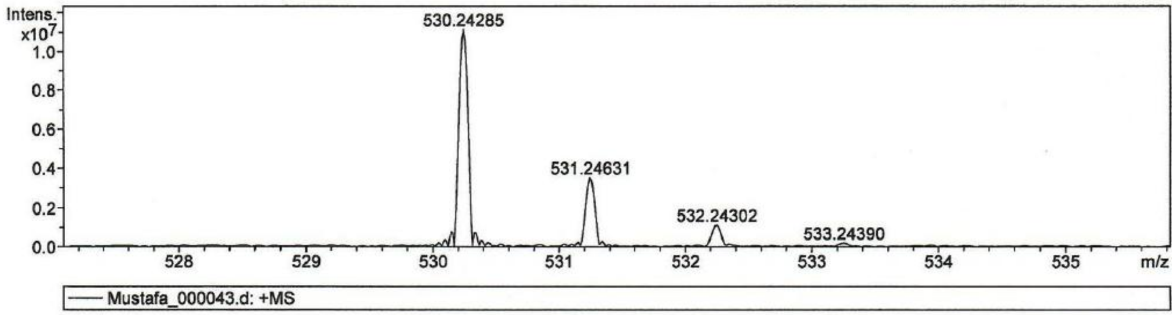

#	m/z	I	I %
1	42.00586	6411638	26.7
2	46.35253	2095215	8.7
3	177.16513	3301699	13.8
4	195.17654	2512829	10.5
5	205.08844	2105899	8.8
6	230.25186	4337184	18.1
7	235.20996	1945655	8.1
8	274.28104	7614413	31.7
9	279.16622	2115892	8.8
10	290.27699	2436336	10.2
11	302.31405	1675346	7.0
12	318.31065	4247471	17.7
13	331.29593	3380142	14.1
14	353.27965	23989958	100.0
15	353.32207	1733832	7.2
16	354.28324	5140014	21.4
17	359.32975	3408226	14.2
18	367.26033	2370432	9.9
19	381.31393	18805200	78.4
20	382.31735	4373564	18.2
21	395.29476	2810794	11.7
22	411.29231	2105654	8.8
23	415.23172	5275953	22.0
24	432.26066	2121467	8.8
25	437.21668	17437830	72.7
26	438.22021	4640239	19.3
27	453.19135	2814808	11.7
28	530.24285	11105119	46.3
29	531.24631	3507154	14.6
30	635.26179	3834108	16.0

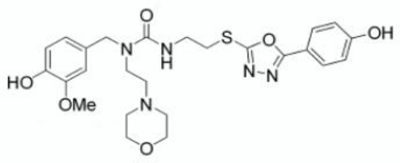

12b

Figure 22. Mass spectroscopy of high resolution for compound **12b**

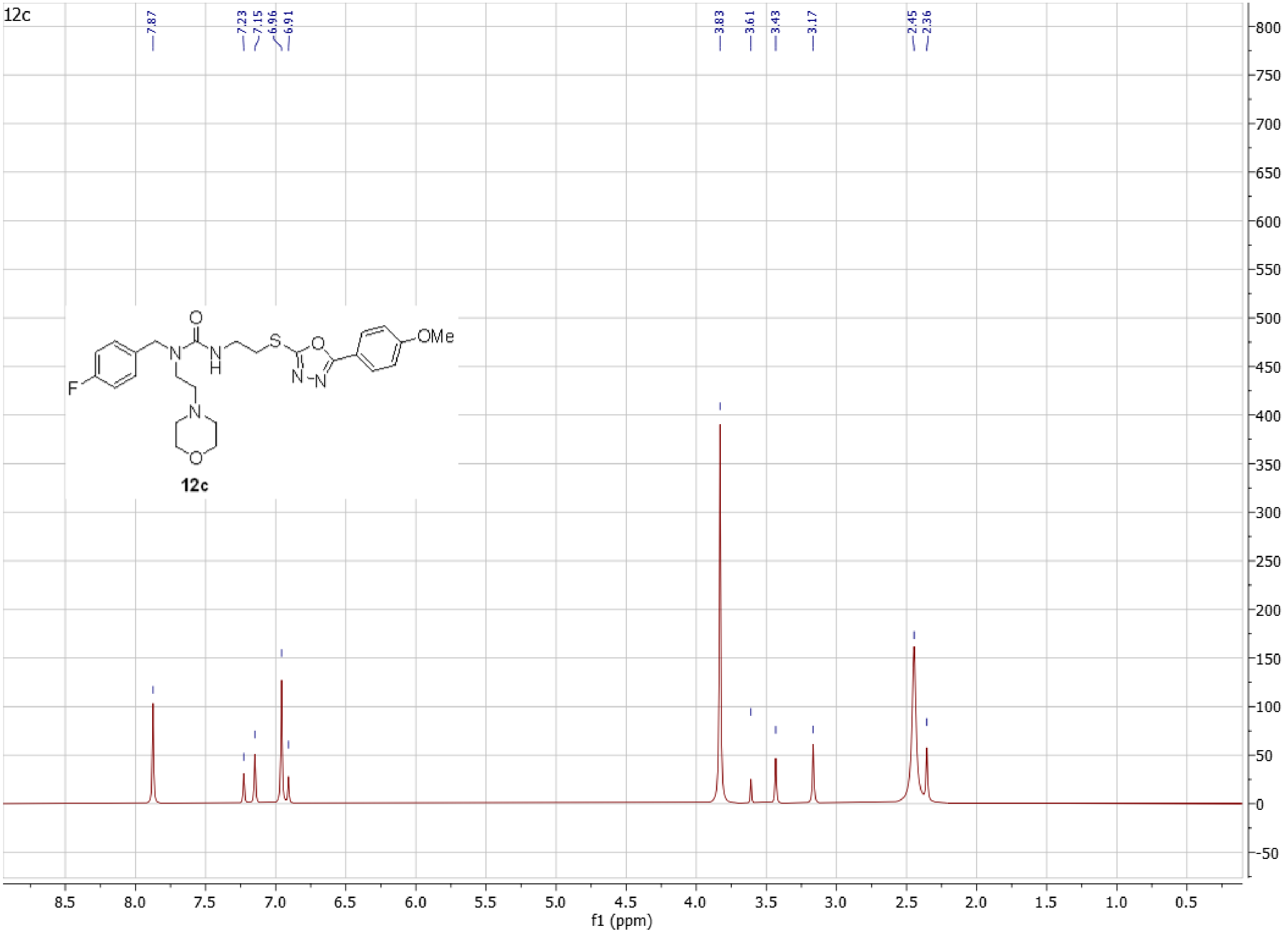

Figure 23. 1H-NMR for compound **12c**

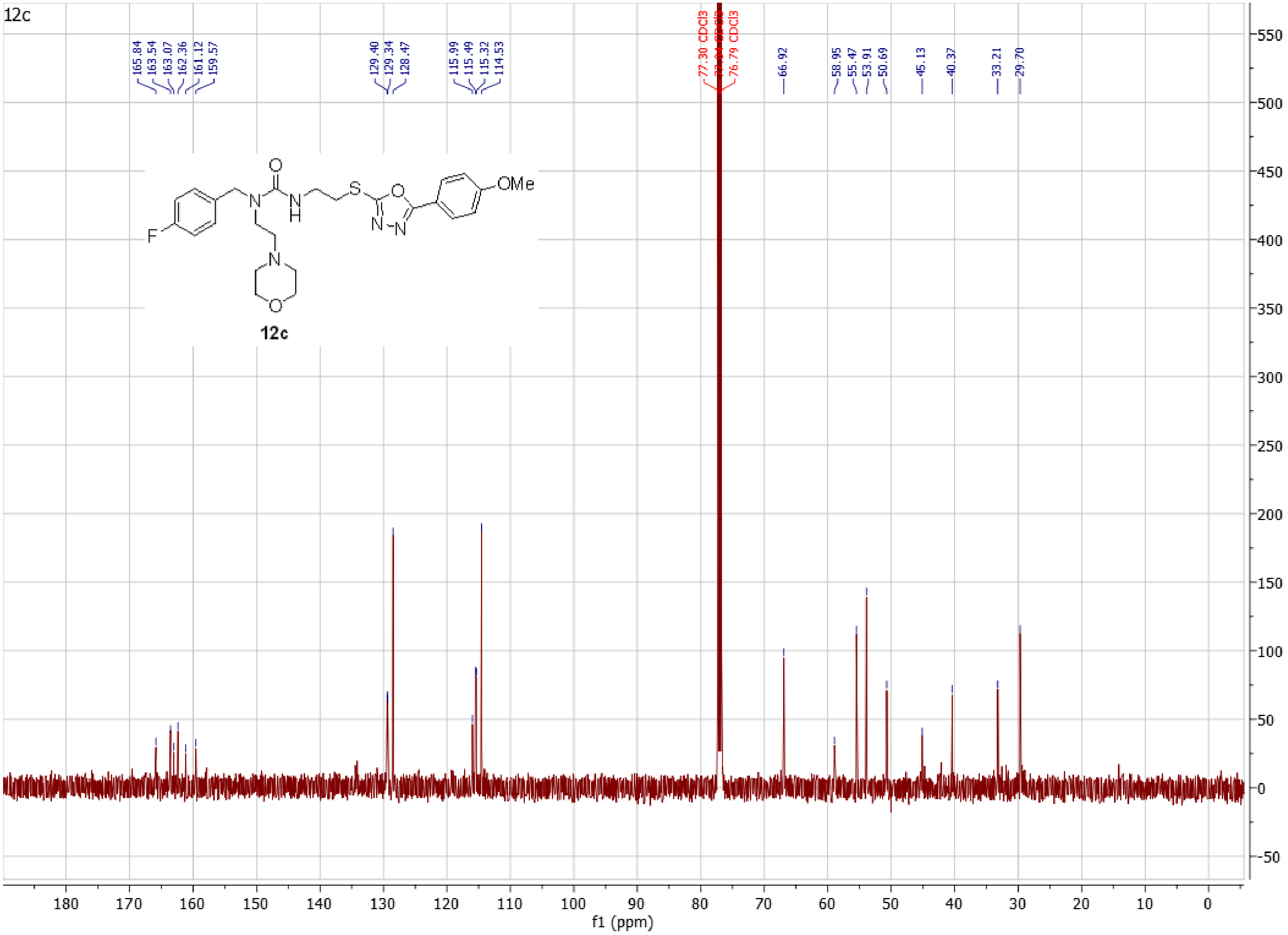

Figure 24. 13C-NMR for compound **12c**

Mass Spectrum Molecular Formula Report

Analysis Info

Analysis Name	F:\Data\2024MAY12\Mustafa_000049.d	Acquisition Date	5/22/2024 1:35:17 PM
Method	ESI_pos_20181024	Operator	Bruker_PC
Sample Name	F-OMe	Instrument	apex-IV
Comment	CHCl3+ACN		

Acquisition Parameter

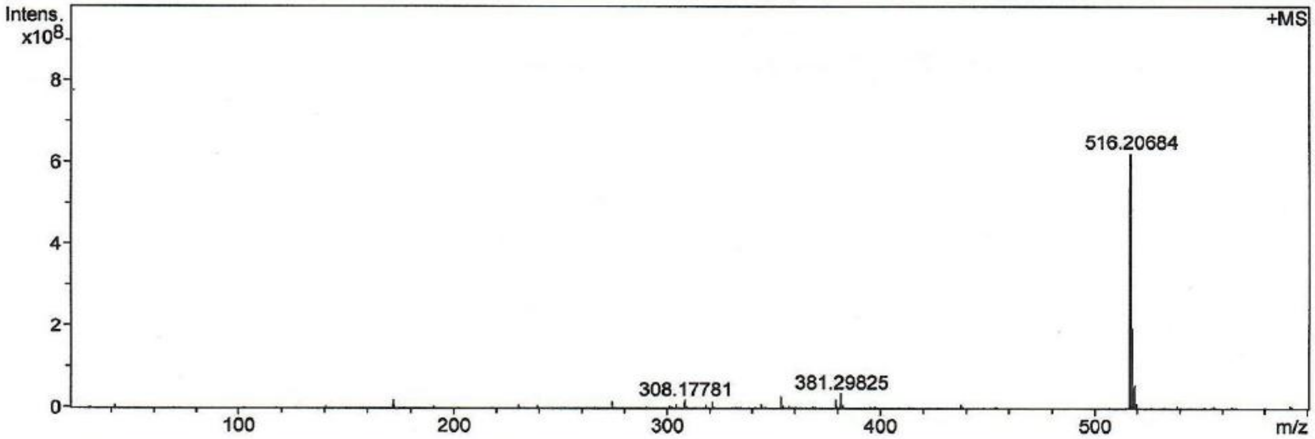

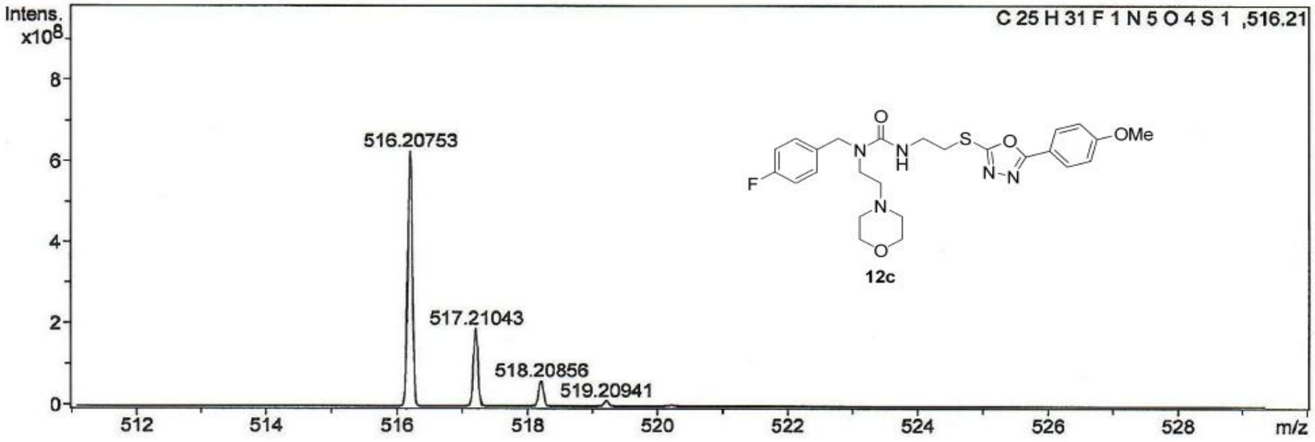

Sum Formula	Sigma	m/z	Err [ppm]	Mean Err [ppm]	Err [mDa]	rdb	N Rule	e⁻
C 25 H 31 F 1 N 5 O 4 S 1	0.007	516.20753	1.34	0.18	0.69	12.50	ok	even

Bruker Daltonics DataAnalysis 3.4 printed: 5/22/2024 1:37:28 PM Page 1 of 1

Figure 25. Mass spectroscopy of high resolution for compound **12c**

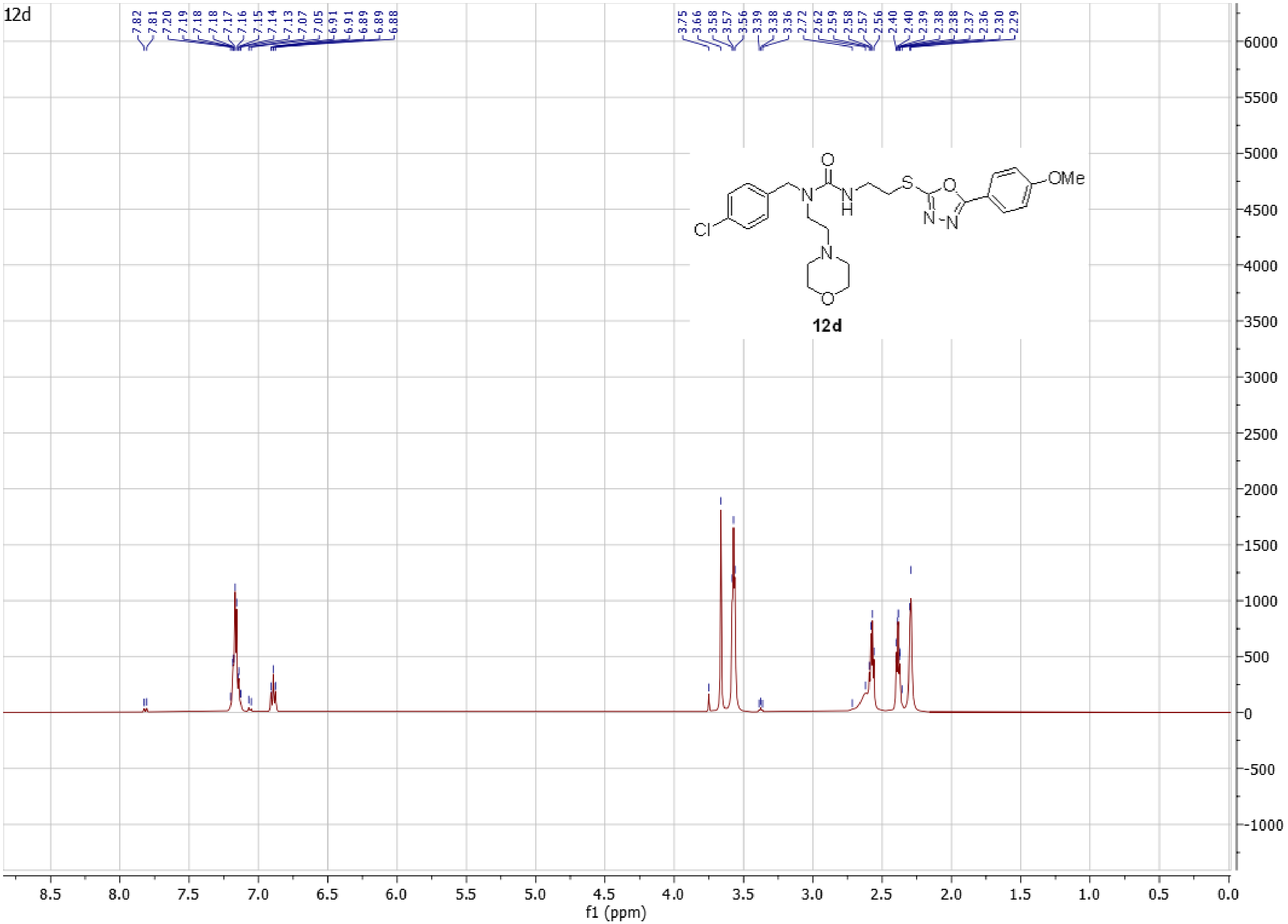

Figure 26. 1H-NMR for compound **12d**

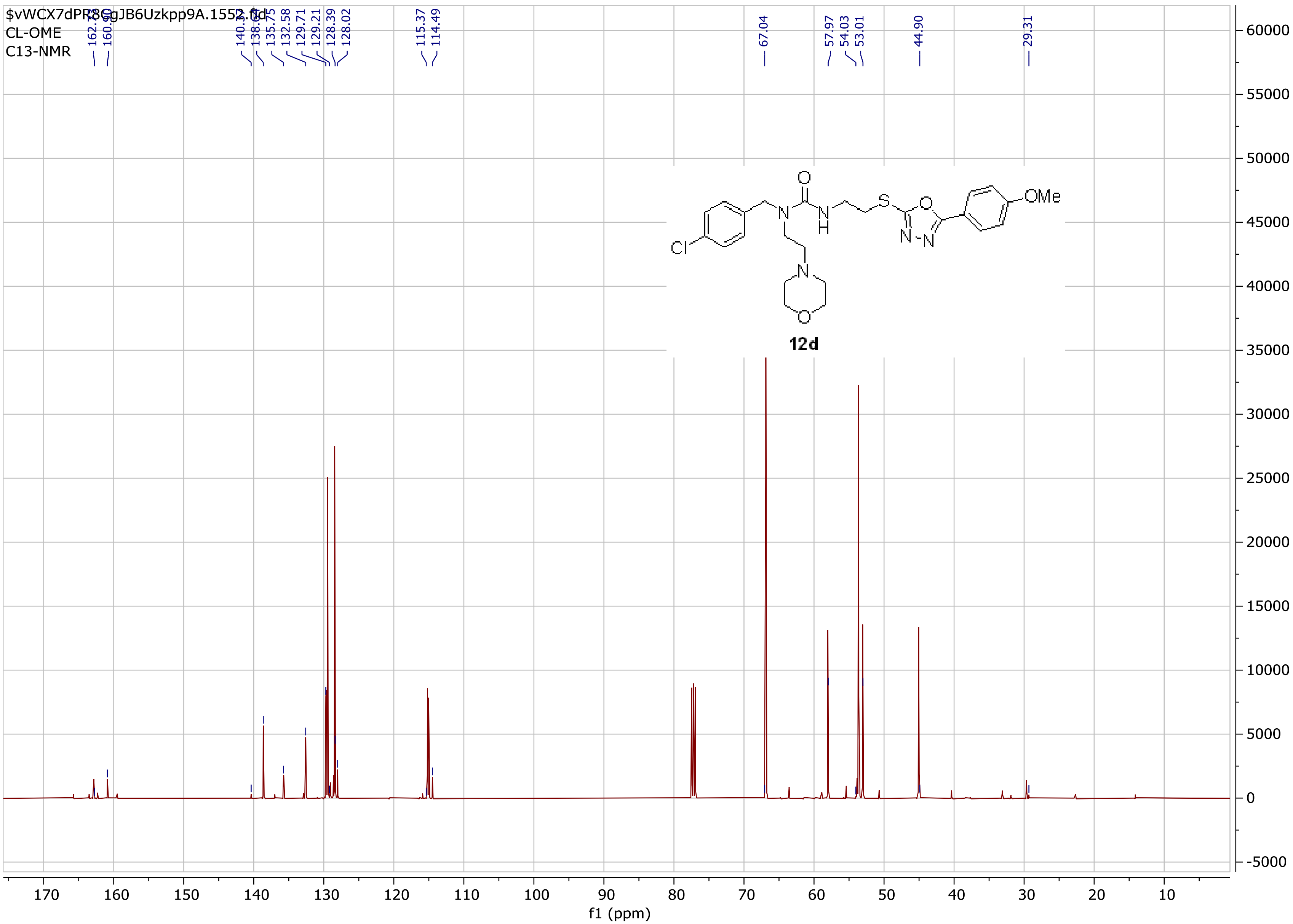

Figure 27. 13C-NMR for compound **12d**

Mass Spectrum Molecular Formula Report

Analysis Info

Analysis Name	F:\Data\2024MAY23\Mustafa_000063.d	Acquisition Date	7/4/2024 12:37:58 PM
Method	ESI_pos_20181024	Operator	Bruker_PC
Sample Name	Cl-OMe	Instrument	apex-IV
Comment	CHCl3+ACN		

Acquisition Parameter

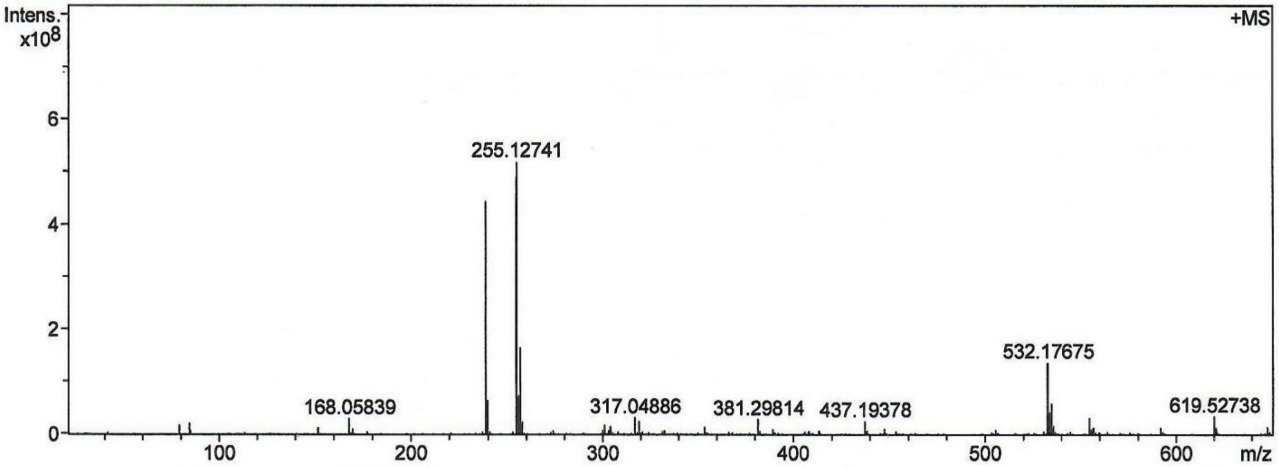

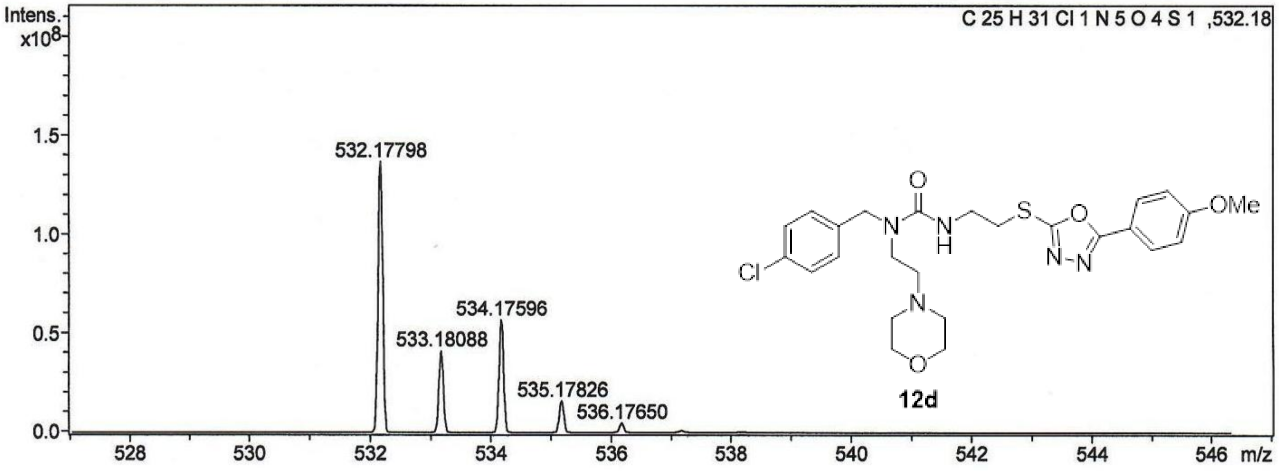

Sum Formula	Sigma	m/z	Err [ppm]	Mean Err [ppm]	Err [mDa]	rdb	N Rule	e⁻
C 25 H 31 Cl 1 N 5 O 4 S 1	0.008	532.17798	2.30	1.55	1.22	12.50	ok	even

Figure 28. Mass spectroscopy of high resolution for compound **12d**

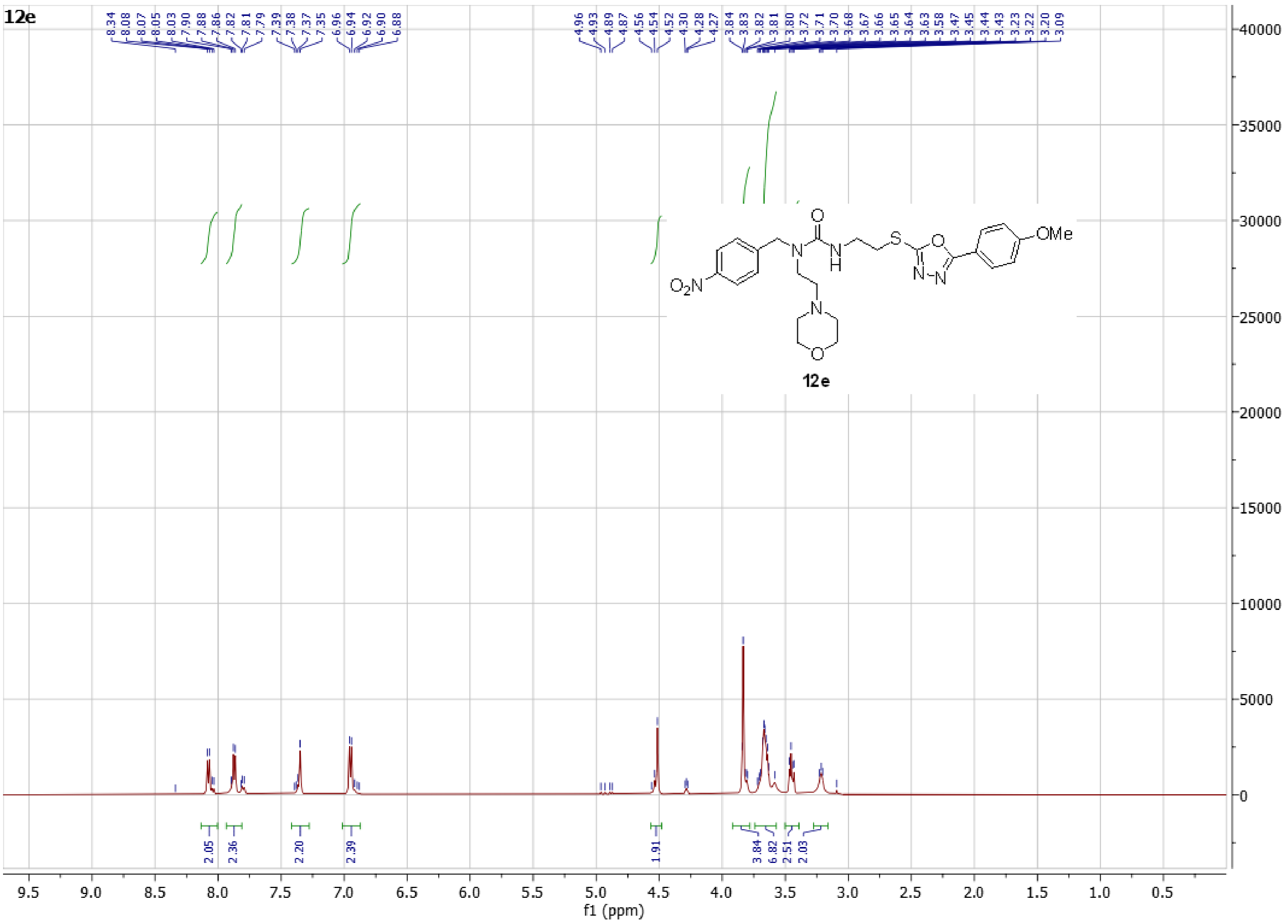

Figure 29. 1H-NMR for compound **12e**

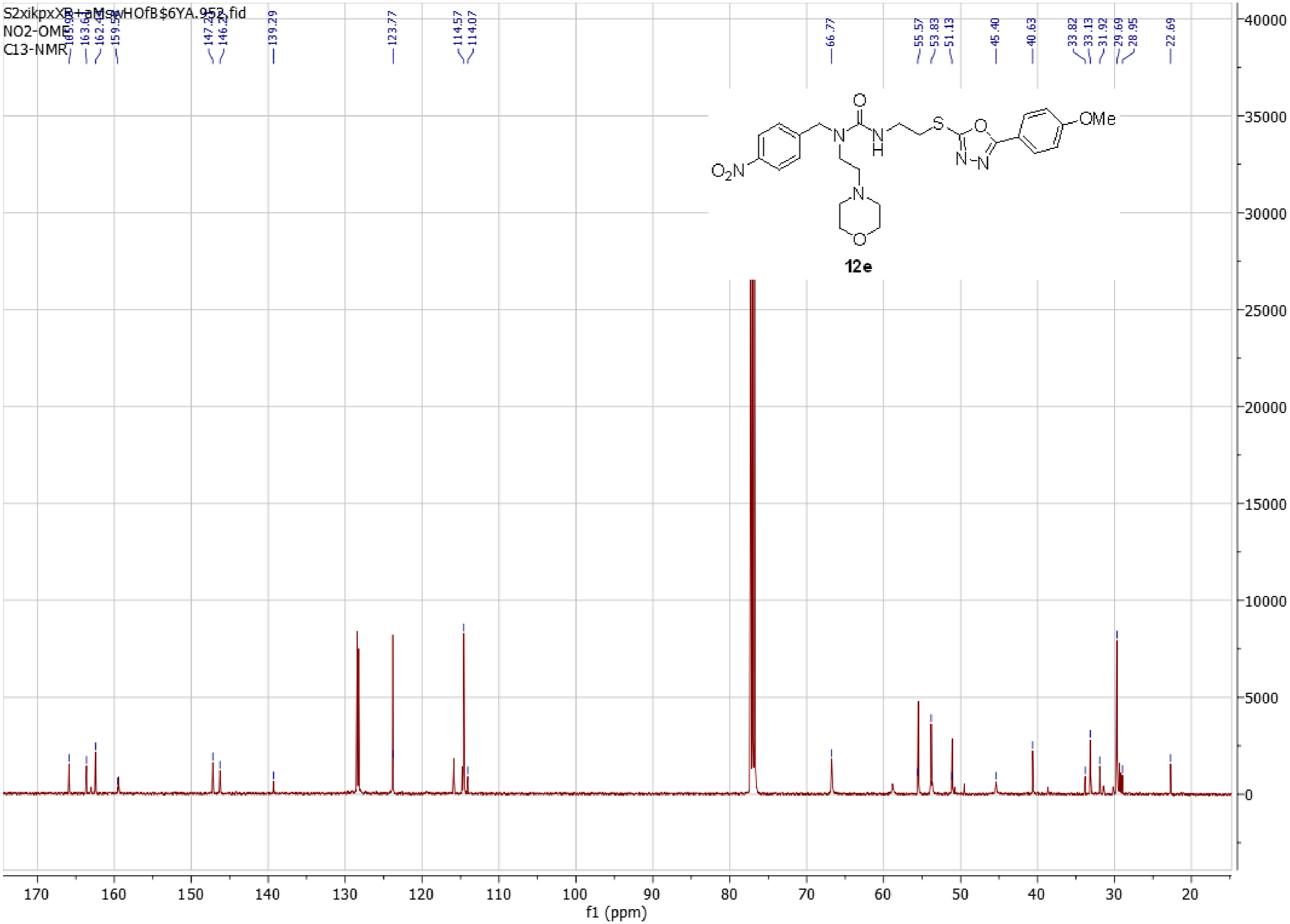

Figure 30. 13C-NMR for compound **12e**

Mass Spectrum Molecular Formula Report

Analysis Info | Acquisition Date 7/4/2024 12:27:42 PM

Analysis Name	F:\Data\2024MAY23\Mustafa_000061.d		
Method	ESI_pos_20181024	Operator	Bruker_PC
Sample Name	NO2-OMP	Instrument	apex-IV
Comment	CHCl3+ACN		

Acquisition Parameter

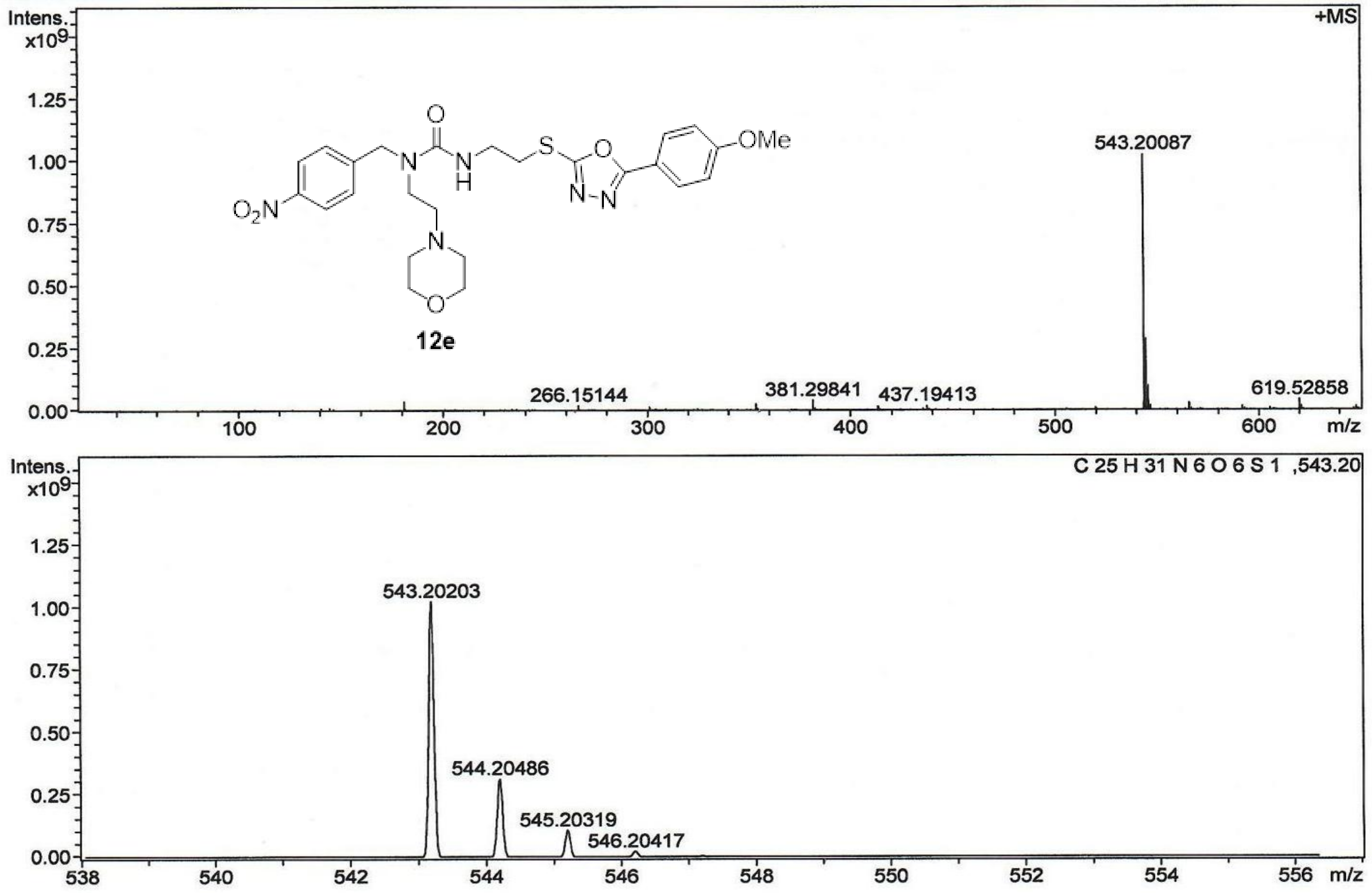

Sum Formula	Sigma	m/z	Err [ppm]	Mean Err [ppm]	Err [mDa]	rdb	N Rule	e⁻
C 25 H 31 N 6 O 6 S 1	0.012	543.20203	2.13	2.35	1.16	13.50	ok	even

Figure 31. Mass spectroscopy of high resolution for compound **12e**

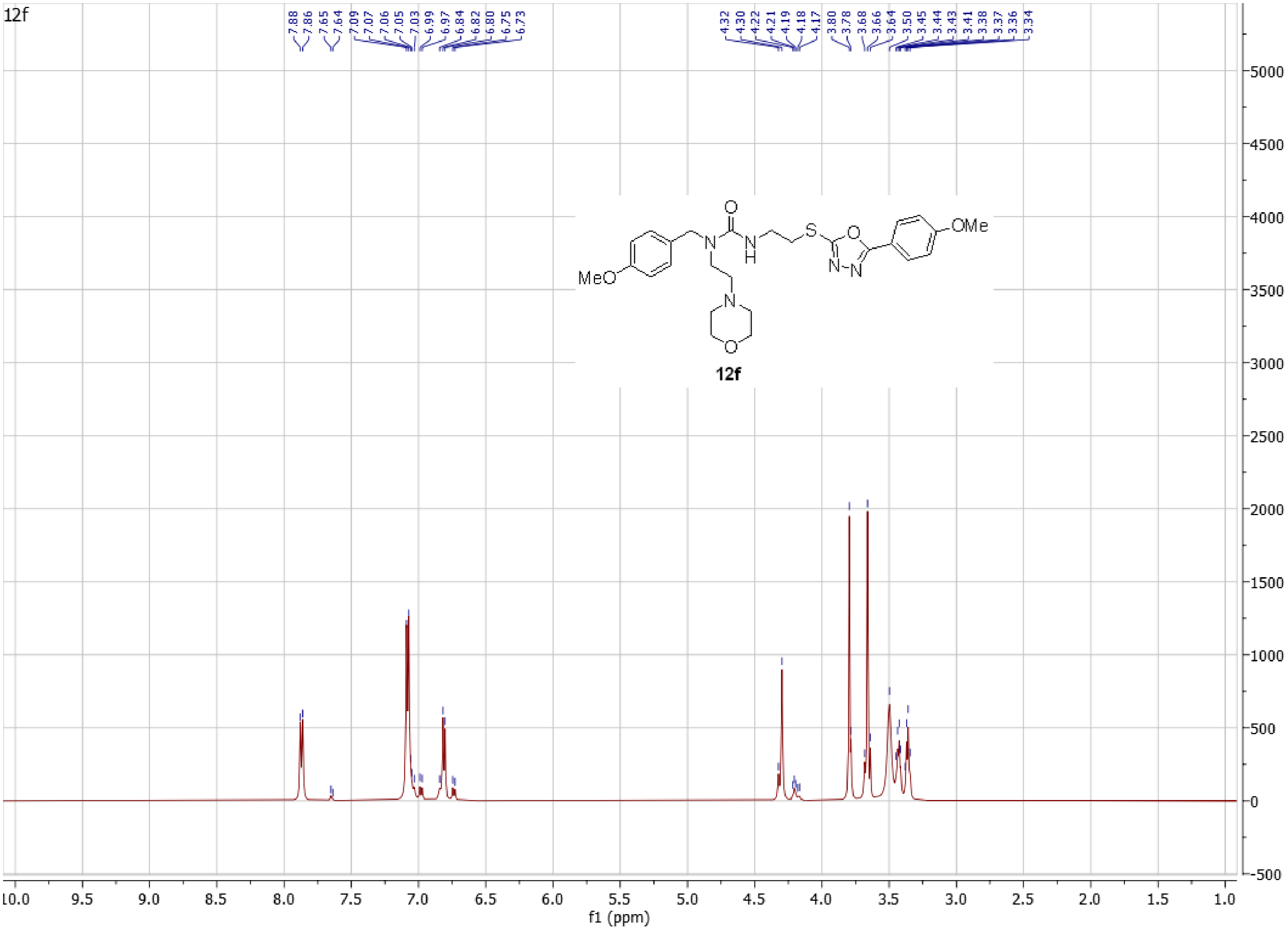

Figure 32. 1H-NMR for compound **12f**

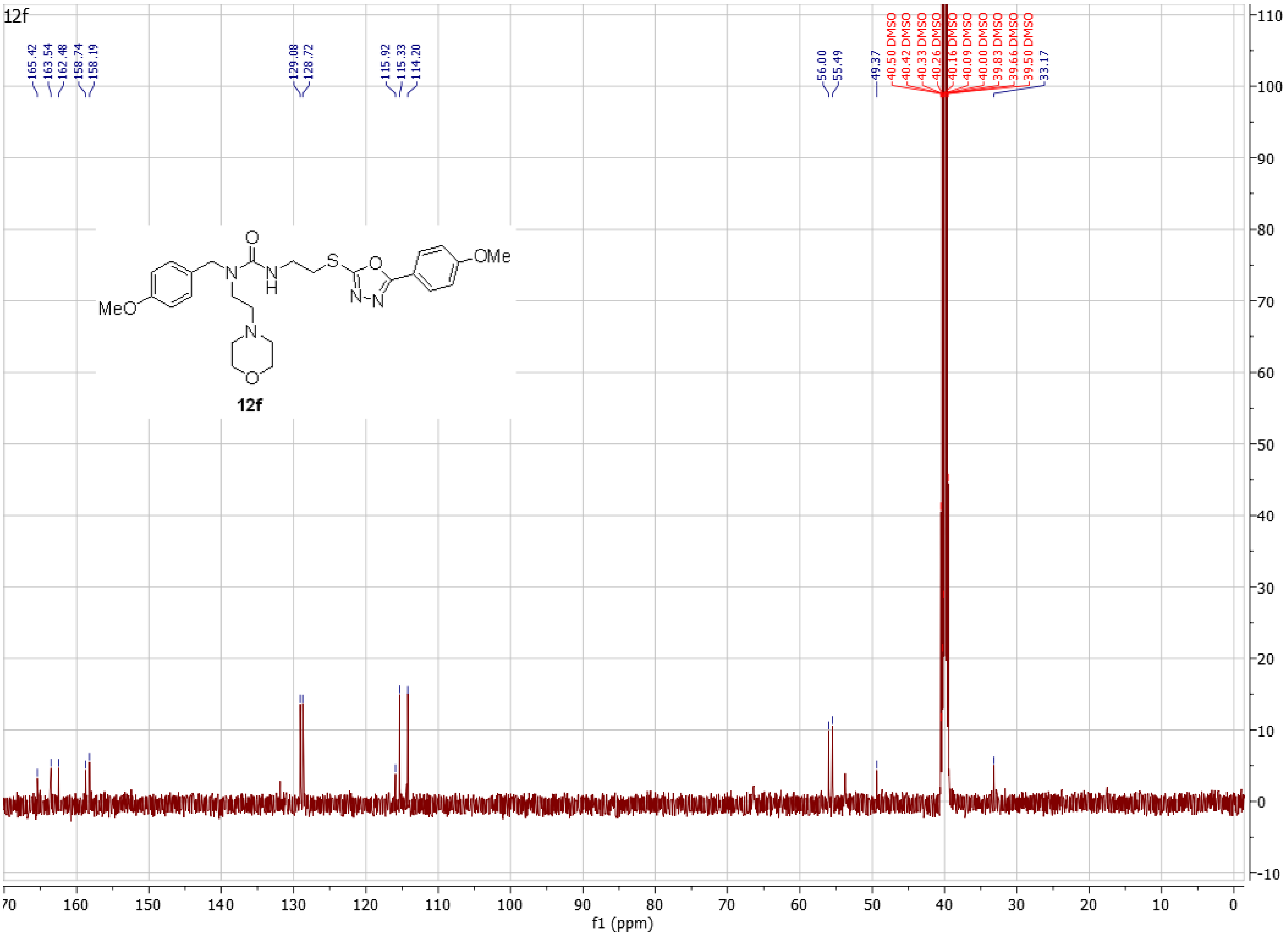

Figure 33. 13C-NMR for compound **12f**

Mass Spectrum Molecular Formula Report

Analysis Info

Analysis Name	F:\Data\2024JUL10\Mustafa_000015.d	Acquisition Date	7/18/2024 12:42:34 PM
Method	ESI_pos_20181024	Operator	Bruker_PC
Sample Name	Anis-OMe	Instrument	apex-IV
Comment	CHCl3+ACN		

Acquisition Parameter

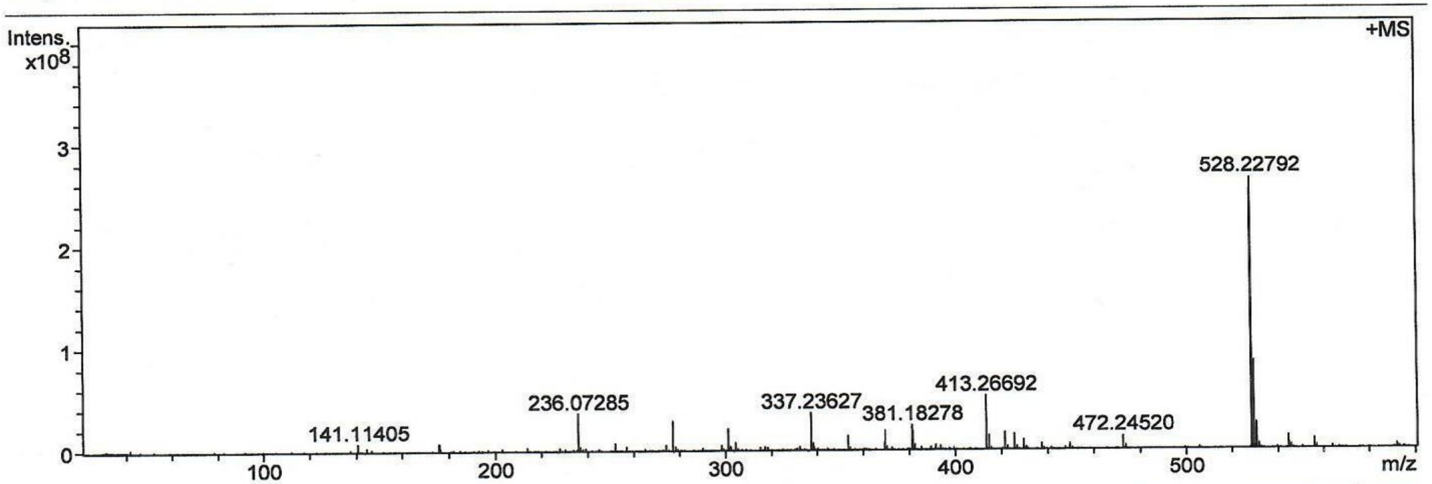

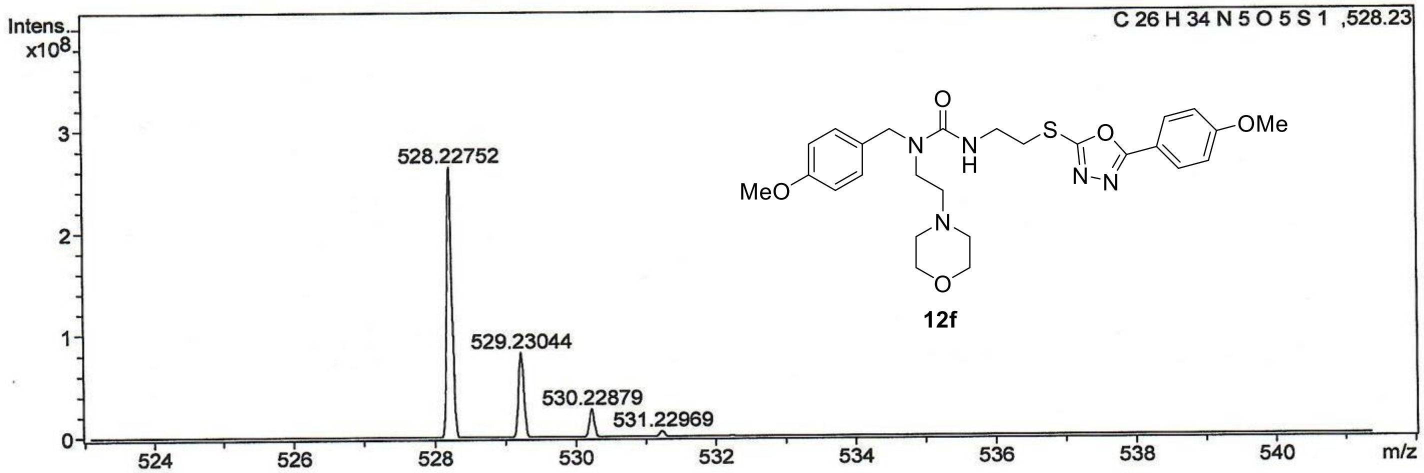

Sum Formula	Sigma	m/z	Err [ppm]	Mean Err [ppm]	Err [mDa]	rdb	N Rule	e⁻
C 26 H 34 N 5 O 5 S 1	0.007	528.22752	-0.77	-1.81	-0.41	12.50	ok	even

Figure. 34. Mass spectroscopy of high resolution for compound **12f**

Mass Spectrum Molecular Formula Report

Analysis Info

Analysis Name	F:\Data\2024APR23\Mustafa_000042.d	Acquisition Date	5/6/2024 10:07:04 AM
Method	ESI_pos_20181024	Operator	Bruker_PC
Sample Name	V-OMe	Instrument	apex-IV
Comment	CHCl3+ACETONE		

Acquisition Parameter

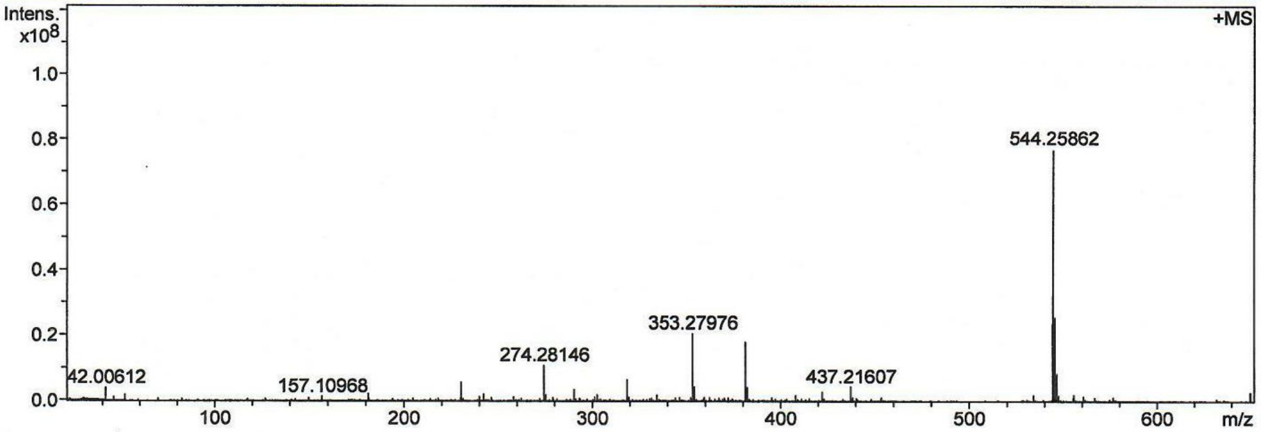

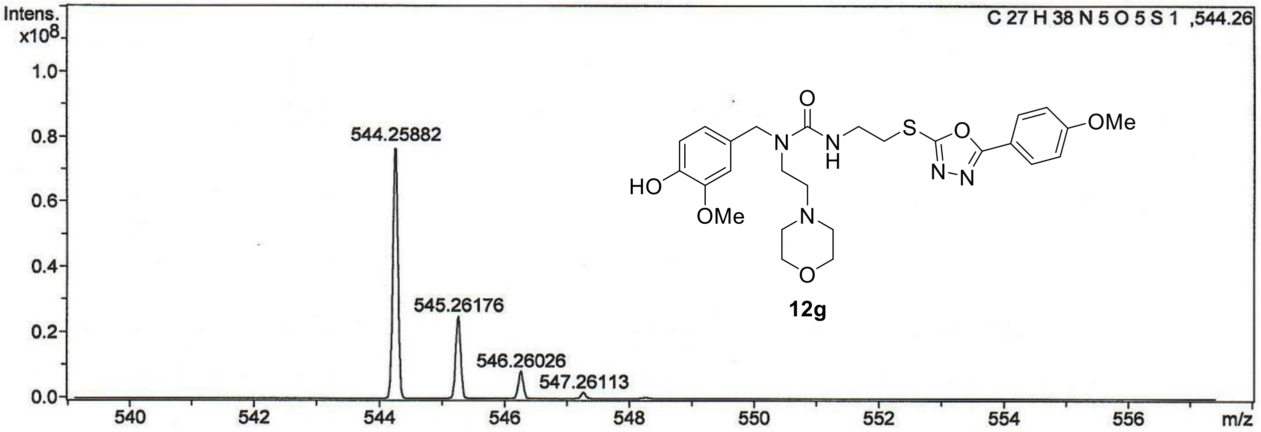

Sum Formula	Sigma	m/z	Err [ppm]	Mean Err [ppm]	Err [mDa]	rdb	N Rule	e⁻
C 27 H 38 N 5 O 5 S 1	0.005	544.25882	0.36	-0.17	0.20	11.50	ok	even

Figure 35. Mass spectroscopy of high resolution for compound **12g**

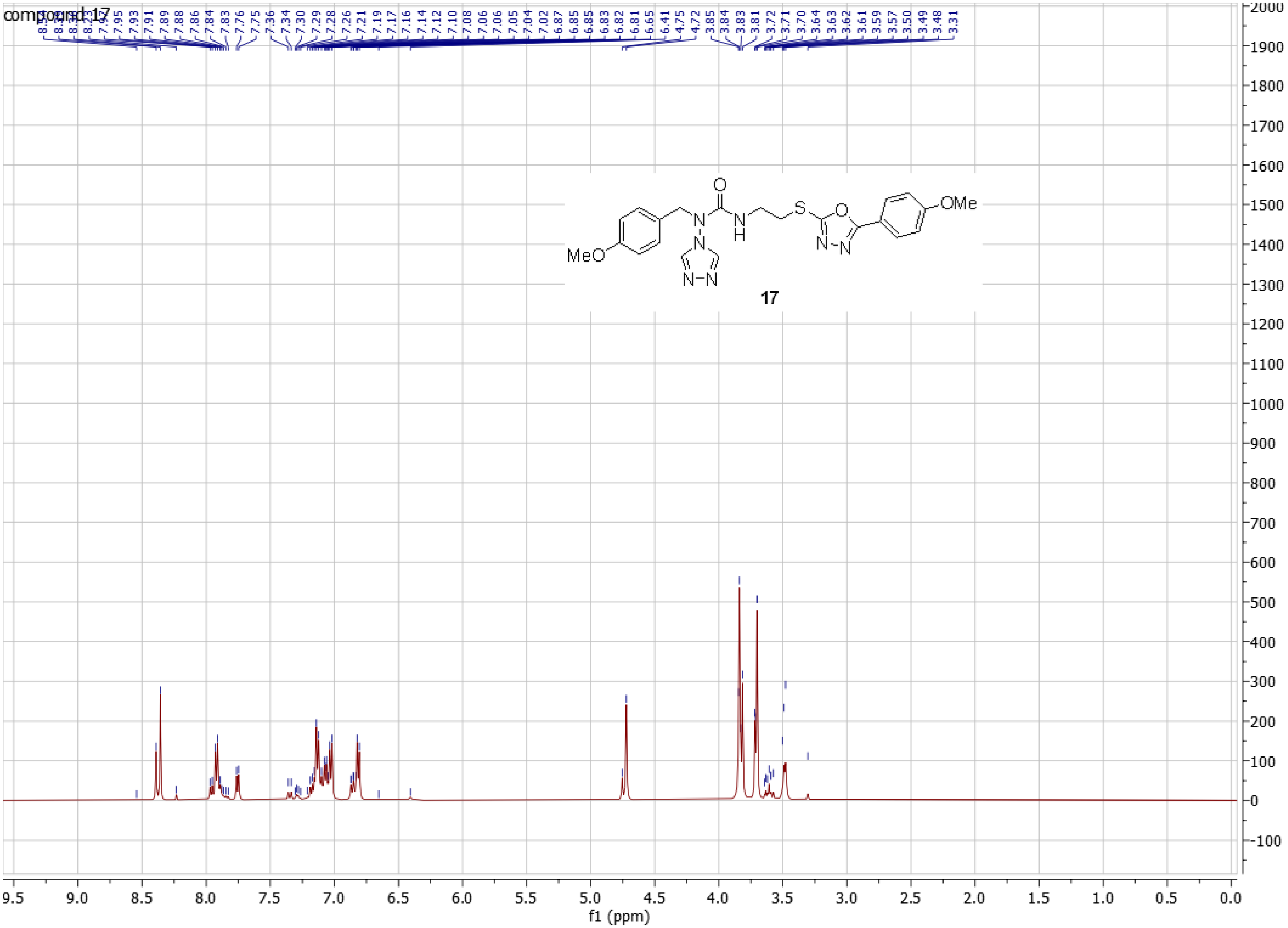

Figure 36. 1H-NMR for compound **17**

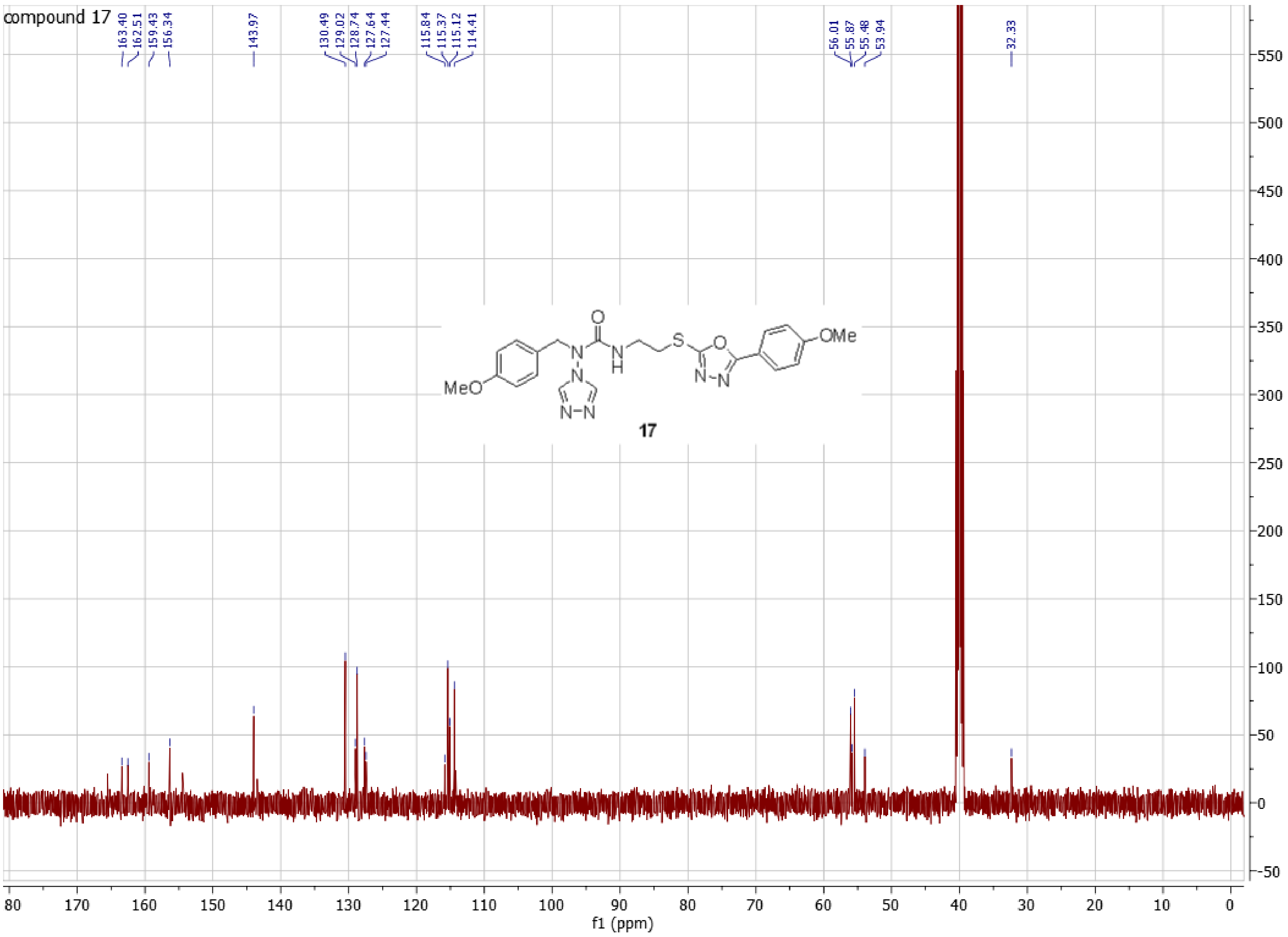

Figure 37. 13C-NMR for compound **17**

Intermediates NMR

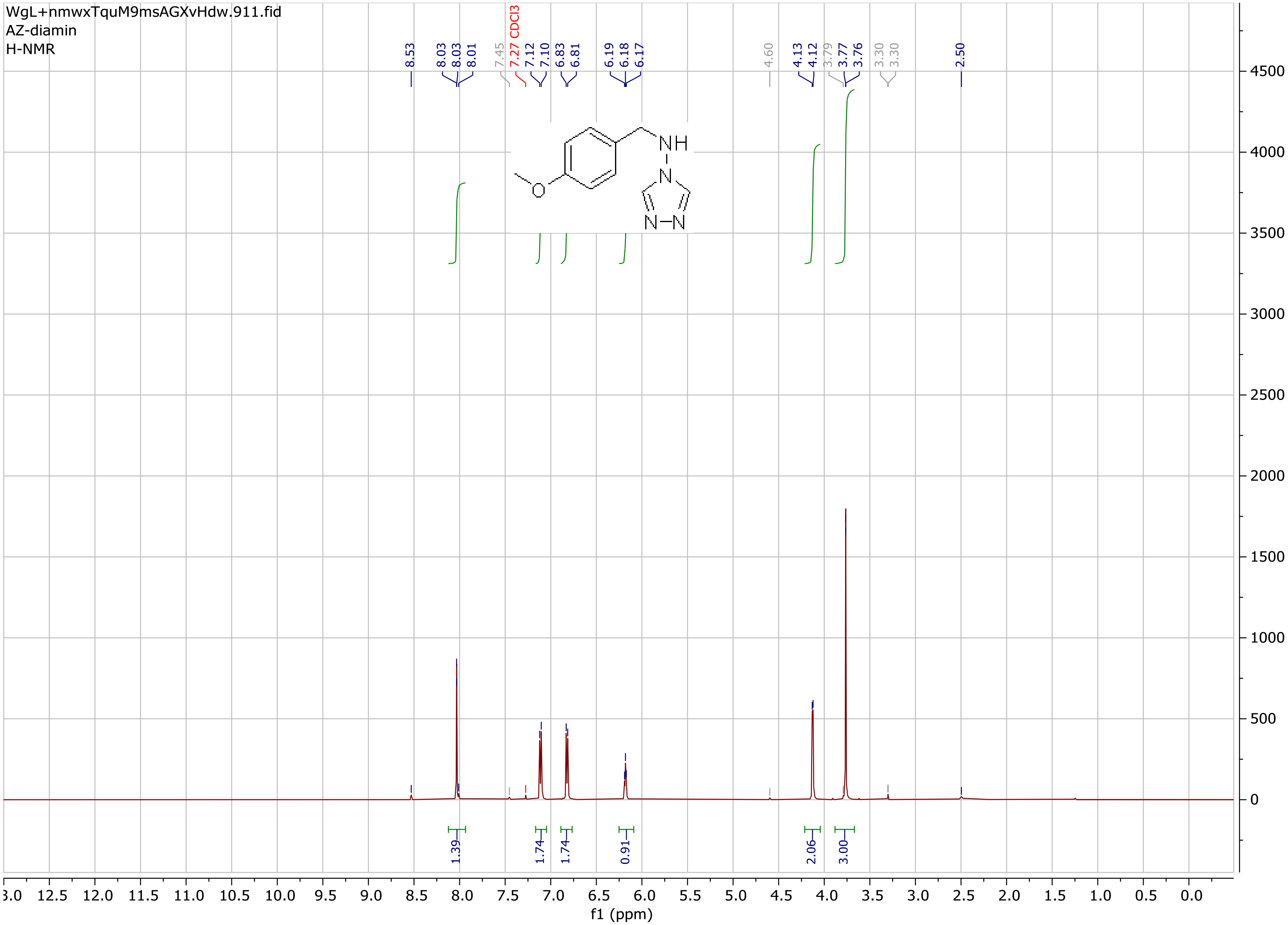

Figure 38. 1H-NMR for compound **15**

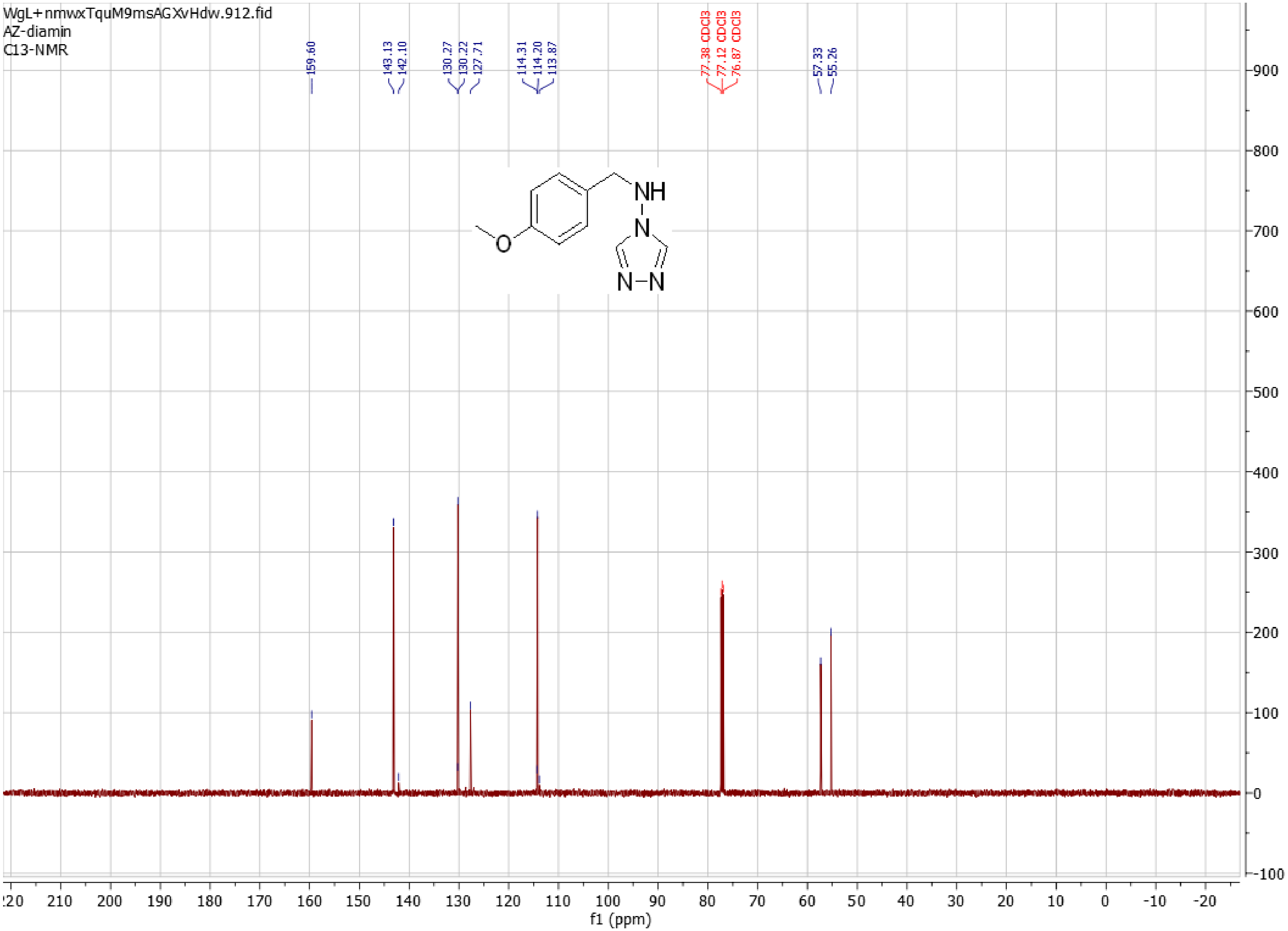

Figure 39. 13C-NMR for compound **15**

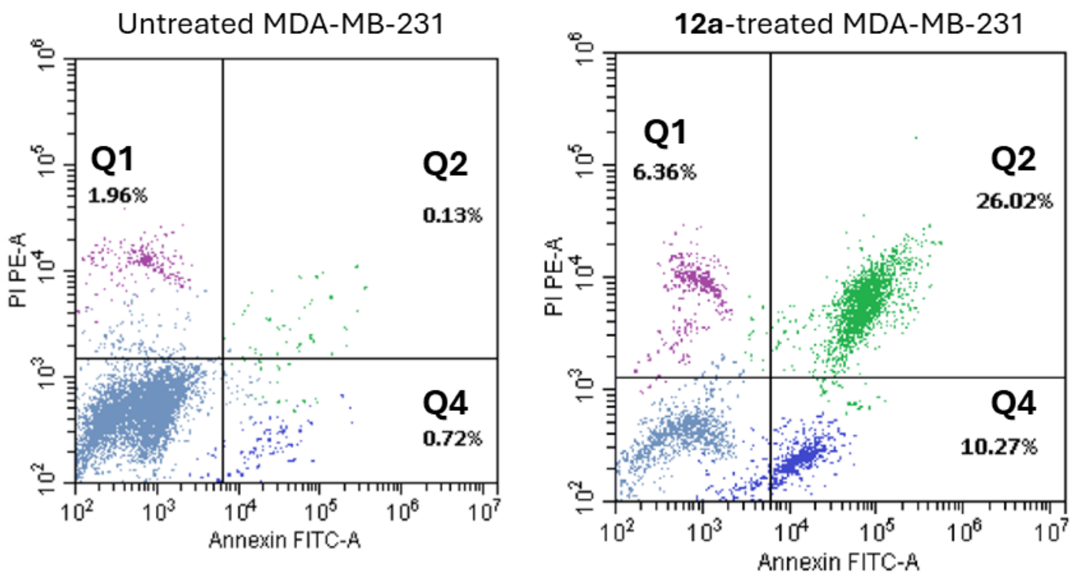

Figure 40: Histogram of Annexin V/PI staining in the untreated and 12a-treated MDA-MB-231 cancer cells

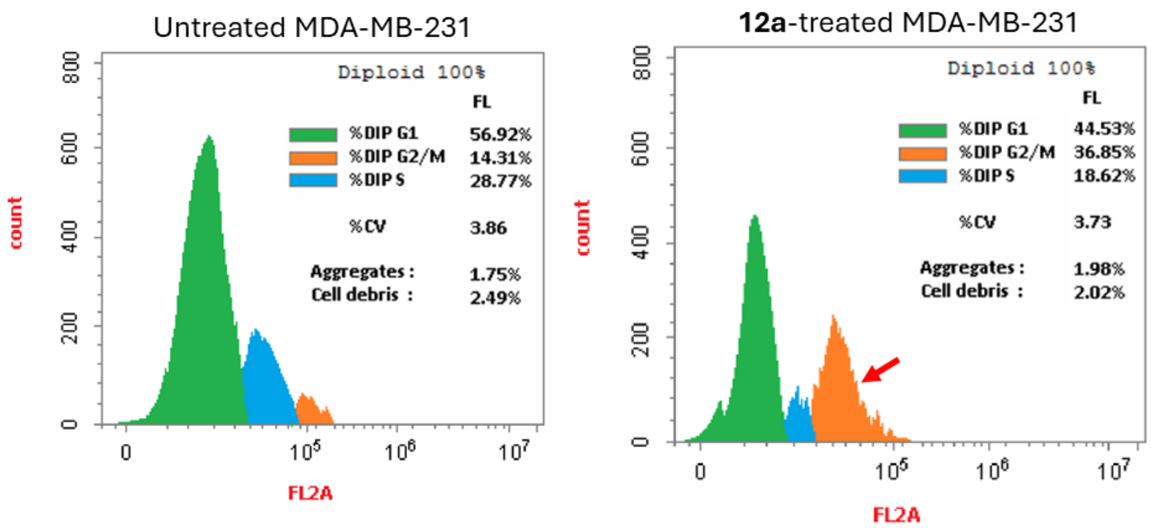

Figure 41: Histogram of cell cycle analysis in the untreated and 12a-treated MDA-MB-231 cancer cells
